# PemK’s Arg24 is a crucial residue for PemIK toxin–antitoxin system to induce the persistence of *Weissella cibaria* against ciprofloxacin stress

**DOI:** 10.3389/fmicb.2024.1402319

**Published:** 2024-05-14

**Authors:** Hao-Yu Zhu, Wen-Liang Xiang, Ting Cai, Min Zhang, Han-Yang Wang

**Affiliations:** ^1^School of Food and Bioengineering, Xihua University, Chengdu, China; ^2^Key Laboratory of Food Microbiology of Sichuan, Xihua University, Chengdu, China

**Keywords:** *Weissella cibaria*, toxin-antitoxin system, PemIK module, ciprofloxacin stress, persister cell, metabolic processes

## Abstract

The toxin-antitoxin (TA) system plays a key role in bacteria escaping antibiotic stress with persistence, however, the mechanisms by which persistence is controlled remain poorly understood. *Weissella cibaria*, a novel probiotic, can enters a persistent state upon encountering ciprofloxacin stress. Conversely, it resumes from the persistence when ciprofloxacin stress is relieved or removed. Here, it was found that PemIK TA system played a role in transitioning between these two states. And the PemIK was consisted of PemK, an endonuclease toxic to mRNA, and antitoxin PemI which neutralized its toxicity. The PemK specifically cleaved the U↓AUU in mRNA encoding enzymes involved in glycolysis, TCA cycle and respiratory chain pathways. This cleavage event subsequently disrupted the crucial cellular processes such as hydrogen transfer, electron transfer, NADH and FADH_2_ synthesis, ultimately leading to a decrease in ATP levels and an increase in membrane depolarization and persister frequency. Notably, Arg_24_ was a critical active residue for PemK, its mutation significantly reduced the mRNA cleavage activity and the adverse effects on metabolism. These insights provided a clue to comprehensively understand the mechanism by which PemIK induced the persistence of *W. cibaria* to escape ciprofloxacin stress, thereby highlighting another novel aspect PemIK respond for antibiotic stress.

## 1 Introduction

The discovery of antibiotics in the late 1920s marked a significant milestone in the history of combating bacterial infection. However, it is now widely acknowledged that antibiotics are not the panacea they were once thought to be. Rather, antibiotics can and do fail, often with severe consequences (Page and Peti, 2016). This occurs because bacteria acquire resistance mutations to render the antibiotics ineffective or activate their endogenous mechanisms to evade antibiotic stress (Page and Peti, 2016). The former is a major culprit, but it would be overly simplistic to attribute all failures solely to this factor (Maisonneuve and Gerdes, 2014). In fact, when bacteria are treated with antibiotic, it is nearly impossible to kill them completely. Even if they are sensitive to antibiotic, there will always be a small percentage of cells known as persister cells that can withstand the lethal effect of antibiotic (Wilmaerts et al., 2019). Bacterial persistence is a state of dormancy induced by an endogenous mechanism, and unlike drug-resistant mutant, it does not proliferate in the presence of antibiotic, but, randomly in time, switches from a dormant state back to a growing state when antibiotic stress is relieved or removed. Therefore, it is generally accepted that persistence is an important reason for failure of antibiotic prevention or treatment of bacteria (Wilmaerts et al., 2019).

Persister process of bacteria is a survival strategy that allows populations to survive in sudden adverse environments without going extinct completely (Gerdes and Maisonneuve, 2012; Page and Peti, 2016; Wilmaerts et al., 2019). To date, persistence has been observed in nearly all bacterial species, even eukaryotic microorganisms (Wilmaerts et al., 2019). Although persister process is governed by phenotypic switching in isogenic populations, it occurs stochastically and independently of environment, albeit at a higher frequency in adverse conditions (Maisonneuve and Gerdes, 2014; Page and Peti, 2016; Wilmaerts et al., 2019). Numerous researchers have suggested that persister formation depends on the expression level of toxin-antitoxin systems (TAs) in bacteria. These systems encode two components, a stable toxin protein that inhibits cell growth and a labile antitoxin (either RNA or protein) that regulates toxin activity. When toxin protein exceeds a certain threshold in bacterial cell, persistence is induced (Wilmaerts et al., 2019).

PemIK is a novel type II TA system, originally discovered in plasmid pCH91 of *Staphylococcus aureus* (Bukowski et al., 2013). The toxin PemK dimer, an RNase, specifically cleaves the mRNA in a ribosome-independent manner. The antitoxin PemI neutralizes the toxicity of PemK, thereby facilitating bacterial recovery from persistence (Bukowski et al., 2013; Wilmaerts et al., 2019). In pathogenic bacteria such as *S. aureus*, *Klebsiella pneumonia*, *Bacillus anthracis* and *Mycobacterium tuberculosis*, free PemK dimers lead to a rapid formation and higher proportion of persister cells (Kim et al., 2022). Although PemK can induce persister cells, it possesses distinct pattern in regulating physiological activities across different organisms. In *S. aureus*, it comprehensively regulates virulence by cleaving target mRNA encoding proteins involved in virulence synthesis (Bukowski et al., 2013; Kim et al., 2022). However, in *Caenorhabditis elegans*, it affects fundamental physiological processes including ATP production, lipid synthesis, cytoskeleton organization, stress response, and others (Kim et al., 2022). However, the mechanism by which PemK orchestrates the specific regulation of bacterial transcription, thereby manipulating the transition from growth to persistence, remains elusive.

*Weissella cibaria*, a novel probiotic and hetero-lactic bacterium, is usually present in various spontaneous fermentation foods. Interestingly, it can survive in various stresses through persistence, including antibiotic residue in raw materials, high acidity and nutrient hunger, and even growth inhibitor. In its persister cells, PemIK responds strongly to the persister formation under ciprofloxacin stress. Although the structure and function of PemIK in pathogenic bacteria, particularly *S. aureus*, are somewhat known, it remains unknown in *W. cibaria* due to its species-specific mode of regulation. Therefore, the present study analyzed the structure and function of PemIK in *W. cibaria*, and investigated the PemK’s adverse effects on basic metabolic processes. Meanwhile, the detoxification effect of PemI on PemK was also determined. The results provided a structural basis for understanding the molecular mechanism by which PemIK induced persistence of *W. cibaria* to survive in ciprofloxacin stress, and also offered insights into strategies for discovering new agents that could activate or inhibit *W. cibaria*.

## 2 Materials and methods

### 2.1 Bacterial strains and culture conditions

*Weissella cibaria* CGMCC 1.19376 was cultured in De Man, Rogosa, and Sharpe (MRS) broth at 30°C (Cai et al., 2022). *Escherichia coli* BL21 was cultivated in Luria-Bertani (LB) liquid me-dium at 37°C.

### 2.2 Transcription analysis of PemIK

Total RNA was extracted using TRIzol Reagent (ThermoFisher Scientific Inc., Massachusetts, USA). Subsequently, cDNA synthesis was performed using the prime-script reverse transcriptase and random primers according to manufacturer’s instructions (TaKaRa, Beijing, China). Polymerase chain reaction (PCR) was conducted with PemK-F, PemK-R, PemI-F, and PemI-R primers (Supplementary Table 1) for amplifying the PemI and PemK genes. Templates included cDNA, mRNA, and gDNA (genomic DNA of *W. cibaria*). The promoter and terminator analysis of PemKI were performed by BPROM program (Shahmuradov, 2003).

### 2.3 Structural analysis of PemK

The primary and secondary structures of PemK were analyzed by Clustal Omega and Jalview,^1^ respectively. The results were visualized by ESPript 3.0 (Robert and Gouet, 2014). Three-dimensional models of PemK protein were generated using SWISS-MODEL (Waterhouse et al., 2018). The dimeric structure of PemK was submitted along with mRNA containing 5′ UAUU 3′ to the HADDOCK 2.2 webserver (Van Zundert et al., 2016). Subsequently, a model of PemK bound to RNA was generated and visualized by PyMOL (PyMOL Molecular Graphics System, version 2.3.0) (Schiffrin et al., 2020).

### 2.4 Identification of active residues in PemK

The SNAP2^2^ predicted the mutation of active amino acid in PemK (Bromberg and Rost, 2007). The thermal stability, solvent-accessible surface area and electrostatic potential energy of PemK were analyzed by MUpro,^3^ CCP4^4^ and APBS plug in PyMOL, respectively.

### 2.5 Cytotoxicity analysis of PemIK

PemK and its mutant were ligated to pET28a plasmid, while PemI was ligated to pBAD43 plasmid. Subsequently, the recombinant pET28a and pBAD43 were co-transformed into *E. coli* BL21. Following cultivation to an optical density (OD) 600 nm of 0.2–0.3, PemK and PemI were induced by 1 mM IPTG and 0.2% L-arabinose, respectively. Cytotoxicity of PemK and neutralization of PemK by PemI were assessed through growth curves of *E. coli* BL21 at OD_600*nm*_.

### 2.6 Regulation of cell membrane potential, endogenous reactive oxygen species (ROS) and intracellular ATP by PemK

*Weissella cibaria* at 1 × 10^7^ CFU/ml in logarithmic stage was treated with 1/2 MIC ciprofloxacin (16 μg/ml) at 30°C for 1–8 h. Recombinant *E. coli* BL21 was cultured to logarithmic stage and adjusted to 1 × 10^7^ CFU/ml, followed by induction of PemK using 1 mM IPTG at 37°C for 1–8 h. The cell membrane potential was assessed using the rhodamine 123 fluorescence method (Yu et al., 2022). The endogenous ROS level was analyzed following the method described by Xiong et al. (2023). Intracellular ATP was determined as described by Shan et al. (2017). PBS (pH 7.4) treatment served as control.

### 2.7 Effect of PemK on expression of metabolic enzymes

The splicing mRNA of PemK were analyzed based on the genome sequence of *W. cibaria*, focusing on metabolic enzymes in oxidative phosphorylation (OP), glycolysis, and citric acid cycle (TCA). To further validate these impacts, transcriptome analysis was conducted in recombinant *E. coli* BL21. The total RNA with high-quality from recombinant *E. coli* BL21 using TRIzol Reagent (ThermoFisher Scientific Inc., MA, USA) was used to construct the RNA-Seq library for sequencing on Illumina Hiseq platform (Hiseq 4000, 150PE) with NEBNext^®^ Ultra™ RNA Library Prep Kit for Illumina^®^ (NEB, USA) by Majorbio Technology Co. Ltd (Shanghai, China). The RNA-seq data analysis was conducted as methods described by Wang et al. (2015). The differential expression was calculated by DESeq R package (1.18.0). The transcription with log_2_ fold change > 1 was defined as the metabolic enzymes affected by PemK.

### 2.8 Regulation of cell persistence and morphology by PemK

The persister cells were detected using method of biphasic bactericidal curve (Sun et al., 2017). Briefly, *W. cibaria* was incubated in MRS broth at 30°C to 10^7^ CFU/ml, then ciprofloxacin was added to 1/2 MIC and incubated again for 0, 1, 3, 5, 7 and 9 h. Subsequently, 0.5 ml cultures were transferred into 20 ml MRS broth containing MBC (2,048 μg/ml) ciprofloxacin and incubated at 30°C for 24 h. The 1 ml cultures were harvested by centrifugation at 4,000 rpm for 1 min and washed twice with PBS (pH 7.4). After cell pellets were resuspended and serially diluted 10-fold with PBS (pH 7.4), the dilution cells were inoculated on MRS agar plate to count after 24 h incubation at 30°C. The persister frequency was expressed as the ratio of viable bacteria in treated and untreated group with 1/2 MIC ciprofloxacin. After PemK was induced by IPTG in recombinant *E. coli* BL21, no-persister cells were killed with MBC ciprofloxacin in LB broth medium. The persister frequency of recombinant *E. coli* BL21 was carried out following similar procedures described above. The cell morphology was observed in FEI inspect F50 scanning electron microscope (Inspect F50, FEI, Hillsboro, USA).

### 2.9 Statistical analysis

All experiments were conducted in triplicate for each sample, and the data was presented as the mean values ± standard deviation, with variations less than 10%. Statistical significance analysis was carried out using analysis of variance (ANOVA) with Tukey’s *post-hoc* test in SPSS 20.0, and a *p*-value less than 0.05 was considered statistically significant.

## 3 Results

### 3.1 PemIK is a typical II TA stress regulator

**FIGURE 1:**
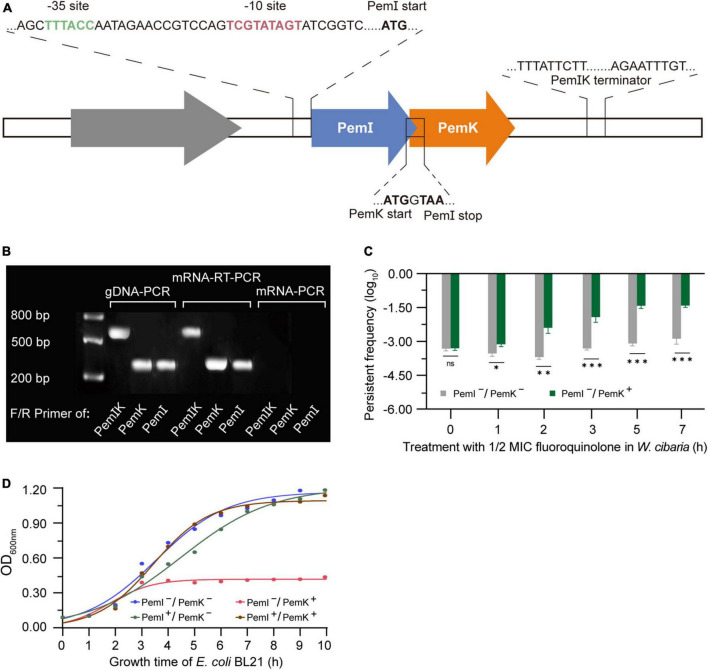
The structural characteristics and pressure response mechanism of PemIK. **(A)** Operon structure of PemIK of *W. cibaria*. **(B)** Validation of co-transcription. **(C)** Persister frequency of *W. cibaria* after treatment with 1/2 MIC ciprofloxacin. **(D)** Growth curve of recombinant *E. coli* BL21, demonstrating that PemI counteracted the cytotoxic effects of PemK through physical binding. Data were assessed by ANOVA with Tukey’s *post-hoc* test. The asterisk (*) indicated significant level: *P < 0.05, **P < 0.01, ***P < 0.001, not significant (ns), *P* > 0.05.

Co-localization and co-transcription in an operon are common characteristics of type II-TAs (Zhou et al., 2021). In *W. cibaria*, the PemI and PemK genes were situated at locus 8,267–8,949 on plasmid PA010 (Supplementary Figure 1), sharing a promoter and terminator in transcription but possessing independent start and stop codons in translation (Figure 1A). Both open reading frames (ORFs) consisted of a frame with 342 bp encoding 114 amino acids followed by a TAA termination codon. Interestingly, a genetic overlap of seven base pairs between them (Figure 1A), which distinguished it from other PemIK structures.

When *W. cibaria* was exposed to 1/2 MIC ciprofloxacin, the co-transcription of PemIK was activated (Figure 1B), while PemI underwent degradation by Lon enzyme. Consequently, free PemK exhibited cytotoxicity to *W. cibaria*, leading to a severe growth inhibition and a significant increase in proportion of persister cells (Figure 1C). It has been demonstrated that PemI could counteract the cytotoxicity of PemK in recombinant *E. coli* BL21 (Figure 1D). Moreover, it also functioned as a transcription factor by inhibiting their co-transcription. Therefore, once the adverse conditions were alleviated or eliminated, the persistence of *W. cibaria* resumed its growth.

### 3.2 PemK is a novel specific endonuclease of mRNA

**FIGURE 2:**
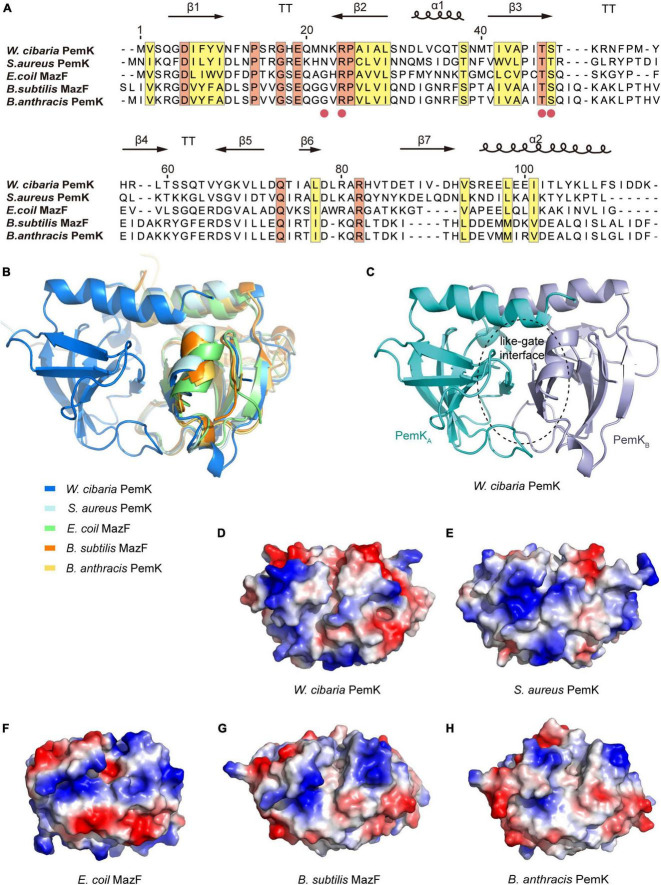
The structural comparison of PemK with its homologs. **(A)** Sequence alignment of PemK with its homologs. Secondary structures were denoted in the upper region. Potential active site residues are marked as red circles. Conserved and similar residues were shown in orange and yellow boxes, respectively. α: α-helix, β: β-sheet, T: turn. **(B)** Overlay structure of PemK with other homologs. **(C)** Overall structure of the PemK dimer. **(D–H)** Electrostatic surface potential represented the different charge distributions at the gate-like interface regions in PemK and its homologs.

The toxins of II-type TAs are generally enzymes, particularly RNases (Díaz-Orejas et al., 2017). PemK from *W. cibaria* shared conserved residues and exhibited similar folds with its homologs, a barrel-shaped pattern of 7 β-sheets and 2 α-helices (Figure 2A). However, there were some differences in their secondary structures (Figure 2B), suggesting that they have similar functions but different mechanisms in controlling mRNA binding. The binding of mRNA typically occurred at the like-gate interfaces, which corresponded to the crevices between β1–β2 and β3–β4 sheets of one monomer and α1 helices of the other monomer in PemK dimers from *W. cibaria* (Figure 2C). The gate of PemK dimer from *W. cibaria* differed from that observed in its homologs from *S. aureus*, *E. coli*, *B. subtilis*, and *B. anthracis* (Figure 2B). Simultaneously, its electrostatic potential surface also displayed variations compared to its homologs (Figures 2D–H), it featured a weak positive charge plaque on its surface. These unique structural differences suggested that its mechanism for binding and cleaving mRNA differed from its homologs.

**FIGURE 3:**
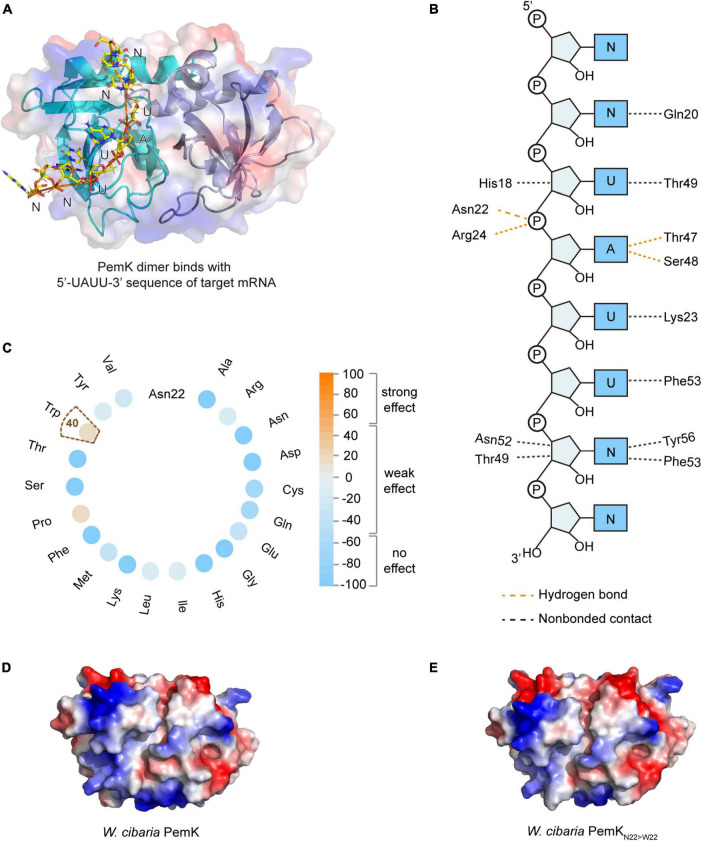
The mRNA binding and cleavage of PemK. **(A)** Model of PemK-mRNA complex depicted the stick model of PemK dimer and target mRNA. **(B)** NUCPLOT diagram of contacts between mRNA and PemK. Orange and black dotted lines represented hydrogen bonds and non-bonded contacts, respectively. **(C)** SNAP2 predicted the impact scores on the function of PemK after Asn_22_ (N_22_) was mutated to 20 amino acids, respectively. **(D)** Electrostatic surface potential of PemK. **(E)** Electrostatic surface potential of PemK_N22>W22_ that Asn_22_ was mutated to Trp_22_ (W_22_).

PemK specifically recognizes and cleaves the tetrad U↓AHU (where H is C, A, or U) sequence in target mRNAs (Zhang et al., 2004; Zorzini et al., 2016; Cho et al., 2017). In *S. aureus*, Arg_25_ and Thr_48_ had been confirmed as key active residues for PemK (Kim et al., 2022). In *W. cibaria*, the mRNA’s U↓AHU (where H is U) sequence bound to the like-gate interfaces of PemK dimer through four hydrogen bonds and four van der Waals contacts (Figures 3A, B). Specifically, Asn_22_ and Arg_24_ formed hydrogen bonds with the phosphate group between U↓A, while Thr_47_ and Ser_48_ bound to the adenine base of U↓A through hydrogen bonds (Figure 3B). However, among these residues, Asn_22_ was not highly conserved in homologs based on sequence alignment (Figure 2A), and its substitution with any amino acid had little impact on PemK’s function (Figure 3C). Even when Asn_22_ was mutated to Trp *in situ*, there were no changes observed in the electrostatic potential surface of its gate interface (Figures 3D, E). Therefore, Arg_24_, Thr_47_ and Ser_48_ might be important residues influencing mRNA-binding and catalytic activity.

### 3.3 Arg_24_ is crucial for the RNase activity of PemK

**FIGURE 4:**
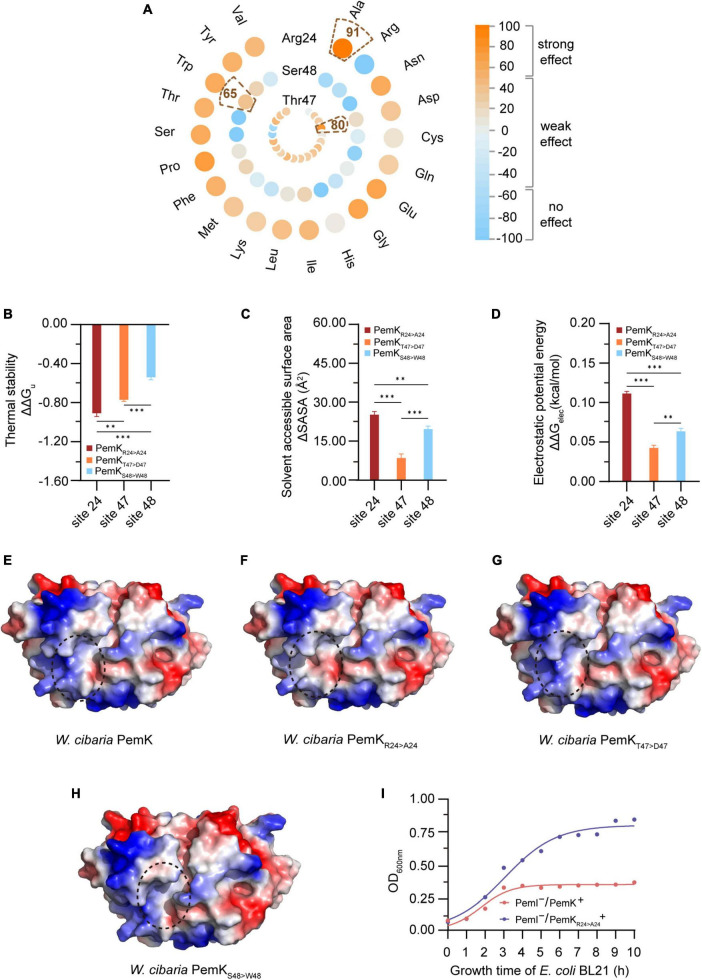
The properties of PemK after mutation of Arg_24_, Thr_47_ and Ser_48_. **(A)** SNAP2 predicted the impact scores on the function of PemK after Arg_24_, Thr_47_ and Ser_48_ were mutated to 20 amino acids, respectively. **(B–D)** Differences of thermal stability, solvent accessible surface area, electrostatic potential energy of PemK_R24>A24_ (Arg_24_ was mutated to Ala_24_), PemK_T47>D47_ (Thr_47_ was mutated to Asp_47_) and PemK_S48>W48_ (Ser_48_ was mutated to Trp_48_), compared with the wild-type PemK. **(E–H)** Electrostatic surface potential of PemK, PemK_R24>A24_, PemK_T47>D47_, and PemK_S48>W48_. The change in electrical potential energy at the point mutation locations were highlighted in dotted circles. **(I)** Growth curve of recombinant *E. coli* BL21 after induction of PemK and PemK_R24>A24_ expression. Data were assessed by ANOVA with Tukey’s *post-hoc* test. The asterisk (*) indicated significant level: *P < 0.05, **P < 0.01, ***P < 0.001, not significant (ns), *P* > 0.05.

The function of protein is often closely related to its structure and charge distribution (Gorham et al., 2011). To gain more detailed insights into the active site of PemK, mutations were predicted *in situ* at Arg_24_, Thr_47_, and Ser_48_ residues (Figure 4A). Interestingly, substitution of Arg_24_ by most amino acid strongly affected PemK function, resulting in a score ranging from 52 to 91 (Figure 4A and Supplementary Table 2). While substitution of Ser_48_ had weak or no effects with a score below 65 (Figure 4A). The impact of Thr_47_ substitution was generally less pronounced than that of Arg_24_, a score below 80 (Figure 4A). In PemK-mRNA complex, Arg_24_ bound to the phosphate group in U↓A through a hydrogen bond (Figure 3B). In *S. aureus* PemK and *B. subtilis* MazF, the amino acid residues, which bound to the phosphate group between U↓A via hydrogen bonding, were considered active sites involved in cleaving the tetrad U↓AUU sequence (Bukowski et al., 2013). Notably, Arg_24_ of PemK from *W. cibaria* corresponded well to Arg_25_ and Arg_26_ (Figure 2A), an important active residue in *S. aureus* PemK and *B. subtilis* MazF. So it might be a crucial active site for PemK to cleave the 3′,5′-phosphodiester bond of target mRNA.

The unfolding free energy (ΔGu) is an important indicator of protein thermostability, and the ΔΔGu (ΔΔG_u_ = ΔG_mut–u_—ΔG_wt–u_) can characterize the change in the thermostability caused by site-directed mutations (Verstraeten et al., 2015). The substituting *in situ* of Arg_24_, Thr_47_ and Ser_48_ decreased the thermostability of PemK, with substituting of Arg_24_ with Ala being more pronounced than the others, ΔΔGu = −0.91 (Figure 4B). In addition, the solvent accessible surface area (SASA) also characterized the instability caused by mutations (Figure 4C). A larger SASA value indicates a higher solvent-accessible surface area of protein, which is associated with lower stability. Three site-directed mutations *in situ* resulted in an increase in SASA of PemK, in which the ΔSASA_Arg24>Ala24_ (25.18) > ΔSASA_Ser48>Trp48_ (19.64) > ΔSASA_Thr47>Asp47_ (8.47) (Figure 4C).

The mutations may alter the charge distribution of protein’s active site or ligand-binding site, thereby impacting its affinity and specificity for the target molecules (Gorham et al., 2011). In *W. cibaria*, substituting Arg_24_ with Ala significantly altered the charge distribution on electrostatic potential surface of PemK dimers (Figures 4E, F), particularly at the like-gate interfaces where a transition occurred from positive charge to weak negative charge. Moreover, substituting Arg_24_ with Ala led to a more pronounced destabilization of PemK compared to Thr_47_ and Ser_48_ mutations, as indicated by ΔΔG_elec_ (ΔΔG_elec_ = ΔG_mut–elec_—ΔG_wt–elec_): ΔΔG_elec–Arg24>Ala24_ (0.11) > ΔΔG_elec–Ser48>Trp48_ (0.06) > ΔΔG_elec–Thr47>Asp47_ (0.04) (Figures 4D, G, H). Additionally, it was observed that PemK_Arg24>Ala24_ significantly reduced its cellar toxicity, without a significant inhibition on the recombinant *E. coli* BL21 (Figure 4I). These findings suggested that Arg_24_ was more likely to affect PemK’s ability to cleave U↓AUU sequence, thereby influencing cell regulation in response to adverse external environments.

### 3.4 PemK_Arg24>Ala24_ mitigates the impairment of respiratory chain

**FIGURE 5:**
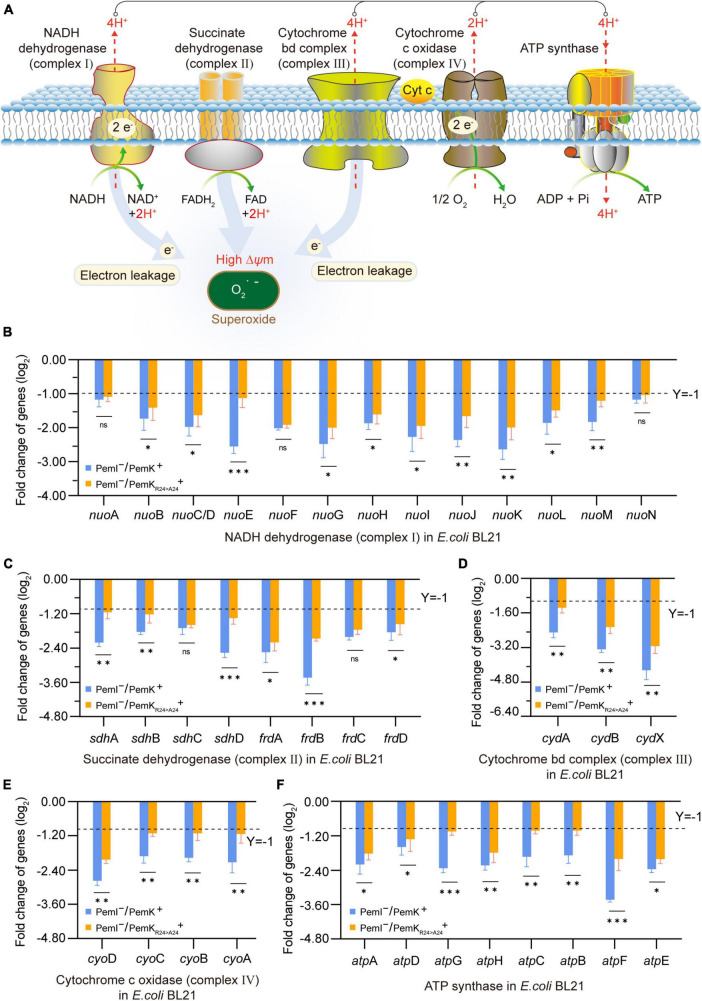
The effects on the respiratory chain before and after PemK mutation. **(A)** Schematic of respiratory chain. **(B–F)** Effects of PemK and PemK_R24>A24_ on gene transcription of complexes I–IV and ATP-synthase in recombinant *E. coli* BL21. Data were assessed by ANOVA with Tukey’s *post-hoc* test. The asterisk (*) indicated significant level: *P < 0.05, **P < 0.01, ***P < 0.001, not significant (ns), *P* > 0.05.

Persistence has been associated with reduced levels of cellular ATP (Verstraeten et al., 2015; Wilmaerts et al., 2019). The respiratory chain is the main process to generate cellular ATP, which is composed of a series of hydrogen transfer and electron transfer reactions mediated by complexes I to IV and ATP-synthase on cell plasma membrane (Figure 5A). In *W. cibaria*, PemK cleaved the target mRNAs of complexes I–IV and ATP-synthase, including 13 genes in complex I, 8 genes of complex II, 3 genes of complex III, 4 genes of complex IV, and 8 genes of ATP-synthase (Table 1 and Supplementary Table 3). In recombinant *E. coli* BL21, Ala substitution for Arg_24_ impaired PemK’s ability to cleave these target mRNAs, especially *nuo*E in complex I, *sdh*D and *frd*A in comolex II, *atp*G and *atp*F in ATP-synthase (Figures 5B–F), thus reducing the adverse effects of PemK on respiratory chain.

**TABLE 1:** PemK cleaving the target mRNAs of enzymes in respiratory chain of *W. cibaria*.

Metabolic enzymes	Gene	Number of cleavage sites	Metabolic enzymes	Gene	Number of cleavage sites
NADH dehydrogenase	*nuo*A	2	Succinate dehydrogenase	*frd*B	1
	*nuo*B	2		*frd*C	2
	*nuo*C/D	3		*frd*D	3
	*nuo*E	3	Cytochrome C reductase	*cyd*A	4
	*nuo*F	2		*cyd*B	4
	*nuo*G	8		*cyd*X	1
	*nuo*H	2	Cytochrome C oxidase	*cyo*D	1
	*nuo*I	1		*cyo*C	1
	*nuo*J	1		*cyo*B	6
	*nuo*K	1		*cyo*A	5
	*nuo*L	6	ATP-synthase	*atp*A	3
	*nuo*M	6		*atp*D	1
	*nuo*N	3		*atp*G	1
Succinate dehydrogenase	*sdh*C	2		*atp*H	2
	*sdh*D	1		*atp*C	1
	*sdh*A	3		*atp*B	4
	*sdh*B	3		*atp*A	1
	*frd*A	2		*atp*E	1

**FIGURE 6:**
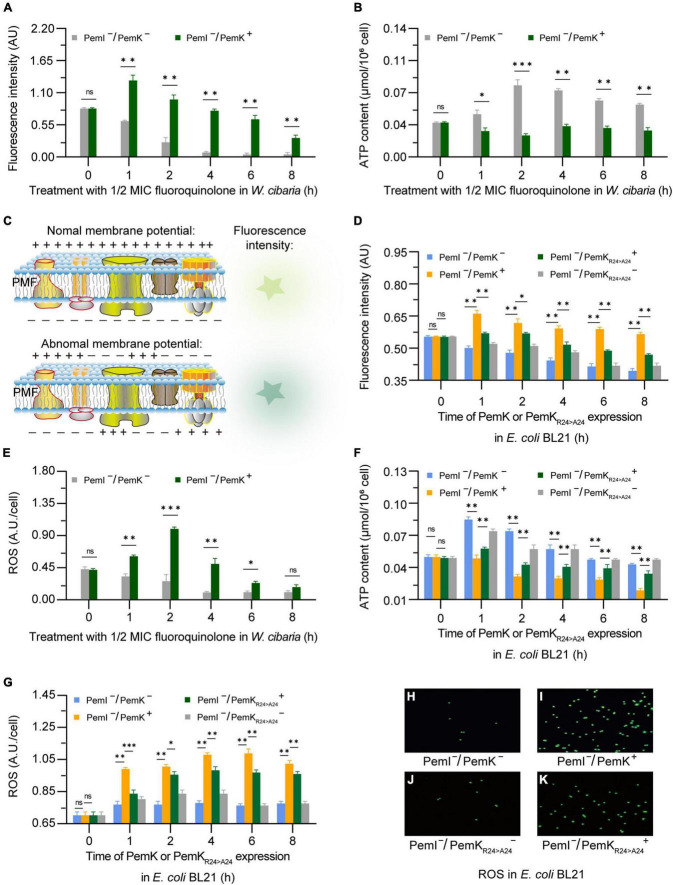
The alterations in membrane potential, ATP, and ROS caused by PemK and PemK_R24>A24_ in *W. cibaria* and recombinant *E. coli* BL21, respectively. **(A,B,E)** Membrane potential, ATP, and ROS of *W. cibaria* after treatment with 1/2 MIC ciprofloxacin. **(C)** Schematic of membrane potential. **(D,F,G)** Membrane potential, ATP, and ROS of recombinant *E. coli* BL21 after induction of PemK and PemK_R24>A24_ protein expression. **(H–K)** Fluorescence of intracellular ROS in recombinant *E. coli* BL21. Data were assessed by ANOVA with Tukey’s *post-hoc* test. The asterisk (*) indicated significant level: *P < 0.05, **P < 0.01, ***P < 0.001, not significant (ns), *P* > 0.05.

Cleavage of mRNA caused the mRNA to break before translation, thus it blocked the synthesis of complexes I–IV and ATPase, resulting in the failure of H^+^ and e^–^ transfer reactions in respiratory chain, which reduced the membrane potential and ATP production of *W. cibaria* (Figures 6A, B). Alterations in membrane potential caused membrane depolarization (Figure 6C), so the heightened fluorescence intensity corresponded to a more lower membrane potential (Figure 6A). In recombinant *E. coli* BL21, PemK significantly reduced the membrane potential and increased the membrane depolarization, while Ala substitution for Arg_24_ significantly alleviated the degree of membrane potential reduction and membrane depolarization, with an alleviation of 7.8–19.6% (Figure 6D). The membrane potential generated a proton motive force (PMF), which promotes the movement of H^+^ across cell membrane and ultimately produces ATP via ATP synthase. Therefore, the changes in membrane potential directly affected the production of ATP in *W. cibaria*, generally reducing the amount of ATP by 20–40% (Figure 6B). Additionally, blocked electron transport obstructed the reduction of O_2_, leading to the accumulation of excess ROS in *W. cibaria* to 1.50–2.30 times of normal levels (Figure 6E). Similarly, Ala substitution for Arg_24_ had also moderated the adverse effects on the ATP production and reduced ROS accumulation in recombinant *E. coli* BL21, with ATP content increased by 20.8–276.60% and ROS accumulation reduced by 7.8–15.6%, respectively (Figures 6F–K). These insights implied that Arg_24_ played an important role in regulation of respiratory chain by PemK.

### 3.5 PemK_Arg24>Ala24_ alleviates the adverse effects on NADH and FADH_2_ synthesis

**FIGURE 7:**
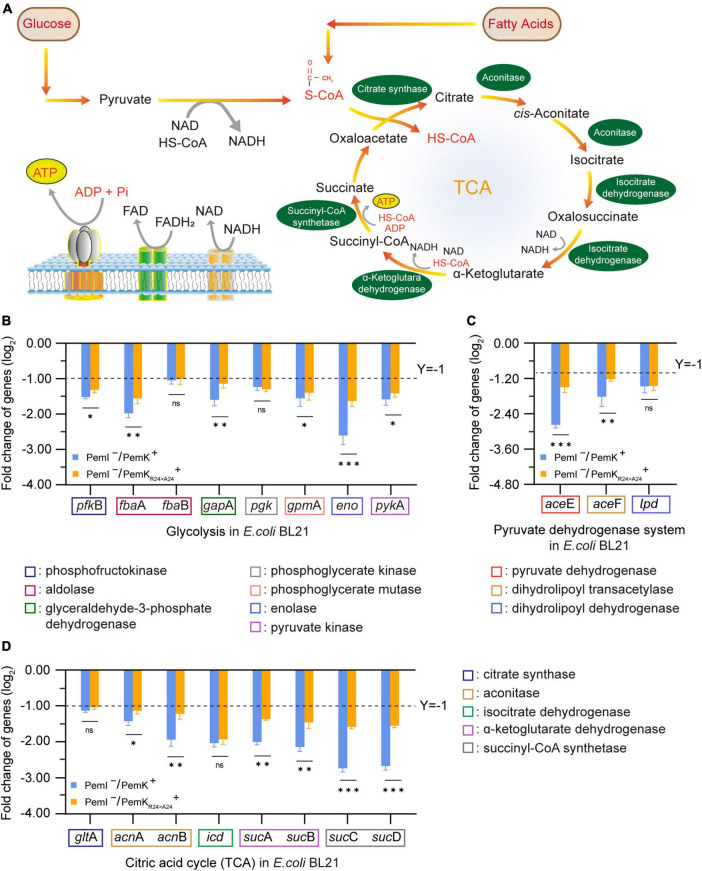
The effects on NADH and FADH_2_ synthesis before and after PemK mutation in recombinant *E. coli* BL21. **(A)** Schematic of glycolysis and TCA cycle. **(B)** Effects of PemK and PemK_R24>A24_ on the gene transcription of glycolysis. **(C)** Effects of PemK and PemK_R24>A24_ on the gene transcription of pyruvate dehydrogenase system. **(D)** Effects of PemK and PemK_R24>A24_ on the gene transcription of TCA cycle. Data were assessed by ANOVA with Tukey’s *post-hoc* test. The asterisk (*) indicated significant level: *P < 0.05, **P < 0.01, ***P < 0.001, not significant (ns), *P* > 0.05.

NADH and FADH_2_ are the important reducing agents to supply H^+^ for cells via the respiratory chain pathway, while glycolysis and TCA cycle serve as their primary sources. Glycolysis catalyzes a glucose into two pyruvates, accompanied by production of 2 NADH. This process involves ten enzyme-catalyzed reactions (Chandel, 2021), and any dysfunction of them can lead to disorders in NADH synthesis (Figure 7A). In glycolysis of *W. cibaria*, PemK only targeted the mRNA of one or two subunits of phosphofructokinase, aldolase, glyceraldehyde-3-phosphate dehydrogenase, phosphoglycerate kinase, phosphoglycerate mutase, enolase and pyruvate kinase (Table 2 and Supplementary Table 4).

**TABLE 2:** PemK cleaving the target mRNAs of enzymes in glycolysis of *W. cibaria*.

Metabolic enzymes	Gene	Number of cleavage sites
Phosphofructokinase	*pfk*B	2
Aldolase	*fba*A	1
	*fba*B	5
Glyceraldehyde-3-phosphate dehydrogenase	*gap*A	1
Phosphoglycerate kinase	*pgk*	1
Phosphoglycerate mutase	*gpm*A	2
Enolase	*eno*	1
Pyruvate kinase	*pykA*	1

Acetyl-CoA serves as the bridge between glycolysis and TCA cycle, generated by the pyruvate dehydrogenase system consisting of pyruvate dehydrogenase, dihydrolipoamide transacetylase, and dihydrolipoamide dehydrogenase. In *W. cibaria*, PemK also cleaved their mRNA (Table 3 and Supplementary Table 5). Acetyl-CoA enters TCA to undergo a series of enzymatic reactions into CO_2_ and H_2_O while producing 3 NADH and 1 FADH_2_. However, PemK only targeted the mRNA of one or two subunit of citrate synthase, aconitase, isocitrate dehydrogenase, α-ketoglutarate dehydrogenase and succinyl-CoA synthetase in *W. cibaria* (Table 4 and Supplementary Table 6). In recombinant *E. coli* BL21, PemK significantly impaired the mRNA of these target enzymes involved in glycolysis, pyruvate oxidative decarboxylation and TCA cycle by −1.05–2.98 log_2_ folds (Figures 7B–D). However, Ala substitution for Arg_24_ effectively alleviated these adverse impacts except for phosphoglycerate kinase, dihydrolipoamide dehydrogenase, citrate synthase and isocitrate dehydrogenase. Therefore, Arg_24_ was a crucial site for PemK to disrupt the NADH and FADH_2_ synthesis in *W. cibaria*.

**TABLE 3:** PemK cleaving the target mRNAs of pyruvate dehydrogenase system in *W. cibaria*.

Metabolic enzymes	Gene	Number of cleavage sites
Pyruvate dehydrogenase	*ace*E	6
Dihydrolipoamide transacetylase	*ace*F	2
Dihydrolipoamide dehydrogenase	*Ipd*	1

**TABLE 4:** PemK cleaving the target mRNAs of enzymes in TCA cycle of *W. cibaria*.

Metabolic enzymes	Gene	Number of cleavage sites
Citrate synthase	*glt*A	8
Aconitase	*acn*A	10
	*acn*B	5
Isocitrate dehydrogenase	*icd*	3
α-ketoglutarate dehydrogenase	*suc*A	2
	*suc*B	2
Succinyl-CoA synthetase	*suc*C	1
	*suc*D	1

### 3.6 PemK_Arg24>Ala24_ attenuates growth inhibition and persister frequency

**FIGURE 8:**
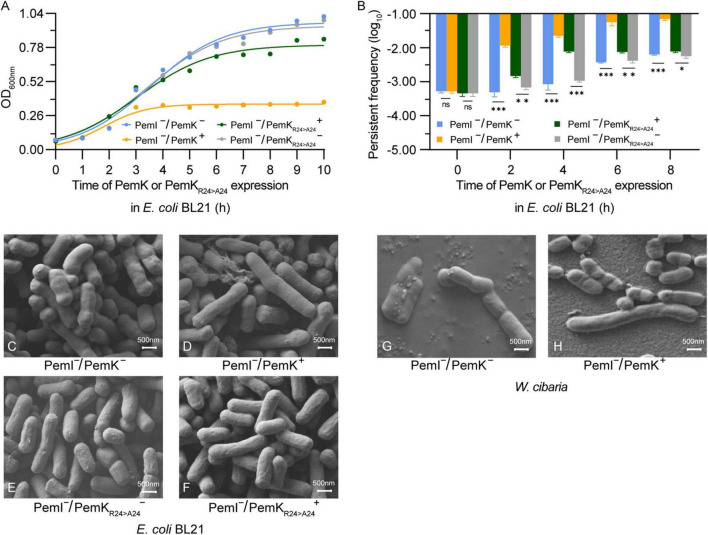
Persister frequency and cell morphology after induction of PemK and PemK_R24>A24_ expression. **(A)** Growth curve of recombinant *E. coli* BL21. **(B)** Persister frequency of recombinant *E. coli* BL21. **(C–F)** Cell morphology of *E. coli* BL21. **(G,H)** Cell morphology before and after induction of PemK expression in *W. cibaria*. Data were assessed by ANOVA with Tukey’s *post-hoc* test. The asterisk (*) indicated significant level: *P < 0.05, **P < 0.01, ***P < 0.001, not significant (ns), *P* > 0.05.

Induction of persistence appears to be the primary physiological function of TAs in bacteria, and stimulating the expression of type II toxins can significantly enhance the persister frequency (Maisonneuve and Gerdes, 2014; Merfa et al., 2016). In recombinant *E. coli* BL21, induction of PemK resulted in significant growth inhibition, with OD_600nm_ maintained at 0.29–0.31 (Figure 8A). Although the persister frequency also increased from −3.5 log_10_ to −2.58 log_10_ during growth of *E. coli* BL21 with PemK^–^/PemI^–^, PemK expression dramatically elevated the persister frequency of *E. coli* BL21 with PemI^–^/PemK^+^ up to −1.82 log_10_ to −0.24 log_10_ (Figure 8B). Substitution of Arg_24_ with Ala_24_ significantly weakened the growth inhibition and induced persister frequency of *E. coli* BL21 with PemI^–^/PemK_Arg24>Ala24_^+^, decreasing it to −2.82 to −2.24 log_10_ (Figure 8B).

II-type toxin expression does not necessarily lead to changes in morphology of persister cells, but when it does, it usually involves abnormal elongation, filamentation, and even a transition from spiral to round coccoid (Cho et al., 2017; El Mortaji et al., 2020; Yu et al., 2023). In recombinant *E. coli* BL21, PemK expression significantly prolonged the morphology of some cells, increasing from 0.75–1.5 μm to 1.5–2.5 μm. This change was also observed in *W. cibaria*, but with more pronounced effects from 0.75–1.25 to 0.75–3.5 μm. However, the mutation PemK_Arg24>Ala24_ did not induce significant alterations of *E. coli* BL21 (Figures 8C–H). It was generally believed that ectopic expression of a single toxin gene resulted in a phenotype similar to naturally occurring persister bacteria (Merfa et al., 2016; Page and Peti, 2016). Therefore, PemK have prolonged the persister cell of *W. cibaria* through the same mechanism as *E. coli* BL21.

## 4 Discussion

The mechanism underlying TA-mediated persistence remains incompletely understood, although it is generally believed that the direct cause is the shutdown or disruption of major cellular processes induced by TA toxins (Verstraeten et al., 2015; Wilmaerts et al., 2019). However, experimental confirmation of this understanding has been scarce. Our presented results revealed the molecular mechanism through which PemK induced persistence of *W. cibaria* by disrupting crucial cellular processes such as hydrogen transfer, electron transfer, NADH and FADH_2_ synthesis (Figures 3, 5, 7). This disruption occurred via mRNA fragmentation of targeting metabolic enzymes involved in glycolysis, TCA cycle and respiratory chain pathways, ultimately leading to reduced ATP levels and membrane depolarization (Figures 6A–C). These findings provided valuable insights into how *W. cibaria* evaded ciprofloxacin stress, that was, when faced with ciprofloxacin stress, it entered the persistence, conversely, once the stress was alleviated, the persistence was awakened.

Bacterial persistence is a programmed phenotypic transformation with a genetic basis (Gerdes and Maisonneuve, 2012; Wilmaerts et al., 2019). Currently, it is widely accepted that TAs play a crucial role in bacterial persistence (Page and Peti, 2016). Our findings further supported this consensus, as PemKI’s mRNA was one of the most up-regulated transcripts during the persistence of *W. cibaria* under ciprofloxacin stress (Figure 1C). Additionally, ectopic expression of PemK significantly increased the persistence frequency of recombinant *E. coli* BL21 (Figure 8B). In *W. cibaria*, the PemKI module consisted of PemK and its cognate antitoxin PemI (Figures 1A, B). Although PemK shared conserved residues and similar structure with homologs, its gate crevice and charge distribution differed from those homologs due to amino acid variations. Notably, the gate crevice of PemK closely resembled that of *S. aureus*, but differed significantly from *E. coli*, *B. subtilis* and *B. anthracis* (Figure 2). These unique characteristics determined its binding specificity for mRNA and physiological functions.

The significant function of PemK and its homologues is to catalyze the cleavage of bacterial mRNA with specific sequences (Bukowski et al., 2013; Kim et al., 2022), and their catalytic activities are closely related to the type and location of amino acid residues involved in mRNA binding and cleavage. In *E. coli*, MazF specifically cleaves the P-O5′ phosphoester bond between G↓ACA or G/UA↓CA of mRNA through transphosphorylation, with Arg_29_ and Thr_52_ likely serving as catalytic residues (Zorzini et al., 2016). MazF from *B. subtilis* cleaves mRNAs with U↓ACAU, where Arg_25_ and Thr_48_ are crucial for its activity, while Gln_50_ and Arg_71_ have minimal effect on activity (Simanshu et al., 2013). For *S. aureus*, Glu_20_, Arg_25_, Th_r48_, and Arg_84_ are key residues involved in binding and cleavage of mRNA with U↓AUU. Among them, Arg_25_ and Thr_48_ may be responsible for mRNA cleavage, while Arg_84_ maintains the “closed form” conformation of PemK dimer despite not directly interacting with mRNA. Conserved Arg and Thr residues located in β2 and β3 sheet, respectively, are generally considered as the active residues for catalysis in PemK homologues, where Arg acts as both a general acid/base catalyst, while Thr stabilizes transition states during reactions. In *W. cibaria*, Arg_24_ bound the phosphate group between U↓A, its mutation significantly affected the conformation and charge distribution of its gate crevice, as well as the thermostability, SASA and instability of PemK, thus significantly reducing the catalytic activity of PemK (Figures 3B, 4–8). Interestingly, substitution at Thr_48_ with any amino acid did not obviously affect any properties described above for PemK (Figure 4A), most likely due to fact that it bound the adenine between U↓A rather than phosphate group in target mRNA sequence (Figure 3B). Therefore, the active residues of PemK from *W. cibaria* differed significantly from those found in its homologs, where only Arg_24_ was the crucial active residue.

A decrease in intracellular ATP and/or low PMF across the membrane are characteristic indicators of persister formation resulting from TA-toxin attacking on conservative metabolic processes (Yu et al., 2023). Various small toxins, such as those in the Hok family, abrogate the PMF and inhibit ATP synthesis by depolarizing bacterial membranes (Harms et al., 2018). However, there is currently limited knowledge regarding the detailed mechanism through which TA-toxins target ATP and PMF. In *W. cibaria*, ATP synthesis primarily occurs through substrate level phosphorylation in glycolysis and TCA cycle, as well as oxidative phosphorylation in the respiratory chain. The former serves as an auxiliary pathway for energy acquisition, with only 3 ATP production. However, PemK inhibited their ATP synthesis, more importantly, it reduced the supply of NADH + H^+^ and FADH_2_ for oxidative phosphorylation by cleaving mRNA of enzymes in metabolic framework (Figures 5–7). The oxidative phosphorylation is the primary pathway for ATP production, generating 10 ATP molecules along with the efflux of 10 H^+^ through complexes I, III, and IV, and the influx of 4 H^+^ through ATP-synthase. Consequently, in *W. cibaria*, PemK cleaved target mRNAs encoding complexes I, III and IV to result in impaired H^+^ efflux from inner-membrane, subsequently reduced the membrane potential and abolished PMF (Figures 5, 6A, C). Simultaneously, PemK targeted complexes I–IV and ATP synthase to block the electron transfer and H^+^ influx which ultimately disrupts ATP synthesis (Figures 6B, F). In bacteria, disruption of ATP synthesis appears to be a promising mechanism for TA-toxins as most antibiotics corrode active targets by energy-dependent processes. Therefore, the disruption of ATP synthesis leading to cell depolarization was likely to be the primary physiological function of PemK inducing *W. cibaria* persistence to evade ciprofloxacin stress.

Recently, it has been documented that ATP content was the decisive factor in whether exponentially growing cells transition into persister cells. However, this correlation was not observed in *S. aureus* (Kamphuis et al., 2006; Somerville and Proctor, 2009). Under ciprofloxacin stress, PemK significantly augmented the persister frequency of *W. cibaria*, from 0.03% up to 0.6% (Figure 1C), achieved through disruption of ATP synthesis pathway and depolarization of cell membrane, which has been further validated in recombinant *E. coli* BL21 (Figures 6A–D, F). Moreover, when Ala replaced Arg_24_, PemK_Arg24>Ala24_ considerably weakened its cytotoxicity to ATP synthesis pathway and membrane potential of recombinant *E. coli* BL21, resulting in a decrease in persister frequency from −1.82 to −1.24 log_10_ to −2.82 to −2.24 log_10_ (Figures 6D, 8A). These ectopic results further corroborated the physiological mechanism underlying PemK’s involvement in the persister formation of *W. cibaria*, while also highlighted the significance of Arg_24_ for maintaining PemK’s activity.

The significance of TA system in bacterial persistence renders it a natural target for the discovery of innovative strategies that can either trigger the persistence or awaken the persister cell. Our study provided some crucial structural and functional insights into PemK of *W. cibaria*, serving as a fundamental basis for uncovering this novel approach. However, the pivotal question remains whether interference with mRNA and physiological function by ciprofloxacin-induced PemK characterizes universal regulatory on *W. cibaria* under diverse stresses, which currently lacks clarity and necessitates further investigation.

## Data availability statement

The datasets presented in this study can be found in online repositories. The names of the repository/repositories and accession number(s) can be found in this article/Supplementary material.

## Author contributions

H-YZ: Data curation, Formal analysis, Investigation, Methodology, Software, Validation, Visualization, Writing – original draft. W-LX: Conceptualization, Funding acquisition, Resources, Supervision, Writing – review & editing. TC: Data curation, Formal analysis, Writing – original draft. MZ: Data curation, Investigation, Methodology, Writing – original draft. H-YW: Formal analysis, Validation, Writing – original draft.

## Footnotes

1
http://www.jalview.org

2
https://rostlab.org/services/snap/

3
https://www.ics.uci.edu/∼baldig/mutation.html

4
http://www.ccp4.ac.uk/
