# Supplementary material for: PemK’s Arg24 is a crucial residue for PemIK toxin–antitoxin system to induce the persistence of Weissella cibaria against ciprofloxacin stress
Source: Front Microbiol. 2024 May 14;15:1402319. doi: 10.3389/fmicb.2024.1402319 (PMC11130411; doi:10.3389/fmicb.2024.1402319)
Supplement: Supplementary file 1 [file Data_Sheet_1.pdf]

Supplementary Material

PemK's Arg24 is a crucial residue for PemIK toxin–antitoxin system to promote the persistence of *Weissella cibaria*

Hao-Yu Zhu [1, ✦], Wen-Liang Xiang [1, 2, *, ✦], Ting Cai [1, 2], Yi-Min Cai [3], Min Zhang [2], Han-Yang Wang [2], Jie Tang [1, 2], Qing Zhang [1, 2*]

Correspondence: Wen-Liang Xiang
Email: biounicom@mail.xhu.edu.cn

FIG S1 Type II TA systems modules of *W. cibaria* CGMCC 1.19376.
TABLE S1 Primer sequences of PemK and PemI

TABLE S2 The predicting impact scores on the function of PemK protein after mutation

TABLE S3 The mRNA of metabolic enzymes involved in oxidative phosphorylation

TABLE S4 The mRNA of metabolic enzymes involved in glycolysis

TABLE S5 The mRNA of metabolic enzymes involved in pyruvate dehydrogenase system

TABLE S6 The mRNA of metabolic enzymes involved in citric acid cycle (TCA cycle)

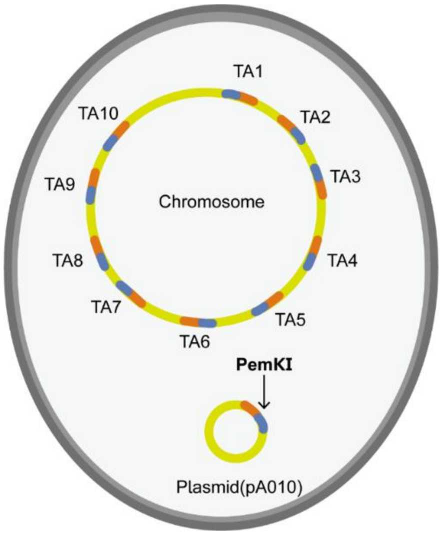

FIG S1 Type II TA systems modules of *W. cibaria* CGMCC 1.19376.

TABLE S1 Primer sequences of PemK and PemI

Primer Name	Primer sequence (5' - 3')
PemK-F	GTAAGCCAAGGTGATATATTTTATGTTAACTTTAATCC
PemK-R	CTATTTGTCATCAATACTAAACAATAATTTGTACAAAGTG
PemI-F	ATGACCTCCAGTAAGAATATTTGCATTGT
PemI-R	TTACCATTGAAGTTCATTACCTTCTGACTTTCC

TABLE S2 The predicting impact scores on the function of PemK protein after mutation

Sites of mutation / Species of residue	Asn22	Arg24	Thr47	Ser48
Ala	-1	91	48	16
Arg	29	-99	63	23
Asn	-99	82	65	-14
Asp	0	69	80	55
Cys	11	55	49	40
Gln	9	65	62	6
Glu	20	84	71	46
Gly	-54	84	66	29
His	-26	52	65	-7
Ile	29	73	63	56
Leu	27	72	65	50
Lys	0	65	73	29
Met	22	68	62	41
Phe	1	79	68	58
Pro	37	87	73	50
Ser	-32	78	-45	-99
Thr	-11	77	-99	-25
Trp	40	82	75	65
Tyr	28	78	69	56
Val	23	73	65	35

TABLE S3 The mRNA of metabolic enzymes involved in oxidative phosphorylation

Oxidative phosphorylation		
Enzyme	Gene	Sequence (mRNA)
NADH dehydrogenase (complexe I)	*nuo*A	AUGAGUAUGUCAACAUCCACUGAAGUCAUCGCUCAUCACU<br>GGGCAUUCGCUAUCUUUCUUAUCGUUGCCAUUGGCCUGU<br>GUUGCCUGAUGCUGGUAGGCGGUUGGUUUUUAGGCGGU<br>CGCGCACGCGCGAGGUCGAAAAACGUGCCGUUUGAAUCC<br>GGUAUCGACUCGGUCGGCUCCGCCCGCUUACGCCUGUCC<br>GCCAAGUUUUAUCUGGUGGCCAUGUUCUUCGUUAUCUUC<br>GACGUUGAAGCGCUGUAUCUGUUCGCAUGGUCAACCUCU<br>AUCCGCGAAAGCGGCUGGGUAGGCUUUGUGGAAGCUGCA<br>AUUUU**UAUU**UUUGUGUUACUGGCAGGUCUGGUUUAUCUG<br>GUGCG**UAUU**GGCGCGCUGGACUGGACGCCCGCGCGUUC<br>ACGCCGCGAGCGUAUGAACCCGGAAACGAACAGUAUCGC<br>UAAUCGUCAACGCUAA
	*nuo*B	AUGGAUUAUACGCUCACCCGCAUAGAUCCCAACGGUGAG<br>AACGACCGUUACCCCCUGCAAAAGCAGGAGAUCGUAACCG<br>ACCCUCUGGAGCAAGAAGUUAACAAAAACGUGUUUAUGGG<br>CAAGCUCAAUGACAUGGUUAACUGGGGUCGUAAAAACUCA<br>AUUUGGCCGUAUAACUUCGGUCUUUCCUGCUGUUACGUU<br>GAGAUGGUGACUUCGUUUACCGCGGUGCAUGACGUGGCG<br>CGUUUUGGCGCAGAAG**UAUU**GCGUGCUUCGCCGCGUCAG<br>GCUGACCUGAUGGUGGUUGCAGGAACCUGCUUUACCAAA<br>AUGGCACCGGUCAUUCAGCGUCUGUAUGACCAGAUGCUG<br>GAACCAAAAUGGGUUAUCUCAAUGGGUGCCUGUGCCAAC<br>UCUGGUGGUAUGUACGA**UAUUUAUU**CCGUUGUGCAGGGC<br>GUCGAUAAAUUCAUCCCGGUUGAUGUGUAUAUCCCGGGC<br>UGCCCGCCGCGUCCUGAAGCGUACAUGCAGGCACUGAUG<br>CUGUUGCAGGAAUCUAUCGGCAAAGAACGUCGUCCGCUC<br>UCCUGGGUGGUUGGCGAUCAGGGCGUUUAUCGCGCCAAU<br>AUGCAAUCAGAGCGCGAACGCAAGCGCGGUGAACGCAUU<br>GCCGUAACUAACCUGCGUACACCUGACGAGAUUUAA
	*nuo*C/D	AUGGUGAACAAUAUGACCGACUUAACCGCGCAAGAACCCG<br>CCUGGCAGACCCGCGAUCAUCUUGAUGAUCCGGUGAUUG<br>GCGAACUGCGCAACCGUUUUGGGCCGGAUGCCUUUACUG<br>UUCAGGCGACUCGCACCGGGGUUCCCGUUGUGUGGAUCA<br>AGCGUGAACAAUUACUGGAAGUUGGCGAUUUCUUAAAGAA<br>ACUGCCGAAACCUUACGUCAUGCUGUUUGACUUACACGG<br>CAUGGACGAACGUCUGCGCACACACCGCGAAGGGUUACC<br>UGCCGCGGAUUUUUCCGUUUUCUACCAUCUGAUUUCUAU<br>CGAUCGUAACCGCGACAUCAUGCUGAAGGUGGCGCUGGC<br>AGAAAACGACCUGCACGUACCGACCUUCACCAAACUGUUC<br>CCGAACGCUAACUGGUAUGAGCGUGAAACCUGGGAUCUG<br>UUUGGCAUUACUUUCGACGGUCACCCGAACCUGCGACGC<br>AUCAUGAUGCCGCAAACCUGGAAAGGUCACCCGCUGCGU<br>AAAGAUUAUCCGGCGCGCGCUACCGAAUUCUCGCCGUUU<br>GAGCUGACCAAAGCCAAACAGGAUCUGGAGAUGGAAGCC<br>CUGACCUUCAAACCGGAAGAGUGGGGGAUGAAGCGCGGC<br>ACCGAAAACGAGGACUUCAUGUUCCUCAACCUCGGUCCG<br>AACCACCCGUCGGCGCACGGGGCUUUCCGUAUCGUUUUG<br>CAACUCGAUGGCGAAGAGAUUGUCGACUGCGUACCAGAC<br>AUCGGUUACCACCACCGUGGUGCGGAGAAAAUGGGCGAA<br>CGCCAGUCCUGGCACAGCUACAUUCCGUAUACUGACCGU<br>AUCGAAUACCUCGGCGGCUGCGUUAACGAAAUGCCUUAC

		GUGCUGGCGGUAGAGAAACUGGCCGGGAUCACCGUGCCG<br>GAUCGCGUUAACGUCAUUCGCGUUAUGCUCUCCGAACUG<br>UUCCGCAUCAACAGUCACCUGCUGUAUAUCUCGACCUU**U**<br>**AUU**CAGGACGUCGGCGCAAUGACGCCAGUGUUCUUCGCC<br>UUUACCGAUCGUCAGAAAAUUUACGAUCUGGUGGAAGCG<br>AUCACGGGUUUCCGUAUGCACCCGGCGUGGUUCCG**UAUU**<br>GGCGGCGUAGCGCACGACCUGCCGCGCGGCUGGGAUCG<br>CCUGCUGCGUGAGUUCCUCGACUGGAUGCCGAAACGUCU<br>GGCGUCUUACGAGAAAGCGGCGCUGCAAAACACCAUUCU<br>GAAAGGUCGUUCCCAGGGCGUUGCCGCCUAUGGCGCGAA<br>AGAGGCGCUGGAGUGGGGCACCACUGGCGCGGGCCUGC<br>GUGCUACCGGGAUCGACUUCGACGUGCGUAAGGCGCGUC<br>CU**UAUU**CUGGCUAUGAAAACUUCGACUUUGAAAUCCCGG<br>UGGGUGGUGGCGUUUCUGACUGCUACACCCGCGUAAUGC<br>UUAAAGUGGAAGAGCUGCGCCAGAGUCUGCGCAUUCUUG<br>AGCAGUGCCUCAACAACAUGCCGGAAGGCCCGUUCAAAG<br>CGGAUCACCCGCUGACCACGCCGCCGCCGAAAGAGCGCA<br>CGCUGCAACAUAUCGAAACCCUGAUCACCCACUUCCUGCA<br>AGUGUCGUGGGGGCCGGUGAUGCCUGCCAAUGAAUCUUU<br>CCAGAUGAUUGAGGCGACCAAGGGGAUCAACAGUUACUA<br>CCUGACCAGCGACGGUAGCACCAUGAGUUAUCGCACUCG<br>UAUCCGCACGCCGAGUUAUGCGCAUUUGCAGCAAAUUCC<br>GGCGGCGAUCCGCGGCAGCCUGGUGUCUGACCUGAUUG<br>UUUAUCUGGGCAGUAUCGAUUUUGUUAUGUCAGAUGUGG<br>ACCGCUAA
	*nuo*E	AUGCACGAGAAUCAACAACCACAAACCGAGGCUUUUGAGC<br>UGAGUGCGGCAGAGCGUGAAGCGAUUGAGCACGAGAUGC<br>ACCACUACGAAGACCCGCGUGCGGCGUCCAUUGAAGCGC<br>UGAAAAUCGUUCAGAAGCAGCGUGGCUGGGUGCCGGAUG<br>GUGCGAUCCACGCGAUCGCCGAUGUGCUGGG**UAUU**CCG<br>GCAAGCGACGUCGAAGGUGUGGCAACGUUCUACAGUCAG<br>AUCUUCCGCCAGCCGGUUGGUCGCCAUGUGAUCCGU**UAU**<br>**U**GCGACAGCGUGGUCUGUCAUAUCAACGGGUAUCAGGG**U**<br>**AUU**CAGGCGGCACUCGAGAAAAAGCUGAACAUCAAACCAG<br>GGCAAACGACAUUUGAUGGCCGCUUUACGCUGCUGCCAA<br>CUUGCUGCCUGGGGAACUGUGAUAAAGGGCCAAACAUGA<br>UGAUCGAUGAGGACACUCACGCGCAUCUGACCCCGGAAG<br>CGAUCCCUGAACUGCUGGAGCGGUAUAAAUGA
	*nuo*F	AUGAAAAACAUUAUCCGUACUCCCGAAACGCAUCCGCUGA<br>CCUGGCGUCUGCGCGAUGACAAACAGCCAGUGUGGCUGG<br>ACGAAUAUCGCAGCAAAAACGGUUACGAAGGCGCGCGUAA<br>GGCGCUGACCGGGCUGUCUCCGGACGAAAUCGUUAAUCA<br>GGUAAAAGACGCUGGUCUGAAAGGGCGCGGUGGCGCGG<br>GCUUUUCUACCGGCCUGAAAUGGAGCCUGAUGCCGAAAG<br>ACGAAUCCAUGAACAUCCGUUACCUGCUGUGUAACGCCG<br>AUGAAAUGGAGCCAGGCACCUAUAAAGACCGCCUGCUGA<br>UGGAGCAACUGCCGCACCUGCUGGUGGAAGGCAUGCUCA<br>UCUCCGCGUUUGCGCUGAAAGCUUACCGUGGCUAUAUCU<br>UCCUGCGCGGCGAAUAUAUCGAAGCGGCAGUGAAUCUAC<br>GCCGUGCCAUUGCCGAAGCCACCGAAGCAGGUCUGCUUG<br>GCAAAAA**UAUU**AUGGGAACAGGUUUUGACUUCGAACUGU<br>UCGUCCAUACCGGGGCAGGGCGCUACAUCUGCGGCGAAG<br>AAACAGCGUUAAUCAACUCCCUGGAAGGACGUCGUGCUAA<br>CCCACGCUCGAAGCCUCCAUUCCCGGCGACCUCCGGCGC

		AUGGGGUAAACCGACCUGUGUCAACAACGUCGAAACCCU<br>GUGUAACGUUCCGGCGAUCCUCGCUAACGGCGUGGAGUG<br>GUAUCAGAACAUCUCGAAAAGUAAAGAUGCUGGCACCAAG<br>CUGAUGGGCUUCUCCGGUCGGGUGAAAAAUCCGGGCCUG<br>UGGGAACUGCCGUUCGGCACCACCGCACGCGAGAUCCUC<br>GAAGAUUACGCCGGUGGUAUGCGUGACGGUCUGAAAUUC<br>AAAGCCUGGCAGCCAGGCGGCGCGGGGACUGACUUCCUG<br>ACCGAAGCGCACCUUGACCUGCCGAUGGAAUUCGAAAGU<br>AUCGGUAAAGCGGGCAGCCGUCUGGGUACGGCGCUGGC<br>GAUGGCGGUUGACCAUGAGAUCAACAUGGUGUCGCUGGU<br>GCGUAACCUGGAAGAGUUUUUCGCCCGUGAGUCCUGCGG<br>CUGGUGUACGCCGUGCCGCGACGGUCUGCCGUGGAGCG<br>UGAAAAUUCUGCGUGCGCUGGAGCGUGGUGAAGGUCAGC<br>CAGGCGAUAUCGAAACACUUGAGCAACUGUGUCGAUUCU<br>UAGGCCCGGGUAAAACUUUCUGUGCCCACGCACCUGGUG<br>CAGUGGAGCCGUUACAGAGCGCCAUCAAA**UAUU**UCCGCG<br>AAGAAUUUGAGGCGGGAAUCAAACAGCCGUUCAGCAAUAC<br>CCAUUUGAUUAAUGGGAUUCAGCCGAACCUGCUGAAAGA<br>GCGCUGGUAA
	*nuo*G	AUGGCUACAAUUCAUGUAGACGGCAAAGAAUACGAGGUCA<br>ACGGAGCGGACAACCUGCUGGAAGCUUGUCUGUCUCUGG<br>GCCUUGA**UAUU**CCUUACUUUUGCUGGCAUCCGGCGCUGG<br>GAAGCGUCGGUGCUUGCCGCCAGUGUGCGGUGAAGCAAU<br>ACCAAAACGCGGAAGACACGCGUGGUCGCCUGGUGAUGU<br>CCUGUAUGACACCGGCUUCCGAUGGCACCUU**UAUU**UCCA<br>UUGACGACGAAGAAGCGAAACAGUUCCGUGAAAGUGUGG<br>UCGAGUGGUUAAUGACCAACCACCCGCACGACUGUCCGG<br>UAUGUGAAGAAGGCGGUAACUGCCAUCUUCAGGAUAUGA<br>CCGUGAUGACCGGACACAGCUUCCGUCGCUACCGUUUCA<br>CCAAACGUACCCACCGUAAUCAGGAUUUGGGACCGUUCA<br>UCUCUCACGAAAUGAACCGCUGCAUCGCCUGCUACCGCU<br>GCGUGCGUUACUACAAAGAUUACGCUGGCGGUACAGAUC<br>UGGGCGUUUACGGUGCGCACGACAACGUCUACUUCGGUC<br>GCCCGGAAGACGGCACGCUGGAAAGCGAAUUUUCCGGUA<br>ACCUGGUCGAAAUUUGCCCGACCGGCG**UAUU**UACCGACA<br>AAACGCACUCCGAGCGUUACAACCGUAAAUGGGAUAUGCA<br>GUUUGCGCCGAGCAUCUGCCAGCAAUGUUCCAUCGGCUG<br>UAACAUCAGCCCCGGCGAGCGUUAUGGCGAACUGCGUCG<br>UAUCGAAAACCGUUACAACGGUACGGUAAACCACUACUUC<br>CUCUGCGACCGUGGUCGUUUCGGUUACGGUUACGUCAAC<br>CUGAAAGAUCGUCCGCGUCAGCCAGUACAGCGUCGUGGC<br>GAUGAUUUCAUUACCCUCAACGCCGAACAGGCAAUGCAG<br>GGCGCAGCAGA**UAUU**CUGCGUCAGUCGAAGAAAGUGAUC<br>GGCAUUGGUUCUCCGCGCGCCAGCGUGGAAAGCAACUUU<br>GCGCUGCGUGAGCUGGUGGGCGAAGAAAACUUCUACACC<br>GGUAUCGCUCACGGUGAGCAGGAACGUCUGCAACUGGCG<br>CUGAAAGUACUGCGUGAAGGCGGCAUUUAUACUCCUGCU<br>CUGCGCGAAAUCGAAUCUUACGAUGCGGUACUGGUGCUG<br>GGCGAAGACGUUACCCAGACCGGCGCGCGCGUCGCGCUG<br>GCAGUGCGUCAGGCGGUGAAAGGUAAAGCGCGCGAAAUG<br>GCUGCAGCACAGAAAGUGGCUGACUGGCAGAUUGCGGCA<br>AUCCUCAACAUCGGUCAACGUGCGAAGCAUCCGCUGUUU<br>GUUACCAACGUUGAUGACACUCGUCUGGAUGAUAUCGCG<br>GCGUGGACUUACCGCGCACCGGUUGAAGAUCAGGCGCGU<br>UUAGGUUUUGCCAUCGCCCAUGCGCUGGAUAACUCUGCA

		CCAGCGGUUGACGGUAUCGAACCUGAGCUGCAAAGCAAA<br>AUCGACGUCAUCGUGCAGGCACUGGCUGGUGCGAAGAAA<br>CCGUUGAUUAUCUCCGGGACGAACGCCGGUAGCUUAGAG<br>GUGAUUCAGGCGGCGGCUAACGUCGCGAAAGCCCUGAAA<br>GGUCGCGGCGCUGACGUUGGUAUCACCAUGAUUGCUCGC<br>UCUGUAAACAGCAUGGGGCUGGGCAUUAUGGGUGGCGGU<br>UCGCUUGAAGAAGCGUUGACCGAACUGGAAACCGGACGC<br>GCCGACGCGGUGGUGGUGUUGGAAAACGAUCUGCAUCGU<br>CACGCUUCUGCUACCCGCGUGAAUGCUGCGCUGGCUAAA<br>GCACCGCUGGUGAUGGUGGUUGAUCAUCAACGCACAGCG<br>AUUAUGGAAAACGCCCAUCUGGUACUUUCUGCUGCCAGC<br>UUUGCUGAAAGCGACGGUACGGUGAUCAACAACGAAGGC<br>CGCGCCCAACGUUUCUUCCAGGUUUACGAUCCUGCU**UAU**<br>**U**ACGACAGCAAAACUGUCAUGCUGGAAAGCUGGCGCUGG<br>UUACACUCGCUGCACAGCACCCUGCUGAGCCGUGAAGUG<br>GACUGGACGCAGCUCGACCAUGUGAUUGACGCUGUUGUG<br>GCGAAAAUCCCGGAACUGGCAGGUAUCAAAGAUGCUGCG<br>CCGGAUGCGACAUUCCG**UAUU**CGUGGGCAGAAACUGGCC<br>CGUGAACCGCACCGUUACAGCGGUCGUACCGCCAUGCGC<br>GCCAAUAUCAGCGUUCAUGAGCCGCGUCAGCCGCAGGA**U**<br>**AUU**GACACCAUGUUCACCUUCUCGAUGGAAGGUAACAACC<br>AGCCGACUGCGCACCGUUCGCAAGUGCCGUUUGCCUGGG<br>CGCCGGGCUGGAACUCCCCGCAGGCGUGGAACAAAUUCC<br>AGGACGAAGUGGGCGGCAAACUGCGCUUUGGCGAUCCGG<br>GCGUGCGUCUGUUUGAAACCAGCGAAAAUGGUCUGGAUU<br>ACUUCACCAGCGUACCGGCACGCUUCCAGCCGCAGGACG<br>GGAAAUGGCGUAUCGCGCCG**UAUU**ACCACCUGUUUGGCA<br>GCGAUGAAUUGUCACAGCGUGCUCCGGUCUUCCAGAGCC<br>GUAUGCCGCAGCCGUACAUCAAACUCAACCCAGCGGAUG<br>CCGCGAAGUUGGGUGUGAACGCAGGUACACGCGUCUCCU<br>UUAGUUACGAUGGCAACACGGUCACGCUGCCGGUUGAAA<br>UCGCCGAAGGACUGACGGCAGGGCAGGUGGGCUUGCCG<br>AUGGGUAUGUCCGGCAUUGCUCCGGUGCUGGCUGGCGC<br>GCAUCUUGAGGAUCUCAAGGAGGCACAACAAUGA
	*nuo*H	AUGAGUUGGAUAUCACCGGAACUGAUUGAGAUCCUGCUG<br>ACCAUCCUCAAAGCGGUGGUGAUCCUGCUGGUGGUUGUC<br>ACCUGCGGGGCAUUCAUGAGCUUUGGCGAACGUCGCCUG<br>CUGGGUCUGUUCCAGAACCGUUACGGACCUAACCGUGUU<br>GGCUGGGGCGGUUCGCUCCAGCUGGUUGCGGACAUGAU<br>CAAAAUGUUCUUUAAAGAAGACUGGAUCCCGAAAUUCUCG<br>GAUCGCGUCAUCUUUACCCUGGCACCGAUGAUUGCCUUU<br>ACCUCGCUGCUGCUGGCCUUUGCGAUUGUGCCAGUCAGU<br>CCGGGUUGGGUGGUUGCCGACCUGAACAUCGGGAUUUU<br>GUUCUUCCUGAUGAUGGCAGGUCUGGCGGUUUACGCGG<br>UGCUGUUUGCGGGCUGGUCAAGUAACAACAAAUACUCGU<br>UGCUGGGUGCGAUGCGUGCUUCUGCGCAGACCCUGAGC<br>UACGAAGUGUUCCUCGGGCUUUCCUUGAUGGGCGUGGU<br>GGCGCAGGCCGGUUCAUUCAACAUGACCGACAUCGUCAA<br>CAGCCAGGCGCAUGUGUGGAACGUUAUCCCGCAAUUCUU<br>UGGUUU**UAUU**ACCUUUGCCAUCGCGGGCGUGGCGGUAU<br>GUCACCGUCACCCGUUUGACCAGCCGGAAGCCGAGCAGG<br>AACUGGCGGAUGGUUACCACAUUGAA**UAUU**CCGGUAUGA<br>AGUUCGGUCUGUUCUUCGUGGGUGAGUACAUCGGGAUUG<br>UGACCAUCUCUGCAUUGAUGGUGACGCUGUUCUUCGGUG

		GCUGGCAAGGCCCGUUGUUACCGCCAUUCAUCUGGUUCG<br>CGCUGAAAACCGCGUUCUUUAUGAUGAUGUUCAUUUUGA<br>UUCGUGCGUCGUUACCGCGUCCGCGUUAUGACCAGGUAA<br>UGUCCUUCGGCUGGAAAAUCUGCCUGCCGCUGACGCUGA<br>UCAACUUGCUGGUAACGGCGGCUGUCAUUCUCUGGCAGG<br>CGCAAUAA
	*nuo*I	AUGACCUUAAAAGAAUUGUUAGUAGGUUUCGGCACCCAG<br>GUUCGUAG**UAUU**UGGAUGAUCGGCCUGCACGCGUUCGCC<br>AAACGCGAAACGCGAAUGUACCCGGAAGAGCCGGUCUAU<br>CUGCCGCCCCGUUAUCGUGGUCGUAUCGUUCUGACCCGC<br>GACCCGGACGGCGAAGAGCGUUGCGUAGCCUGUAACCUC<br>UGCGCGGUAGCCUGCCCGGUCGGCUGUAUCUCGCUGCAA<br>AAAGCAGAAACCAAAGACGGUCGCUGGUACCCGGAAUUUU<br>UCCGCAUCAACUUCUCACGCUGCAUUUUCUGUGGUCUGU<br>GCGAAGAAGCCUGUCCGACCACGGCGAUUCAGUUAACCC<br>CGGAUUUCGAAAUGGGGGAAUACAAGCGCCAGGAUCUGG<br>UUUACGAGAAAGAGGAUCUGCUGAUCUCCGGUCCGGGCA<br>AAUACCCGGAAUAUAACUUCUACCGGAUGGCAGGUAUGG<br>CAAUCGACGGCAAAGAUAAGGGCGAAGCAGAGAACGAAG<br>CCAAGCCUAUCGACGUCAAGAGCCUGUUACCGUAA
	*nuo*J	AUCGAACAGGAACGCCAGUGGCUGAAACCGCAGGUGUGG<br>AUUGGUCCGGCAAUUUUGUCGGCCAUCAUGCUGGUGGUG<br>AUUGUUUACGCCAUCCUCGGUGUUAACGAUCAGGGUAUC<br>GACGGUACGCCAAUCAGUGCUAAAGCAGUGGG**UAUU**ACG<br>CUGUUCGGGCCUUACGUACUGGCGGUGGAACUGGCUUCU<br>AUGCUGCUGCUCGCAGGUCUGGUUGUGGCCUUCCACGUC<br>GGUCGUGAAGAGCGUGCGGGUGAAGUGCUGAGCAAUCGU<br>AAAGACGACAGCGCGAAAAGAAAAACGGAGGAGCACGCAU<br>GA
	*nuo*K	AUGAUCCCCUUACAACAUGGACUGAUCCUCGCGGCAAUC<br>U**UAUU**CGUUCUUGGCUUAACCGGUCUGGUUAUCCGUCGC<br>AAUCUGCUGUUUAUGUUGAUUGGUCUGGAAAUCAUGAUU<br>AACGCCUCCGCGCUGGCCUUCGUGGUCGCCGGAAGCUAC<br>UGGGGCCAGACCGACGGUCAGGUGAUGUACAUUCUCGCC<br>AUCAGCCUCGCGGCGGCAGAAGCGAGUAUCGGCCUUGCG<br>CUGCUGCUGCAACUUCACCGUCGUCGCCAGAACCUGAAC<br>AUCGAUUCAGUAAGUGAGAUGCGCGGAUGA
	*nuo*L	AUGAACAUGCUUGCCUUAACCAU**UAUU**UUGCCAUUGAUU<br>GGCUUCGUCCUGCUGGCAUUCUCCCGUGGGCGCUGGUC<br>UGAAAACGUCUCGGCGAUCGUCGGCGUAGGCUCUGUGGG<br>CCUGGCGGCGCUGGUAACCGCCUUUAUCGGCGUUGAUUU<br>CUUCGCUAACGGCGAGCAGACAUACAGCCAGCCGCUGUG<br>GACGUGGAUGUCGGUAGGCGACUUUAACAUCGGUUUUAA<br>CCUGGUGCUGGACGGCCUGUCGCUGACCAUGCUCUCGG<br>UGGUCACUGGUGUGGGUUUCCU**UAUU**CACAUGUACGCCU<br>CCUGGUAUAUGCGCGGUGAAGAGGGCUACUCUCGCUUCU<br>UCGCUUACACCAACCUGUUCAUCGCCAGCAUGGUGGUUC<br>UGGUGCUUGCCGACAACCUGCUGCUGAUGUACCUCGGCU<br>GGGAAGGCGUGGGCCUGUGCUCCUAUCUGCUGAUCGGG<br>UUC**UAUU**ACACCGAUCCGAAGAAUGGCGCAGCGGCAAUG<br>AAAGCGUUCGUCGUGACCCGUGUGGGUGACGUGUUCCUC<br>GCUUUCGCACUGUUCAUUCUUUACAACGAACUGGGCACC<br>CUGAACUUCCGCGAAAUGGUGGAACUGGCACCAGCGCAC<br>UUUGCUGACGGCAAUAACAUGCUGAUGUGGGCGACGCUG<br>AUGCUGCUGGGCGGUGCGGUCGGUAAAUCUGCGCAGUU

		GCCGUUGCAGACAUGGCUUGCUGACGCGAUGGCGGGCC<br>CGACGCCUGUCUCCGCGCUGAUCCACGCCGCAACGAUGG<br>UAACCGCGGGUGUCUACCUGAUCGCCCGUACCCACGGCC<br>UGUUCCUGAUGACGCCGGAAGUUCUGCAUCUGGUGGG**UA**<br>**UU**GUCGGGGCGGUUACGCUGCUGCUGGCCGGUUUUGCC<br>GCGCUGGUACAGACCGACAUCAAACGUGUUCUCGCUUAC<br>UCUACCAUGAGCCAGAUUGGCUACAUGUUCCUCGCGCUU<br>GGCGUGCAGGCAUGGGAUGCGGCGAUUUUCCACUUGAUG<br>ACUCACGCGUUCUUUAAAGCGCUGCUGUUCCUGGCAUCC<br>GGUUCCGUCAUUCUGGCCUGCCAUCACGAACAGAACAUC<br>UUCAAGAUGGGCGGUCUGCGUAAAUC**UAUU**CCGCUGGUU<br>UAUCUCUGCUUCCUGGUGGGCGGCGCAGCACUGUCGGCA<br>CUGCCGCUGGUCACUGCGGGCUUCUUCAGUAAGGAUGAG<br>AUCCUCGCGGGUGCGAUGGCGAAUGGUCAUAUCAAUCUG<br>AUGGUGGCAGGUCUGGUCGGUGCGUUUAUGACCUCGCU<br>CUACACCUUCCGUAUGAUUUUCAUCGUCUUCCACGGAAAA<br>GAACAAAUUCACGCUCACGCCGUGAAAGGGGUAACUCACA<br>GCCUGCCGCUGAUUGUGCUGCUGAUCCUUUCCACCUUCG<br>UUGGCGCACUGAUUGUACCGCCGCUGCAGGGCGUGCUUC<br>CGCAAACGACGGAACUGGCGCACGGCAGCAUGUUGACCC<br>UGGAAAUUACCUCUGGCGUGGUCGCGGUGGUCGGCAUUC<br>UGCUGGCAGCCUGGCUGUGGCUGGGUAAACGUACUCUG<br>GUGACCUCCAUCGCCAACAGUGCGCCGGGCCGCCUGCUG<br>AGUACCUGGUGGUACAACGCCUGGGGCUUUGACUGGCUG<br>UACGACAAAGUGUUCGUCAAGCCGUUCCUGGG**UAUU**GCC<br>UGGUUGCUGAAACGCGAUCCGCUGAACUCAAUGAUGAAC<br>AUCCCGGCGGUUCUUUCCCGCUUUGCAGGUAAAGGUCUG<br>CUGUUAAGCGAGAACGGUUAUCUGCGCUGGUAUGUGGCA<br>UCCAUGAGCAUCGGUGCGGUCGUGGUGCUGGCACUGUU<br>GAUGGUACUGCGUUGA
	*nuo*M	AUGU**UAUU**ACCCUGGUUAAUACUAAUCCCCUUUAUCGGC<br>GGCUUCCUGUGCUGGCAGACCGAACGCUUUGGCGUCAAG<br>GUGCCGCGCUGGAUCGCGCUGAUCACCAUGGGAUUGACG<br>CUGGCGCUGUCGCUGCAACUGUGGUUGCAGGGCGGU**UA**<br>**UU**CACUGACGCAAUCCGCCGGAAUUCCGCAGUGGCAGUC<br>UGAAUUCGACAUGCCGUGGAUCCCGCGUUUUGGUAUCUC<br>**UAUU**CAUCUCGCCAUUGACGGUCUGUCGUUGCUGAUGGU<br>CGUGCUGACCGGUCUGCUCGGUGUGCUGGCGGUACUCU<br>GUUCGUGGAAAGAGAUCGAAAAAUAUCAGGGCUUCUUCC<br>ACCUCAACCUGAUGUGGAUCCUGGGCGGCGUUAUCGGCG<br>UGUUCCUUGCCAUCGACAUGUUCCUGUUCUUCUUCUUCU<br>GGGAAAUGAUGCUGGUGCCGAUGUACUUCCUGAUCGCAC<br>UGUGGGGGCAUAAAGCCUCUGACGGUAAAACGCGUAUCA<br>CGGCGGCAACCAAGUUCUUCAUUUACACCCAGGCGAGUG<br>GUCUGGUGAUGUUGAUUGCCAUCCUGGCGCUGGUUUUU<br>GUUCACUACAAUGCGACCGGCGUCUGGACCUUCAACUAU<br>GAAGAGCUGUUGAAUACGCCAAUGUCCAGUGGUGUGGAA<br>UAUCUGUUGAUGCUGGGCUUCUUCAUCGCGUUUGCGGUG<br>AAAAUGCCGGUGGUUCCGCUGCAUGGCUGGCUGCCGGAU<br>GCGCACUCCCAGGCUCCGACCGCCGGUUCCGUUGACCUC<br>GCGGGGAUCUUGCUGAAAACUGCCGCUUACGGUCUGCUG<br>CGUUUCUCCCUGCCGCUGUUCCCGAACGCGUCGGCAGAG<br>UUCGCGCCAAUCGCUAUGUGGCUGGGUGUUAUCGGCAUC<br>UUCUACGGUGCGUGGAUGGCCUUCGCCCAGACCGAUAUC

		AAACGUCUGAUCGCCUACACCUCGGUUUCCCACAUGGGC<br>UUCGUGCUGAUUGCUAUCUACACCGGCAGCCAGUUGGCC<br>UACCAGGGCGCGGUAAUUCAGAUGAUUGCGCACGGCUUG<br>UCGGCGGCGGGUCUGUU**UAUU**CUUUGUGGUCAGCUUUA<br>UGAACGUAUCCAUACCCGCGACAUGCGCAUGAUGGGCGG<br>UCUGUGGAGCAAGAUGAAAUGGCUGCCAGCACUGUCGCU<br>GUUCUUUGCGGUGGCAACGCUUGGGAUGCCUGGCACCG<br>GUAACUUCGUCGGCGAAUUUAUGAUUCUGUUCGGCAGCU<br>UCCAGGUUGUCCCGGUGAUUACCGU**UAUU**UCUACCUUUG<br>GGCUGGUCUUUGCAUCUGUU**UAUU**CGCUGGCGAUGUUG<br>CAUCGCGCUUACUUCGGUAAAGCGAAAAGCCAGAUUGCC<br>AGCCAGGAACUGCCAGGGAUGUCGCUGCGUGAGCUGUUU<br>AUGAUCCUGUUGCUGGUGGUGCUGCUGGUACUGCUGGG<br>CUUCUAUCCGCAGCCGAUUCUGGAUACCUCGCACUCCGC<br>GAUUGGCAAUAUCCAGCAGUGGUUUGUUAAUUCCGUUAC<br>UACUACAAGGCCGUAA
	*nuo*N	AUGACAAUAACUCCACAAAACCUGAUCGCACUGCUACCGU<br>UGCUGAUCGUCGGCUUGACGGUGGUGGUUGUGAUGCUC<br>UCCAUUGCGUGGCGACGCAAUCAUUUCCUCAACGCUACG<br>CUCUCGGU**UAUU**GGGCUUAACGCGGCGCUGGUUUCGCU<br>CUGGUUUGUUGGCCAGGCGGGCGCUAUGGACGUUACGC<br>CGCUGAUGCGCGUUGAUGGUUUCGCCAUGCUUUACACCG<br>GGCUGG**UAUU**GUUGGCGAGCCUCGCCACCUGUACUUUCG<br>CCUACCCGUGGCUUGAAGGCUAUAACGACAACAAGGAUG<br>AGUUCUACCUGUUGGUGUUAAUUGCCGCGCUGGGCGGGA<br>UCCUGCUGGCGAAUGCCAACCAUCUGGCGUCUCUGUUCC<br>UCGGUAUCGAACUGAUCUCUUUGCCGCUGUUUGGCCUGG<br>UCGGUUACGCUUUCCGCCAGAAACGUUCACUGGAAGCCA<br>GUAUCAAAUACACCAUCCUUUCUGCCGCAGCGUCUUCUU<br>UCCUGCUGUUUGGUAUGGCGCUGGUGUAUGCGCAGUCU<br>GGCGACCUGUCGUUUGUCGCGUUGGGUAAAAACCUUGGC<br>GACGGUAUGCUCAACGAGCCGCUGUUGCUGGCAGGUUUC<br>GGCCUGAUGAUUGUUGGCCUCGGCUUCAAACUCUCUCUG<br>GUGCCGUUCCACCUGUGGACGCCAGACGUAUACCAGGGC<br>GCGCCUGCGCCGGUUUCCACUUUCCUGGCGACGGCGAG<br>CAAAAUCGCUAUCUUCGGUGUGGUGAUGCGUCUGUUCCU<br>CUACGCACCGGUGGGUGACAGCGAAGCGAUUCGCGUGGU<br>GCUGGCGAUUAUCGCCUUUGCCUCCAUCAUCUUCGGUAA<br>CCUGAUGGCGCUGAGCCAGACCAAUAUCAAACGUCUGCU<br>CGGUUACUCAUCUAUCUCUCACCUCGGCUAUCUGCUGGU<br>AGCGCUGAUUGCGCUGCAAACCGGCGAGAUGUCGAUGGA<br>AGCGGUAGGGGUUUACCUGGCCGGUUAUCUGUUCAGCAG<br>CCUCGGCGCGUUCGGCGUGGUCAGCCUGAUGUCCAGCC<br>CGUAUCGUGGCCCGGAUGCUGAUUCCCUGUUCUCUUACC<br>GCGGUCUGUUCUGGCAUCGUCCGAUCCUCGCGGCAGUGA<br>UGACGGUGAUGAUGCUGUCUCUGGCCGGUAUCCCGAUGA<br>CGCUGGGCUUUAUCGGUAAGUUCUACGUGCUGGCGGUC<br>GGUGUCCAGGCACACUUGUGGUGGCUGGUGGGUGCCGU<br>GGUUGUCGGUUCGGCAAUCGGCCUCUACUACUACCUGCG<br>CGUGGCGGUGAGCCUGUACCUGCACGCCCCGGAACAACC<br>GGGUCGCGAUGCACCAUCAAACUGGCAGUACAGCGCGGG<br>CGGUAUCGUGGUGCUGAUUUCUGCACUGUUGGUACUGGU<br>GCUGGGUGUAUGGCCACAACCGCUGAUUAG**UAUU**GUGCG<br>UUUGGCAAUGCCGCUGAUGUAA

Succinate dehydrogenase (complexe II)	*sdh*C	AUGAUAAGAAAUGUGAAAAAACAAAGACCUGUUAAUCUGG<br>ACCUACAGACCAUCCGGUUCCCCAUCACGGCGAUAGCGU<br>CCAUUCUCCAUCGCGUUUCCGGUGUGAUCACCUUUGUUG<br>CAGUGGGCAUCCUGCUGUGGCUUCUGGGUACCAGCCUCU<br>CUUCCCCUGAAGGUUUCGAGCAAGCUUCCGCGAUUAUGG<br>GCAGCUUCUUCGUCAAAUUUAUCAUGUGGGGCAUCCUUA<br>CCGCUCUGGCGUAUCACGUCGUCGUAGG**UAUU**CGCCACA<br>UGAUGAUGGAUUUUGGCUAUCUGGAAGAAACAUUCGAAG<br>CGGGUAAACGCUCCGCCAAAAUCUCCUUUGU**UAUU**ACUG<br>UCGUGCUUUCACUUCUCGCAGGAGUCCUCGUAUGGUAA
	*sdh*D	AUGGUAAGCAACGCCUCCGCAUUAGGACGCAAUGGCGUA<br>CAUGAUUUCAUCCUCGUUCGCGCUACCGCUAUCGUCCUG<br>ACGCUCUACAUCAUUUAUAUGGUCGGUUUUUUCGCUACC<br>AGUGGCGAGCUGACA**UAUU**AAGUCUGGAUCGGUUUCUUC<br>GCCUCUGCGUUCACCAAAGUGUUCACCCUGCUGGCGCUG<br>UUUUCUAUCUUGAUCCAUGCCUGGAUCGGCAUGUGGCAG<br>GUGUUGACCGACUACGUUAAACCGCUGGCUUUGCGCCUG<br>AUGCUGCAACUGGUGAUUGUCGUUGCACUGGUGGUUUAC<br>GUGAUUUAUGGAUUCGUUGUGGUGUGGGGUGUGUGA
	*sdh*A	AUGAAAUUGCCAGUCAGAGAAUUUGAUGCAGUUGUGAUU<br>GGUGCCGGCGGCGCAGGUAUGCGCGCGGCGCUGCAAAU<br>UUCCCAGAGCGGCCAGACCUGUGCGCUGCUCUCUAAAGU<br>CUUCCCGACCCGUUCCCAUACCGUUUCUGCGCAAGGCGG<br>CAUUACCGUUGCGCUGGGUAAUACCCAUGAAGAUAACUG<br>GGAAUGGCAUAUGUACGACACCGUAAAAGGGUCGGACUA<br>UAUCGGUGACCAGGACGCGAUUGAAUAUAUGUGUAAAAC<br>CGGGCCGGAAGCGAUUCUGGAACUCGAACACAUGGGCCU<br>GCCGUUCUCGCGUCUUGAUGAUGGUCGUAUCUAUCAACG<br>UCCGUUUGGCGGUCAGUCGAAAAACUUCGGCGGCGAGCA<br>GGCGGCACGCACUGCGGCAGCGGCUGACCGUACCGGUCA<br>CGCACUGUUGCACACGCUUUAUCAGCAGAACCUGAAAAAC<br>CACACCACCAUUUUCUCCGAGUGGUAUGCGCUGGAUCUG<br>GUGAAAAACCAGGAUGGCGCGGUGGUGGGUUGUACCGCA<br>CUGUGCAUCGAAACCGGUGAAGUGGUU**UAUU**UCAAAGCC<br>CGCGCUACCGUGCUGGCGACUGGCGGGGCAGGGCG**UAU**<br>**U**UAUCAGUCCACCACUAACGCCCACAUUAACACUGGCGAC<br>GGUGUCGGCAUGGCUAUCCGCGCAGGCGUACCGGUGCA<br>GGAUAUGGAAAUGUGGCAAUUCCACCCGACCGGCAUUGC<br>CGGUGCGGGCGUACUGGUCACCGAAGGUUGCCGUGGUG<br>AAGGCGGUUAUCUGCUGAACAAACAUGGCGAACGCUUUA<br>UGGAGCGUUAUGCGCCGAACGCCAAAGACCUGGCGGGCC<br>GUGAUGUGGUGGCGCGUUCCAUCAUGAUUGAAAUCCGUG<br>AAGGUCGCGGCUGUGAUGGUCCGUGGGGGCCACACGCG<br>AAACUGAAACUCGAUCACCUGGGUAAAGAAGUUCUCGAAU<br>CCCGUCUGCCGGGUAUCCUGGAGCUUUCCCGUACCUUCG<br>CUCACGUUGAUCCGGUGAAAGAGCCGAUUCCGGUUAUCC<br>CAACCUGUCACUACAUGAUGGGCGG**UAUU**CCGACCAAAG<br>UGACCGGUCAGGCGCUGACUGUGAAUGAGAAAGGCGAAG<br>AUGUGGUUGUUCCGGGGCUGUUCGCGGUAGGUGAAAUU<br>GCCUGCGUAUCGGUACACGGUGCUAACCGUCUGGGCGGU<br>AACUCGCUGCUGGACCUGGUGGUCUUUGGUCGCGCAGC<br>GGGUCUGCAUCUGCAAGAGUCUAUCGCCGAGCAGGGCGC<br>AUUGCGCGAUGCCAGCGAGUCUGAUGUUGAAGCAUCUCU<br>GGAUCGUCUGAACCGCUGGAACAAUAAUCGUAACGGUGA

		AGAUCCGGUGGCGAUUCGUAAAGCGCUGCAAGAAUGUAU<br>GCAGCAUAACUUCUCGGUCUUCCGUGAAGGUGAUGCGAU<br>GGCGAAAGGGCUUGAGCAGUUGAAAGUUAUCCGCGAGCG<br>UCUGAAAAAUGCCCGUCUGGAUGACACUUCCAGCGAGUU<br>CAACACCCAGCGCGUUGAGUGUCUGGAACUGGAUAACCU<br>GAUGGAAACGGCGUAUGCAACGGCUGUUUCUGCCAACUU<br>CCGUACCGAAAGCCGUGGCGCGCAUAGCCGCUUCGACUU<br>CCCGGAUCGUGAUGAUGAAAACUGGCUGUGCCACUCCCU<br>GUAUCUGCCAGAGUCGGAAUCCAUGACGCGCCGAAGCGU<br>CAACAUGGAACCGAAACUGCGCCCGGCAUUCCCGCCGAA<br>GAUUCGUACUUACUAA
	*sdh*B	AUGAGACUCGAGUUUUCAAUUUAUCGCUAUAACCCGGAU<br>GUUGAUGAUGCUCCGCGUAUGCAGGAUUACACCCUGGAA<br>GCGGAAGAAGGUCGCGACAUGAUGUUGCUGGAUGCGCUU<br>AUCCAGCUGAAAGAGAAAGAUCCCAGCCUGUCGUUCCGC<br>CGCUCCUGCCGUGAAGGUGUGUGCGGUUCCGACGGUCU<br>GAACAUGAACGGCAAGAAUGGUCUGGCCUG**UAUU**ACCCC<br>GAUUUCGGCACUCAACCAGCCGGGCAAGAAGAUUGUGAU<br>UCGCCCGCUGCCAGGUUUACCGGUGAUCCGCGAUUUGGU<br>GGUAGACAUGGGACAAUUCUAUGCGCAAUAUGAGAAAAUU<br>AAGCCUUACCUGUUGAAUAAUGGACAAAAUCCGCCAGCUC<br>GCGAGCAUUUACAGAUGCCAGAGCAGCGCGAAAAACUCG<br>ACGGGCUGUAUGAAUG**UAUU**CUCUGCGCAUGUUGUUCAA<br>CCUCUUGUCCGUCUUUCUGGUGGAAUCCCGAUAAGUUUA<br>UCGGCCCGGCAGGCUUGUUAGCGGCAUAUCGUUUCCUGA<br>UCGAUAGCCGUGAUACCGAGACUGGCAGCCGCCUCGACG<br>GUUUGAGCGAUGCAUUCAGUG**UAUU**CCGCUGUCACAGCA<br>UCAUGAACUGCGUCAGUGUAUGUCCGAAGGGGCUGAACC<br>CGACGCGCGCCAUCGGCCAUAUCAAGUCGAUGUUGUUGC<br>AGCGUAAUGCGUAA
	*frd*A	GUGCAAACCUUUCAAGCCGAUCUUGCCAUUGUAGGCGCC<br>GGUGGCGCGGGAUUACGUGCUGCAAUUGCUGCCGCGCA<br>GGCAAAUCCAAAUGCAAAAAUCGCACUAAUCUCAAAAGUA<br>UACCCGAUGCGUAGCCAUACCGUUGCUGCAGAAGGGGGC<br>UCCGCCGCUGUCGCGCAGGAUCAUGACAGCUUCGAAUAU<br>CACUUUCACGAUACAGUAGCGGGUGGCGACUGGUUGUGU<br>GAGCAGGAUGUCGUGGAU**UAUU**UCGUCCACCACUGCCCA<br>ACCGAAAUGACCCAACUGGAACUGUGGGGGUGCCCAUGG<br>AGCCGUCGCCCGGAUGGUAGCGUCAACGUACGUCGCUUC<br>GGCGGCAUGAAAAUCGAGCGUACCUGGUUCGCCGCCGAU<br>AAGACCGGCUUCCAUAUGCUGCACACGCUGUUCCAGACC<br>UCUCUGCAAUUCCCGCAGAUCCAGCGUUUUGACGAACAU<br>UUCGUGCUGGA**UAUU**CUGGUUGAUGAUGGUCAUGUUCGC<br>GGCCUGGUAGCAAUGAACAUGAUGGAAGGCACGCUGGUG<br>CAGAUCCGUGCUAACGCGGUCGUUAUGGCUACCGGCGGU<br>GCGGGUCGCGUUUAUCGUUACAACACCAACGGCGGCAUC<br>GUUACCGGUGACGGUAUGGGUAUGGCGCUAAGCCACGGC<br>GUUCCGCUGCGUGACAUGGAAUUCGUUCAGUAUCACCCA<br>ACCGGUCUGCCAGGUUCCGGUAUCCUGAUGACCGAAGGC<br>UGCCGUGGUGAAGGCGG**UAUU**CUGGUCAACAAAAAUGGC<br>UACCGUUAUCUGCAAGAUUACGGCAUGGGCCCGGAAACU<br>CCGCUGGGCGAGCCGAAAAACAAAUAUAUGGAACUGGGU<br>CCACGCGACAAAGUUUCUCAGGCCUUCUGGCACGAAUGG<br>CGUAAAGGCAACACCAUCUCCACGCCGCGUGGUGAUGUG<br>GUUUAUCUCGACCUGCGUCACCUCGGCGAGAAAAAACUG

		CAUGAACGUCUGCCGUUCAUCUGCGAACUGGCGAAAGCG<br>UACGUUGGCGUCGAUCCGGUUAAAGAACCGAUUCCGGUA<br>CGUCCGACCGCACACUACACCAUGGGCGGUAUCGAAACC<br>GAUCAGAACUGUGAAACCCGCAUUAAAGGUCUGUUCGCC<br>GUGGGUGAAUGUUCCUCUGUUGGUCUGCACGGUGCAAAC<br>CGUCUGGGCUCCAACUCCCUGGCGGAACUGGUGGUCUUC<br>GGCCGUCUGGCCGGUGAACAAGCGACAGAGCGUGCAGCA<br>ACUGCCGGUAAUGGCAACGAAGCGGCAAUUGAAGCGCAG<br>GCAGCUGGCGUUGAACAACGUCUGAAAGAUCUGGUUAAC<br>CAGGAUGGCGGCGAAAACUGGGCGAAGAUCCGCGACGAA<br>AUGGGCCUGGCAAUGGAAGAAGGUUGCGGUAUCUACCGU<br>ACGCCGGAACUGAUGCAGAAAACCAUCGACAAGCUGGCA<br>GAGCUGCAGGAACGCUUCAAGCGCGUGCGCAUCACCGAC<br>ACUUCCAGCGUGUUCAACACCGACCUGCUCUACACCAUU<br>GAACUGGGCCACGGUCUGAACGUUGCUGAAUGUAUGGCG<br>CACUCCGCAAUGGCACGUAAAGAGUCCCGCGGCGCACAC<br>CAGCGUCUGGACGAAGGUUGCACCGAGCGUGACGACGUC<br>AACUUCCUCAAACACACCCUCGCCUUCCGCGAUGCUGAU<br>GGCACGACUCGCCUGGAGUACAGCGACGUGAAGAUUACU<br>ACGCUGCCGCCAGCUAAACGCGUUUACGGUGGCGAAGCG<br>GAUGCAGCCGAUAAGGCGGAAGCAGCCAAUAAGAAGGAG<br>AAGGCGAAUGGCUGA
	*frd*B	AUGGCUGAGAUGAAAAACCUGAAAAUUGAGGUGGUGCGC<br>UAUAACCCGGAAGUCGAUACCGCACCGCAUAGCGCAUUC<br>UAUGAAGUGCCUUAUGACGCAACUACCUCAUUACUGGAU<br>GCGCUGGGCUACAUCAAAGACAACCUGGCACCGGACCUG<br>AGCUACCGCUGGUCCUGCCGUAUGGCGAUUUGUGGUUCC<br>UGCGGCAUGAUGGUUAACAACGUGCCAAAACUGGCAUGU<br>AAAACCUUCCUGCGUGAUUACACCGACGGUAUGAAGGUU<br>GAAGCGUUAGCUAACUUCCCGAUUGAACGCGAUCUGGUG<br>GUCGAUAUGACCCACUUCAUCGAAAGUCUGGAAGCGAUC<br>AAACCGUACAUCAUCGGCAACUCCCGCACCGCGGAUCAG<br>GGUACUAACAUCCAGACCCCGGCGCAGAUGGCGAAGUAU<br>CACCAGUUCUCCGGUUGCAUCAACUGUGGUCUGUGCUAC<br>GCCGCGUGCCCGCAGUUUGGCCUGAACCCAGAGUUCAUC<br>GGUCCGGCUGCCAUUACGCUGGCGCAUCGUUAUAACGAA<br>GAUAGCCGCGACCACGGUAAGAAGGAGCGUAUGGCGCAG<br>UUGAACAGCCAGAACGGCGUAUGGAGCUGUACUUUCGUG<br>GGC**UAUU**GCUCCGAAGUCUGCCCGAAACACGUCGAUCCG<br>GCUGCGGCCAUUCAGCAGGGCAAAGUAGAAAGUUCGAAA<br>GACUUUCUUAUCGCGACCCUGAAACCACGCUAA
	*frd*C	AUGACGACUAAACGUAAACCGUAUGUACGGCCAAUGACGU<br>CCACCUGGUGGAAAAAAUUGCCGUUUUAUCGCUUUUACA<br>UGCUGCGCGAAGGCACGGCGGUUCCGGCUGUGUGGUUC<br>AGCAUUGAACUGAUUUUCGGGCUGUUUGCCCUGAAAAAU<br>GGCCCGGAAGCCUGGGCGGGAUUCGUCGACUUUUUACAA<br>AACCCGGU**UAUU**GUGAUCAUUAACCUGAUCACUCUGGCG<br>GCAGCUCUGCUGCACACCAAAACCUGGUUUGAACUGGCA<br>CCGAAAGCGGCCAAUAUCAUUGUAAAAGACGAAAAAAUGG<br>GUCCAGAGCCAAUUAUCAAAAGUCUCUGGGCGGUAACUG<br>UGGUUGCCACCAUCGUAAUCCUGUUUGUUGCCCUGUACU<br>GGUAA
	*frd*D	GGUGGUAUGUGGAGCGCAAU**UAUU**GCGCCGGUGAUGAUC<br>CUGCUGGUGGG**UAUU**CUGCUGCCGCUGGGGCUGUUUCC

		GGGUGAUGCACUGAGCUACGAGCGCGUUCUGGCGUUCG<br>CGCAGAGCUUCAUUGGUCGCG**UAUU**CCUGUUCCUGAUGA<br>UCGUUCUGCCGCUGUGGUGUGGUUUACACCGUAUGCACC<br>ACGCGAUGCACGAUCUGAAAAUCCACGUACCUGCGGGCA<br>AAUGGGUUUUCUACGGUCUGGCUGCUAUCCUGACAGUUG<br>UCACGCUGAUUGGUGUCGUUACAAUCUAA
Cytochrome bd complex (complexe III)	*cyd*A	AUGUUAGAUAUAGUCGAACUGUCGCGCUUACAGUUUGCC<br>UUGACCGCGAUGUACCACUUCCUUUUUGUGCCACUGACG<br>CUCGGUAUGGCGUUCCUGCUGGCCAUUAUGGAAACGGUC<br>UACGUCCUCUCCGGCAAACAGAUUUAUAAAGAUAUGACCA<br>AGUUCUGGGGCAAGUUGUUUGGUAUCAACUUCGCUCUGG<br>GUGUGGCUACCGGUCUGACCAUGGAGUUCCAGUUCGGGA<br>CUAACUGGUCUUAC**UAUU**CCCACUAUGUAGGGGAUAUCU<br>UCGGUGCGCCGCUGGCAAUCGAAGGUCUGAUGGCCUUCU<br>UCCUCGAAUCCACCUUUGUAGGUCUGUUCUUCUUCGGUU<br>GGGAUCGUCUGGGUAAAGUUCAGCAUAUGUGUGUCACCU<br>GGCUGGUGGCGCUCGGUUCAAACCUGUCCGCACUGUGGA<br>UUCUGGUUGCGAACGGCUGGAUGCAAAACCCAAUCGCGU<br>CCGAUUUCAACUUUGAAACUAUGCGUAUGGAGAUGGUGA<br>GCUUCUCCGAGCUGGUGCUUAACCCGGUUGCUCAGGUGA<br>AAUUCGUUCACACUGUAGCGUCUGGUUAUGUGACUGGCG<br>CGAUGUUCAUCCUCGGUAUCAGCGCAUGGUAUAUGCUGA<br>AAGGUCGUGACUUCGCCUUCGCUAAACGCUCCUUUGCUA<br>UCGCUGCCAGCUUCGGUAUGGCUGCUGUUCUGUCUGU**UA**<br>**UU**GUUCUGGGUGAUGAAUCCGGCUACGAAAUGGGCGACG<br>UGCAGAAAACCAAACUGGCUGC**UAUU**GAAGCCGAGUGGG<br>AAACGCAACCUGCGCCUGCUGCCUUUACUCUGUUCGGCA<br>UUCCUGAUCAGGAAGAGGAGACGAACAAAUUUGCGAUUCA<br>GAUCCCUUACGCACUGGGCAUCAUUGCAACGCGUUCCGU<br>GGAUACCCCGGUUAUCGGCCUGAAAGAGCUGAUGGUGCA<br>GCAUGAAGAACGCAUUCGUAACGGGAUGAAGGCGUACUC<br>UCUGCUCGAACAACUGCGUUCUGGUUCUACCGACCAGGC<br>GGUUCGUGACCAGUUCAAUAGCAUGAAGAAAGACCUCGG<br>UUACGGUCUGCUGCUGAAACGCUAUACGCCAAACGUGGC<br>UGAUGCGACUGAAGCGCAGAUUCAACAGGCAACCAAAGAC<br>UCCAUCCCGCGUGUAGCGCCGCUGUACUUUGCGUUCCGU<br>AUCAUGGUGGCGUGUGGCUUCCUGCUUCUGGCAAUCAUC<br>GCGCUCUCUUUCUGGAGUGUCAUCCGCAACCGCAUUGGC<br>GAGAAAAAAUGGCUUCUGCGCGCCGCGCUGUACGG**UAUU**<br>CCGCUGCCGUGGAUUGCUGUAGAAGCGGGCUGGUUCGU<br>GGCUGAAUAUGGCCGCCAACCGUGGGCUAUCGGUGAAGU<br>GCUGCCGACAGCUGUGGCGAACUCGUCACUGACCGCAGG<br>CGAUCUCAUCUUCUCAAUGGUGCUGAUUUGCGGCCUGUA<br>UACCCUGUUCCUGGUGGCAGAAUUGUUCUUAAUGUUCAA<br>GUUUGCACGCCUCGGCCCAAGCAGCCUGAAAACCGGUCG<br>CUAUCACUUUGAGCAGUCUUCCACGACUACUCAGCCGGC<br>ACGCUAA
	*cyd*B	AUGAUCGAUUAUGAAG**UAUU**GCGUUUUAUCUGGUGGCUG<br>CUGGUUGGCGUUCUGCUGAUUGGUUUUGCAGUCACUGAC<br>GGUUUCGACAUGGGGGUGGGCAUGCUCACCCGUUUCCUC<br>GGUCGUAACGACACCGAGCGUCGAAUUAUGAUUAACUCC<br>AUUGCACCACACUGGGACGGUAACCAGGUUUGGCUGAUC<br>ACCGCGGGCGGCGCACUCUUUGCUGCCUGGCCGAUGGU<br>CUAUGCCGCUGCGUUCUCCGGCUUCUAUGUGGCGAUGAU<br>CCUCGUGCUGGCGUCUUUGUUCUUCCGUCCGGUCGGUU

		UUGACUACCGCUCCAAGAUUGAAGAAACCCGCUGGCGUA<br>ACAUGUGGGACUGGGGCAUCUUCAUUGGUAGCUUCGUUC<br>CGCCGCUGGUAAUUGGUGUAGCGUUCGGUAACCUGUUGC<br>AGGGCGUACCGUUCAACGUUGAUGAAUAUCUGCGUCUGU<br>ACUACACCGGUAACUUCUUCCAGUUGCUUAACCCGUUCG<br>GCCUGCUGGCAGGCGUGGUGAGCGUAGGGAUGAUCAUUA<br>CUCAGGGCGCAACCUAUCUGCAAAUGCGUACCGUGGGCG<br>AACUGCACCUGCGUACCCGUGCAACGGCUCAGGUGGCUG<br>CGCUGGUGACACUGGUCUGUUUCGCACUGGCUGGCGUAU<br>GGGUGAUGUACGG**<u>UAUU</u>**GAUGGUUAUGUCGUGAAAUCGA<br>CAAUGGACCAUUACGCAGCCUCUAACCCACUGAAUAAAGA<br>AGUGGUUCGUGAAGCUGGCGCAUGGCUGGUUAACUUCAA<br>CAACACGCCAAUUCUGUGGGC**<u>UAUU</u>**CCGGCACUGGGUGU<br>GGUUCUGCCGCUGCUGACCAUCCUGACUGCACGUAUGGA<br>UAAAGCCGCGUGGGCGUUUGUGUUCUCCUCCCUGACGCU<br>GGCCUGCAUCAUCCUGACAGCCGGUAUCGCAAUGUUCCC<br>GUUUGUGAUGCCGUCCAGCACCAUGAUGAACGCAAGUCU<br>GACAAUGUGGGAUGCAACUUCCAGCCAGCUGACGCUUAA<br>CGUCAUGACCUGGGUUGCGGUGGUUCUGGUACCGAUCAU<br>UCUGCUCUACACCGCCUGGUGUUACUGGAAAAUGUUCGG<br>UCGUAUCACCAAAGAAGA**<u>UAUU</u>**GAACGUAACACCCACUCU<br>CUGUACUAA
	*cyd*X	AUGUGG**<u>UAUU</u>**UCGCAUGGAUUCUGGGAACGCUUCUUGCC<br>UGUUCGUUUGGGGUAAUCACCGCGCUGGCGCUUGAACAC<br>GUCGAAUCAGGCAAAGCCGGUCAAGAAGACAUCUGA
Cytochrome c oxidase (complexe IV)	*cyo*D	AUGAGUCAUUCUACCGAUCACAGCGGCGCGUCCCAUGGC<br>AGCGUAAAAACCUACAUGACAGGCUUUAUCCUGUCGAUCA<br>UUCUGACGGUGAUUCCGUUCUGGAUGGUGAUGACAGGGG<br>CUGCCUCUCCGGCCGUAAUUCUGGGAACAAUCCUGGCAA<br>UGGCAGUGGUACAGGUUCUGGUGCAUCUGGUGUGCUUC<br>CUGCACAUGAAUACCAAAUCAGAUGAAGGCUGGAACAUGA<br>CGGCGUUUGUCUUCACCGUGCUAAUCAUCGC**<u>UAUU</u>**CUGG<br>UUGUAGGCUCCAUCUGGAUUAUGUGGAACCUCAACUACA<br>ACAUGAUGAUGCACUAA
	*cyo*C	AUGGCAACUGAUACUUUGACGCACGCGACUGCCCACGCG<br>CACGAACACGGGCACCACGAUGCAGGCGGAACCAAAAUCU<br>UCGGAUUUUGGAUCUACCUGAUGAGCGACUGCAUUCUGU<br>UCUCAAUCUUGUUUGCUACC**<u>UAUU</u>**CCGUUCUGGUGAACG<br>GCACCGCAGGCGGCCCGACAGGUAAGGACAUUUUCGAAC<br>UGCCGUUCGUUCUGGUUGAAACUUUCUUGCUGUUGUUCA<br>GCUCCAUCACCUACGGCAUGGCGGCUAUCGCCAUGUACA<br>AAAACAACAAAAGCCAGGUUAUCUCCUGGCUGGCGUUGAC<br>CUGGUUGUUUGGUGCCGGAUUUAUCGGGAUGGAAAUCUA<br>CGAAUUCCAUCACCUGAUUGUUAACGGCAUGGGUCCGGA<br>UCGCAGCGGCUUCCUGUCAGCGUUCUUUGCGCUGGUCG<br>GCACGCACGGUCUGCACGUCACUUCCGGUCUUAUCUGGA<br>UGGCGGUGCUGAUGGUACAAAUCGCUCGUCGCGGCCUGA<br>CCAGCACUAACCGUACCCGCAUCAUGUGCCUGAGCCUGU<br>UCUGGCACUUCCUGGAUGUGGUUUGGAUCUGUGUGUUCA<br>CUGUUGUUUAUCUGAUGGGGGCGAUGUAA
	*cyo*B	AUGUUCGGAAAAUUAUCACUUGAUGCAGUCCCGUUCCAU<br>GAACCUAUCGUCAUGGUUACGAUCGCUGGCAU**<u>UAUU</u>**UUG<br>GGAGGUCUGGCGCUCGUUGGCCUGAUCACUUACUUCGGU<br>AAGUGGACCUACCUGUGGAAAGAGUGGCUGACCUCCGUC

		GACCAUAAACGCCUCGGUAUCAUGUAUAUCAUCGUGGCG<br>AUUGUGAUGUUGCUGCGUGGUUUUGCUGACGCCAUUAUG<br>AUGCGUAGCCAGCAGGCUCUUGCCUCGGCGGGUGAAGCG<br>GGCUUCCUGCCACCUCACCACUACGAUCAGAUCUUCACC<br>GCGCACGGCGUGAUUAUGAUCUUCUUCGUGGCGAUGCCU<br>UUCGUUAUCGGUCUGAUGAACCUGGUGGUUCCGCUGCAG<br>AUCGGCGCGCGUGACGUUGCGUUCCCGUUCCUCAACAAC<br>UUAAGCUUCUGGUUUACUGUUGUUGGUGUGAUUCUGGUU<br>AACGUUUCUCUCGGCGUGGGCGAAUUUGCGCAGACCGGC<br>UGGCUGGCCUAUCCACCGCUAUCGGGAAUAGAGUACAGU<br>CCGGGAGUCGGUGUUGAUUACUGGAUAUGGAGUCUCCAG<br>CUAUCCGGUAUAGGUACGACGCUUACCGGUAUCAACUUC<br>UUCGUUACCAUUCUGAAGAUGCGCGCACCGGGCAUGACC<br>AUGUUCAAGAUGCCAG**UAUU**UACUUGGGCAUCACUGUGC<br>GCGAACGUACUGAU**UAUU**GCUUCCUUCCCAAUUCUGACG<br>GUUACCGUCGCGUUGUUGACCCUGGAUCGCUAUCUGGGC<br>ACCCAUUUCUUUACCAACGAUAUGGGUGGCAACAUGAUGA<br>UGUACAUCAACCUGAUUUGGGCCUGGGGCCACCCGGAAG<br>UUUACAUCCUGAUCCUGCCUGUUUUCGGUGUGUUCUCCG<br>AAAUUGCGGCAACCUUCUCGCGUAAACGUCUGUUUGGUU<br>AUACCUCGCUGGUAUGGGCAACCGUCUGUAUCACCGUGC<br>UGUCGUUCAUCGUUUGGCUGCACCACUUCUUUACGAUGG<br>GUGCGGGCGCGAACGUAAACGCCUUCUUUGGUAUCACCA<br>CAAUGAUUAUCGCCAUCCCGACCGGGGUGAAGAUCUUCA<br>ACUGGCUGUUCACCAUGUAUCAGGGCCGCAUCGUGUUCC<br>AUUCUGCGAUGCUGUGGACCAUCGGUUUUAUCGUCACUU<br>UCUCGGUGGGCGGGAUGACUGGCGUGCUGCUGGCAGUA<br>CCGGGCGCGGACUUCGUUCUGCAUAACAGCCUGUUCCUG<br>AUUGCGCACUUCCAUAACGUGAUCAUCGGCGGCGUGGUC<br>UUCGGCUGCUUCGCAGGGAUGACCUACUGGUGGCCUAAA<br>GCCUUCGGUUUCAAACUGAAUGAAACCUGGGGUAAACGC<br>GCGUUCUGGUUCUGGAUCAUCGGCUUCUUCGUUGCCUUU<br>AUGCCGCUGUAUGCGCUGGGCUUCAUGGGCAUGACCCGU<br>CGUUUGAGCCAGCAGAUUGACCCGCAGUUCCACACCAUG<br>CUGAUGAUUGCAGCUAGCGGUGCAGUACUGAUUGCGCUG<br>GG**UAUU**CUCUGCCUCGU**UAUU**CAGAUGUACGUUUC**UAUU**<br>CGCGACCGCGACCAGAACCGUGACCUGACUGGCGACCCG<br>UGGGGUGGCCGUACGCUGGAGUGGGCAACCUCUUCCCC<br>GCCUCCGUUCUAUAACUUUGCCGUUGUGCCGCACGUUCA<br>CGAGCGUGAUGCAUUCUGGGAAAUGAAAGAGAAAGGCGA<br>AGCGUAUAAAAAGCCUGACCACUAUGAAGAAAUUCAUAUG<br>CCGAAAAACAGCGGUGCAGGUAUCGUCAUUGCAGCUUUC<br>UCCACCAUCUUCGGUUUCGCCAUGAUCUGGCAUAUCUGG<br>UGGCUGGCGAUUGUUGGCUUCGCAGGCAUGAUCAUCACC<br>UGGAUCGUGAAAAGCUUCGACGAGGACGUGGAUUACUAC<br>GUGCCGGUGGCAGAAAUCGAAAAACUGGAAAACCAGCAUU<br>UCGAUGAGAUUACUAAGGCAGGGCUGAAAAAUGGCAACU<br>GA
	*cyo*A	AUGAGACUCAGGAAAUACAAUAAAAGUUUGGGAUGGUUGU<br>CAU**UAUU**UGCAGGCACUG**UAUU**GCUCAGUGGCUGUAAUU<br>CUGCGCUGUUAGAUCCCAAAGGACAGAUUGGUCUGGAGC<br>AACGUUCACUGAUACUGACGGCAUUUGGCCUGAUGUUGA<br>UUGUCGU**UAUU**CCCGCAAUCUUGAUGGCUGUUGGUUUCG<br>CCUGGAAGUAUCGUGCGAGCAAUAAAGAUGCUAAGUACA<br>GCCCGAACUGGUCACACUCCAAUAAAGUGGAAGCUGUGG

		UCUGGACGGUACCUAUCUUAAUCAUCAUCUUCCUUGCGG<br>UACUGACCUGGAAAACCACUCACGCUCUUGAGCCUAGCAA<br>GCCGCUGGCACACGACGAGAAGCCCAUUACCAUCGAAGU<br>GGUUUCCAUGGACUGGAAAUGGUUCUUCAUCUACCCGGA<br>ACAGGGCAUUGCUACCGUGAAUGAAAUCGCUUUCCCGGC<br>GAACACUCCGGUGUACUUCAAAGUGACCUCCAACUCCGU<br>GAUGAACUCCUUCUUCAUUCCGCGUCUGGGUAGCCAGAU<br>UUAUGCCAUGGCCGGUAUGCAGACUCGCCUGCAUCUGAU<br>CGCCAACGAACCCGGUACUUAUGACGGUAUCUCCGCCAG<br>CUACAGCGGGCCGGGCUUCUCAGGCAUGAAGUUCAAAGC<br>**UAUU**GCAACACCGGAUCGCGCCGAAUUCGACCAAUGGGU<br>CGCAAAAGCGAAACAGUCGCCGAACUCCAUGUCUGACAUG<br>GCAGCGUUCGAAAAACUGGCCGCGCCUAGCGAAUACAAC<br>CAGGUGGAA**UAUU**UCUCCAACGUGAAACCAGACUUGUUU<br>GCCGAUGUGAUUAACAAGUUUAUGGCUCACGGUAAGAGC<br>AUGGACAUGACCCAGCCAGAAGGUGAGCACAGUGCACAC<br>GAAGGUAUGGAAGGCAUGGACAUGAGCCACGCGGAAUCC<br>GCCCAUUAA
ATP synthase	*atp*A	AUGCAACUGAAUUCCACCGAAAUCAGCGAACUGAUCAAGC<br>AGCGCAUUGCUCAGUUCAAUGUUGUGAGUGAAGCUCACA<br>ACGAAGGUAC**UAUU**GUUUCUGUAAGUGACGGUGUUAUCC<br>GCAUUCACGGCCUGGCCGAUUGUAUGCAGGGUGAAAUGA<br>UCUCCCUGCCGGGUAACCGUUACGCUAUCGCACUGAACC<br>UCGAGCGCGACUCUGUAGGUGCGGUUGUUAUGGGUCCG<br>UACGCUGACCUUGCCGAAGGCAUGAAAGUUAAGUGUACU<br>GGCCGUAUCCUGGAAGUUCCGGUUGGCCGUGGCCUGCU<br>GGGCCGUGUGGUUAACACUCUGGGUGCACCAAUCGACGG<br>UAAAGGUCCGCUGGAUCACGACGGCUUCUCUGCUGUAGA<br>AGCAAUCGCUCCGGGCGUUAUCGAACGUCAGUCCGUAGA<br>UCAGCCGGUACAGACCGGUUAUAAAGCCGUUGACUCCAU<br>GAUCCCAAUCGGUCGUGGUCAGCGUGAAUUGAUCAUCGG<br>UGACCGUCAGACAGGUAAAACCGCACUGGCUAUCGAUGC<br>CAUCAUCAACCAGCGCGAUUCCGGUAUCAAAUGUAUCUAU<br>GUCGCUAUCGGCCAGAAAGCGUCCACCAUUUCUAACGUG<br>GUACGUAAACUGGAAGAGCACGGCGCACUGGCUAACACC<br>AUCGUUGUGGUAGCAACUGCGUCUGAAUCCGCUGCACUG<br>CAAUACCUGGCACCGUAUGCCGGUUGCGCAAUGGGCGAA<br>UACUUCCGUGACCGCGGUGAAGAUGCGCUGAUCAUUUAC<br>GAUGACCUGUCUAAACAGGCUGUUGCUUACCGUCAGAUC<br>UCCCUGCUGCUCCGUCGUCCGCCAGGACGUGAAGCAUUC<br>CCGGGCGACGUUUUCUACCUCCACUCUCGUCUGCUGGAG<br>CGUGCUGCACGUGUUAACGCCGAAUACGUUGAAGCCUUC<br>ACCAAAGGUGAAGUGAAAGGGAAAACCGGUUCUCUGACC<br>GCACUGCCGAUUAUCGAAACUCAGGCGGGUGACGUUUCU<br>GCGUUCGUUCCGACCAACGUAAUCUCCAUUACCGAUGGU<br>CAGAUCUUCCUGGAAACCAACCUGUUCAACGCCGG**UAUU**<br>CGUCCUGCGGUUAACCCGGG**UAUU**UCCGUAUCCCGUGUU<br>GGUGGUGCAGCACAGACCAAGAUCAUGAAAAAACUGUCC<br>GGUGGUAUCCGUACCGCUCUGGCACAGUAUCGUGAACUG<br>GCAGCGUUCUCUCAGUUUGCAUCCGACCUUGACGAUGCA<br>ACACGUAAGCAGCUUGACCACGGUCAGAAAGUGACCGAAC<br>UGCUGAAACAGAAACAGUAUGCGCCGAUGUCCGUUGCGC<br>AGCAGUCUCUGGUUCUGUUCGCAGCAGAACGUGGUUACC<br>UGGCGGAUGUUGAACUGUCGAAAAUUGGCAGCUUCGAAG

		CCGCUCUGCUGGCUUACGUCGACCGUGAUCACGCUCCGU<br>UGAUGCAAGAGAUCAACCAGACCGGUGGCUACAACGACG<br>AAAUCGAAGGCAAGCUGAAAGGCAUCCUCGAUUCCUUCAA<br>AGCAACCCAAUCCUGGUAA
	*atp*D	AUGGCUACUGGAAAGAUUGUCCAGGUAAUCGGCGCCGUA<br>GUUGACGUCGAAUUCCCUCAGGAUGCCGUACCGCGCGUG<br>UACGAUGCUCUUGAGGUGCAAAAUGGUAAUGAGCGUCUG<br>GUGCUGGAAGUUCAGCAGCAGCUCGGCGGCGGUAUCGUA<br>CGUACCAUCGCAAUGGGUUCCUCCGACGGUCUGCGUCGC<br>GGUCUGGAUGUAAAAGACCUCGAACACCCGAUUGAAGUC<br>CCGGUAGGUAAAGCGACUCUGGGCCGUAUCAUGAACGUA<br>CUGGGUGAACCGGUCGACAUGAAAGGCGAGAUCGGUGAA<br>GAAGAGCGUUGGGCGAUUCACCGCGCAGCACCUUCCUAC<br>GAAGAGCUGUCAAACUCUCAGGAACUGCUGGAAACCGGU<br>AUCAAAGUUAUCGACCUGAUGUGUCCGUUCGCUAAGGGC<br>GGUAAAGUUGGUCUGUUCGGUGGUGCGGGUGUAGGUAA<br>AACCGUAAACAUGAUGGAGCUCAUUCGUAACAUCGCGAUC<br>GAGCACUCCGGUUACUCUGUGUUUGCGGGCGUAGGUGAA<br>CGUACUCGUGAGGGUAACGACUUCUACCACGAAAUGACC<br>GACUCCAACGUUAUCGACAAAGUAUCCCUGGUGUAUGGC<br>CAGAUGAACGAGCCGCCGGGAAACCGUCUGCGCGUUGCU<br>CUGACCGGUCUGACCAUGGCUGAGAAAUUCCGUGACGAA<br>GGUCGUGACGUUCUGCUGUUCGUUGACAACAUCUAUCGU<br>UACACCCUGGCCGGUACGGAAGUAUCCGCACUACUGGGC<br>CGUAUGCCUUCAGCGGUAGGUUAUCAGCCGACCCUGGCG<br>GAAGAGAUGGGCGUUCUGCAGGAACGUAUCACCUCCACC<br>AAAACUGGUUCUAUCACCUCCGUACAGGCAGUAUACGUAC<br>CUGCGGAUGACUUGACUGACCCGUCUCCGGCAACCACCU<br>UUGCGCACCUUGACGCAACCGUGGUACUGAGCCGUCAGA<br>UCGCGUCUCUGGGUAUCUACCCGGCCGUUGACCCGCUGG<br>ACUCCACCAGCCGUCAGCUGGACCCGCUGGUGGUUGGUC<br>AGGAACACUACGACACCGCGCGUGGCGUUCAGUCCAUCC<br>UGCAACGUUAUCAGGAACUGAAAGACAUCAUCGCCAUCCU<br>GGGUAUGGAUGAACUGUCUGAAGAAGACAAACUGGUGGU<br>AGCGCGUGCUCGUAAGAUCCAGCGCUUCCUGUCCCAGCC<br>GUUCUUCGUGGCAGAAG**UAUU**CACCGGUUCUCCGGGUAA<br>AUACGUCUCCCUGAAAGACACCAUCCGUGGCUUUAAAGG<br>CAUCAUGGAAGGCGAAUACGAUCACCUGCCGGAGCAGGC<br>GUUCUACAUGGUCGGUUCCAUCGAAGAAGCUGUGGAAAA<br>AGCCAAAAAACUUUAA
	*atp*G	AUGGCCGGCGCAAAAGAGAUACGUAGUAAGAUCGCAAGC<br>GUCCAGAACACGCAAAAGAUCACUAAAGCGAUGGAGAUGG<br>UCGCCGCUUCCAAAAUGCGUAAAUCGCAGGAUCGCAUGG<br>CGGCCAGCCGUCCUUAUGCAGAAACCAUGCGCAAAGUGA<br>UUGGUCACCUUGCACACGGUAAUCUGGAAUAUAAGCACC<br>CUUACCUGGAAGACCGCGACGUUAAACGCGUGGGCUACC<br>UGGUGGUGUCGACCGACCGUGGUUUGUGCGGUGGUUUG<br>AACAUUAACCUGUUCAAAAAACUGCUGGCGGAAAUGAAGA<br>CCUGGACCGACAAAGGCGUUCAAUGCGACCUCGCAAUGA<br>UCGGCUCGAAAGGCGUGUCGUUCUUCAACUCCGUGGGCG<br>GCAAUGUUGUUGCCCAGGUCACCGGCAUGGGGGAUAACC<br>CUUCCCUGUCCGAACUGAUCGGUCCGGUAAAAGUGAUGU<br>UGCAGGCCUACGACGAAGGCCGUCUGGACAAGCUUUACA<br>UUGUCAGCAACAAAUU**UAUU**AACACCAUGUCUCAGGUUCC<br>GACCAUCAGCCAGCUGCUGCCGUUACCGGCAUCAGAUGA

		UGAUGAUCUGAAACAUAAAUCCUGGGAUUACCUGUACGAA<br>CCCGAUCCGAAGGCGUUGCUGGAUACCCUGCUGCGUCGU<br>UAUGUCGAAUCUCAGGUUUAUCAGGGCGUGGUUGAAAAC<br>CUGGCCAGCGAGCAGGCCGCCCGUAUGGUGGCGAUGAAA<br>GCCGCGACCGACAAUGGCGGCAGCCUGAUUAAAGAGCUG<br>CAGUUGGUAUACAACAAAGCUCGUCAGGCCAGCAUUACUC<br>AGGAACUCACCGAGAUCGUCUCGGGGGCCGCCGCGGUUU<br>AA
	*atp*H	AUGUCUGAAUU**UAUU**ACGGUAGCUCGCCCCUACGCCAAA<br>GCAGCUUUUGACUUUGCCGUCGAACACCAAAGUGUAGAA<br>CGCUGGCAGGACAUGCUGGCGUUUGCCGCCGAGGUAACC<br>AAAAACGAACAAAUGGCAGAGCUUCUCUCUGGCGCGCUU<br>GCGCCAGAAACGCUCGCCGAGUCGUUUAUCGCAGUUUGU<br>GGUGAGCAACUGGACGAAAACGGUCAGAACCUGAUUCGG<br>GUUAUGGCUGAAAAUGGUCGUCUUAACGCGCUCCCGGAU<br>GUUCUGGAGCAGUU**UAUU**CACCUGCGUGCCGUGAGUGAG<br>GCUACCGCUGAGGUAGACGUCAUUUCCGCUGCCGCACUG<br>AGUGAACAACAGCUCGCGAAAAUUUCUGCUGCGAUGGAAA<br>AACGUCUGUCACGCAAAGUUAAGCUGAAUUGCAAAAUCGA<br>UAAGUCUGUAAUGGCAGGCGUUAUCAUCCGAGCGGGUGA<br>UAUGGUCAUUGAUGGCAGCGUACGCGGUCGUCUUGAGCG<br>CCUUGCAGACGUCUUGCAGUCUUAA
	*atp*C	AUGGCAAUGACUUACCACCUGGACGUCGUCAGCGCAGAG<br>CAACAAAUGUUCUCUGGUCUGGUCGAGAAAAUCCAGGUAA<br>CGGGUAGCGAAGGUGAACUGGGGAUCUACCCUGGCCACG<br>CACCGCUGCUCACCGCCAUUAAGCCUGGUAUGAUUCGCA<br>UCGUGAAACAGCACGGUCACGAAGAGUUUAUCUAUCUGU<br>CUGGCGGCAUUCUUGAAGUGCAGCCUGGCAACGUGACCG<br>UUCUGGCCGACACCGCAAUUCGCGGCCAGGAUCUCGACG<br>AAGCGCGAGCCAUGGAAGCGAAACGUAAGGCUGAAGAGC<br>ACAUUAGCAGCUCUCACGGCGACGUAGAUUACGCUCAGG<br>CGUCUGCGGAACUGGCCAAAGCGAUCGCGCAGCUGCGCG<br>U**UAUU**GAGUUGACCAAAAAAGCGAUGUAA
	*atp*B	AUGGCUUCAGAAAAUAUGACGCCGCAGGAUUACAUAGGAC<br>ACCACCUGAAUAACCUUCAGCUGGACCUGCGUACAUUCUC<br>GCUGGUGGAUCCACAAAACCCCCCAGCCACCUUCUGGAC<br>AAUCAA**UAUU**GACUCCAUGUUCUUCUCGGUGGUGCUGGG<br>UCUGUUGUUCCUGGUUU**UAUU**CCGUAGCGUAGCCAAAAA<br>GGCGACCAGCGGUGUGCCAGGUAAGUUUCAGACCGCGAU<br>UGAGCUGGUGAUCGGCUUUGUUAAUGGUAGCGUGAAAGA<br>CAUGUACCAUGGCAAAAGCAAGCUGAUUGCUCCGCUGGC<br>CCUGACGAUCUUCGUCUGGG**UAUU**CCUGAUGAACCUGAU<br>GGAUUUACUGCCUAUCGACCUGCUGCCGUACAUUGCUGA<br>ACAUGUACUGGGUCUGCCUGCACUGCGUGUGGUUCCGUC<br>UGCGGACGUGAACGUAACGCUGUCUAUGGCACUGGGCG**U**<br>**AUU**UAUCCUGAUUCUGUUCUACAGCAUCAAAAUGAAAGGC<br>AUCGGCGGCUUCACGAAAGAGUUGACGCUGCAGCCGUUC<br>AAUCACUGGGCGUUCAUUCCUGUCAACUUAAUCCUUGAA<br>GGGGUAAGCCUGCUGUCCAAACCAGUUUCACUCGGUUUG<br>CGACUGUUCGGUAACAUGUAUGCCGGUGAGCUGAUUUUC<br>AUUCUGAUUGCUGGUCUGUUGCCGUGGUGGUCACAGUG<br>GAUCCUGAAUGUGCCGUGGGCCAUUUUCCACAUCCUGAU<br>CAUUACGCUGCAAGCCUUCAUCUUCAUGGUUCUGACGAU<br>CGUCUAUCUGUCGAUGGCGUCUGAAGAACAUUAA

	*atp*A	GUGAAUCUUAACGCAACAAUCCUCGGCCAGGCCAUCGCG<br>UUUGUCCUGUUCGUUCUGUUCUGCAUGAAGUACGUAUGG<br>CCGCCAU**<u>UAUU</u>**GGCAGCCAUCGAAAAACGUCAAAAAGAAA<br>UUGCUGACGGCCUUGCUUCCGCAGAACGAGCACAUAAGG<br>ACCUUGACCUUGCAAAGGCCAGCGCGACCGACCAGCUGA<br>AAAAAGCGAAAGCGGAAGCCCAGGUAAUCAUCGAGCAGG<br>CGAACAAACGCCGCUCGCAGAUUCUGGACGAAGCGAAAG<br>CUGAGGCAGAACAGGAACGUACUAAAAUCGUGGCCCAGG<br>CGCAGGCGGAAAUUGAAGCCGAGCGUAAACGUGCCCGUG<br>AAGAGCUGCGUAAGCAAGUUGCUAUCCUGGCUGUUGCUG<br>GCGCCGAGAAGAUCAUCGAACGUUCCGUGGAUGAAGCUG<br>CUAACAGCGACAUCGUGGAUAAACUUGUCGCUGAACUGU<br>AA
	*atp*E	AUGGAAAACCUGAA**<u>UAUU</u>**GAUCUGCUGUACAUGGCUGCC<br>GCUGUGAUGAUGGGUCUGGCGGCAAUCGGUGCUGCGAU<br>CGGUAUCGGCAUCCUCGGGGGUAAAUUCCUGGAAGGCGC<br>AGCGCGUCAACCUGAUCUGAUUCCUCUGCUGCGUACUCA<br>GUUCUUUAUCGUUAUGGGUCUGGUGGAUGCUAUCCCGAU<br>GAUCGCUGUAGGUCUGGGUCUGUACGUGAUGUUCGCUG<br>UCGCGUAG

TABLE S4 The mRNA of metabolic enzymes involved in glycolysis

Glycolysis		
Enzyme	Gene	Sequence (mRNA)
phosphofructokinase	*pfk*B	AUGUUAGCCGUCCUAAAUUUUAAAAACUUAGUUGACUUUG<br>AAGAACAGUUGCAAUUUCUUGACGAUUUAAGUCAUGCUGA<br>UGUCCAAGUCGAUCUGAAAAUUUCACCAACUUUGCCUGUA<br>CCCAAUGAUUUUGAGAAUUUUGAAAUCAUCAGUCAAAACU<br>UGACCAUCAAAGAGCGCACGGUCGGCGAAAUCUCGCCUA<br>GCGUUCUCCACCACCUUAACAUUUCAACCAGUUUCAUUGG<br>UCACUUGGAGCGUCGUAAGUCUUUGAACGAAGGCUUGCU<br>AAUCGUGCGUAAGCGUCUGGAAAAUGCGUUGGAAAAUGA<br>UAUCAAGCCAAU**UAUU**UCAGUUGGCCAAUACAGUAAUUUA<br>GAAUUGGUGACUGAAGAGCUUGAUAUCUUGUUGAAGGAU<br>CAACCAAUUACCAAGCCAAUCAU**UAUU**GCUUAUGAAUCAC<br>UUGCAUCAACCGAAAUGGGGCGUGCACUAUACUCAAUUG<br>AUGAAAUUCGCGACGUUCACAAUAAAAUUCGCGAGUUCAU<br>GACGACGCAUUACCCAGCCAUUGAUUACCAAUUAAUUUUG<br>GGUGGUCACAUGGACGAAGUUGGUUCACCAAUUGCCAAC<br>GCGAUUGGCUACGACGGCGUUCUGAUUGGUGAUCGUUAC<br>CACACACUCGCUUCGUUCCAACCCGUUUUGAAUGUACUUA<br>ACAACUUAAGUCGAUGA
aldolase	*fba*A	AUGUCUAAGAUUUUUGAUUUCGUAAAACCUGGCGUAAUCA<br>CUGGUGAUGACGUACAGAAAGUUUUCCAGGUAGCAAAAG<br>AAAACAACUUCGCACUGCCAGCAGUAAACUGCGUCGGUAC<br>UGACUCCAUCAACGCCGUACUGGAAACCGCUGCUAAAGU<br>UAAAGCGCCGGUUAUCGUUCAGUUCUCCAACGGUGGUGC<br>UUCCUUUAUCGCUGGUAAAGGCGUGAAAUCUGACGUUCC<br>GCAGGGUGCUGCUAUCCUGGGCGCGAUCUCUGGUGCGC<br>AUCACGUUCACCAGAUGGCUGAACAUUAUGGUGUUCCGG<br>UUAUCCUGCACACUGACCACUGCGCGAAGAAACUGCUGC<br>CGUGGAUCGACGGUCUGUUGGACGCGGGUGAAAAACACU<br>UCGCAGCUACCGGUAAGCCGCUGUUCUCUUCUCACAUGA<br>UCGACCUGUCUGAAGAAUCUCUGCAAGAGAACAUCGAAAU<br>CUGCUCUAAAUACCUGGAGCGCAUGUCCAAAAUCGGCAU<br>GACUCUGGAAAUCGAACUGGGUUGCACCGGUGGUGAAGA<br>AGACGGCGUGGACAACAGCCACAUGGACGCUUCUGCACU<br>GUACACCCAGCCGGAAGACGUUGAUUACGCAUACACCGAA<br>CUGAGCAAAAUCAGCCCGCGUUUCACCAUCGCAGCGUCC<br>UUCGGUAACGUACACGGUGUUUACAAGCCGGGUAACGUG<br>GUUCUGACUCCGACCAUCCUGCGUGAUUCUCAGGAAUAU<br>GUUUCCAAGAAACACAACCUGCCGCACAACAGCCUGAACU<br>UCG**UAUU**CCACGGUGGUUCCGGUUCUACUGCUCAGGAAA<br>UCAAAGACUCCGUAAGCUACGGCGUAGUAAAAAUGAACAU<br>CGAUACCGAUACCCAAUGGGCAACCUGGGAAGGCGUUCU<br>GAACUACUACAAAGCGAACGAAGCUUAUCUGCAGGGUCAG<br>CUGGGUAACCCGAAAGGCGAAGAUCAGCCGAACAAGAAAU<br>ACUACGAUCCGCGCGUAUGGCUGCGUGCCGGUCAGACUU<br>CGAUGAUCGCUCGUCUGGAGAAAGCAUUCCAGGAACUGA<br>ACGCGAUCGACGUUCUGUAA
	*fba*B	AUGACAGA**UAUU**GCACAGUUGCUUGGCAAAGACGCCGAC<br>AACCUUUUACAGCACCGUUGUAUGAC**UAUU**CCUUCUGAC<br>CAGCUUUAUCUCCCCGGACAUGACUACGUAGACCGCGUG<br>AUGAUUGACAAUAAUCGCCCGCCAGCGGUGUUACGUAAU

		AUGCAGACGUUGUACAACACUGGGCGUCUGGCUGGCACA<br>GGAUAUCUUUC**UAUU**CUGCCGGUUGACCAGGGCGUUGAG<br>CACUCUGCCGGAGCUUCAUUUGCUGCUAACCCGCUCUAC<br>UUUGACCCGAAAAACAUUGUUGAACUGGCGAUCGAAGCG<br>GGCUGUAACUGUGUGGCAUCAACUUACGGCGUGUUGGCG<br>UCGGUAUCGCGGCGCUAUGCGCAUCGCAUUCCAUUCCUC<br>GUCAAACUUAAUCACAACGAGACGCUAAGUUACCCGAACA<br>CCUACGAUCAAACGCUGUAUGCCAGCGUGGAGCAGGCCU<br>UCAACAUGGGCGCGGUGGCGGUUGGUGCGACUAUC**UAUU**<br>UUGGUUCGGAAGAGUCACGUCGCCAGAUUGAAGAAAUUU<br>CUGCGGCUUUUGAACGUGCGCACGAGCUGGGCAUGGUGA<br>CAGUGCUGUGGGCC**UAUU**UGCGUAACUCCGCCUUUAAGA<br>AAGAUGGCGUUGAUUACCAUGUUUCCGCCGACCUGACCG<br>GUCAGGCAAACCAUCUGGCGGCGACCAUAGGUGCAGAUA<br>UCGUCAAACAAAAAAUGGCGGAAAAUAACGGCGGCUAUAA<br>AGCAAUUAAUUACGGUUAUACCGACGAUCGCGUGUACAG<br>CAAGUUAACCAGCGAAAACCCGAUUGAUCUGGUGCGUUA<br>UCAGUUAGCUAACUGCUAUAUGGGCCGGGCCGGGUUGAU<br>AAACUCCGGCGGUGCUGCAGGCGGUGAAACUGACCUCAG<br>CGAUGCAGUGCGUACUGCGGUUAUCAACAAACGCGCUGG<br>CGGAAUGGGGCUGAUUCUUGGACGUAAGGCGUUCAAGAA<br>AUCGAUGGCUGACGGCGUGAAACUGAUUAACGCCGUGCA<br>GGAUGUUUAUCUCGAUAGCAAAAUUACUAUCGCCUGA
glyceraldehyde-3-phosphate dehydrogenase	*gap*A	AUGACUGUUAAGAUUGGUAUCAACGGUUUCGGCCG**UAUU**<br>GGUCGUUUGGCCUUCCGUCGCAUCCUUGAGUUGAAGGAU<br>ACUGCGGACGACAUCGAAGUUGUUGCAAUUAACGAUUUG<br>ACUAACCCUGCUAUGUUGGCAUACUUGUUGAAGUAUGAC<br>UCAACGCAUGGUACUUUGCCAGUUGAUGUUUCAGCUGAC<br>GAAGACGGUAUCAUCGUUGAUGGUAAGAAGAUCCGUGUU<br>UACGCUGAGCGUAACGCGGCUGACUUGAAGUGGGUUGCA<br>AACGAUGGUGUUGAAAUCGUUUUGGAGUCUACUGGAUUC<br>UACACUUCAGCAGAAAAGUCACAAGCUCACUUGGAUGCAG<br>GUGCUAAGAAGGUUUUGAUUUCAGCUCCAGCUGGAAACA<br>UCCCAACGGUUGUCUACGGUGUUAACCAAGACACGUUGA<br>CUGCAGACGAUCACAUCGUAUCUGCUGGUUCAUGCACGA<br>CGCAAUCAUUGGCUCCAUUGGCAAACGCCUUGGACAAGG<br>AAUUUGGUAUCGAGAUCGGUUUGAUGACGACGGUUCACG<br>CCUUCACUUCAACGCAAAUGAUCUUGGACGGUCCUAAGG<br>GAUCAAAGUUCCGUUCAAACCGUACUGCUUCAGCAAACAC<br>GAUUCCUCACUCAACUGGUGCCGCUAAGGCUAUCGGUUU<br>GGUUGUUCCUUCUGUUGCUGGUAAGUUGGACGGUCACGC<br>ACAACGUGUUGCUGUUGUUGACGGUUCUGUUACUGAGUU<br>GACGACUGUUUUGUCAAAGACUGUUACGGCUGAAGAAGU<br>UAACGAAGCUGUUAAGAAGUACACGAACGACUCAUUCGGU<br>GUUAACGAAGACGAGAUCGUGUCAUCAGACAUCAUUGGC<br>GACACACACGGUGCUGUGUUUGACCCAACUUUGACUAAG<br>GUUAUCACUGUUGGUGAUAAGCAAUUGGUUCAAACUGCU<br>GCUUGGUACGACAACGAAUACGGUUUCACUUCAAACAUGA<br>UCCGUACUUUGUUGCACUUGGCAACUCUCUAA

phosphoglycerate kinase	*pgk*	AUGUCUGUAAUUAAGAUGACCGAUCUGGAUCUUGCUGGG<br>AAACGUG**UAUU**UAUCCGUGCGGAUCUGAACGUACCAGUA<br>AAAGACGGGAAAGUAACCAGCGACGCGCGUAUCCGUGCU<br>UCUCUGCCGACCAUCGAACUGGCCCUGAAACAAGGCGCA<br>AAAGUGAUGGUAACUUCCCACCUGGGUCGUCCUACCGAA<br>GGCGAGUACAACGAAGAAUUCUCUCUGCUGCCGGUUGUU<br>AACUACCUGAAAGACAAACUGUCUAACCCGGUUCGUCUGG<br>UUAAAGAUUACCUCGACGGCGUUGACGUUGCUGAAGGUG<br>AACUGGUUGUUCUGGAAAACGUUCGCUUCAACAAAGGCG<br>AGAAGAAAGACGACGAAACCCUGUCCAAAAAAUACGCUGC<br>ACUGUGUGACGUGUUCGUAAUGGACGCAUUCGGUACUGC<br>UCACCGCGCGCAGGCUUCUACUCACGGUAUCGGUAAAUU<br>CGCUGACGUUGCGUGCGCAGGCCCGCUGCUGGCAGCUG<br>AACUGGACGCGCUGGGUAAAGCACUGAAAGAACCUGCUC<br>GCCCGAUGGUGGCUAUCGUUGGUGGUUCUAAAGUAUCUA<br>CCAAACUGACCGUUCUGGACUCCCUGUCUAAAAUCGCUG<br>ACCAGCUGAUUGUUGGUGGUGGUAUCGCUAACACCUUUA<br>UCGCGGCACAAGGCCACGAUGUGGGUAAAUCCCUGUACG<br>AAGCUGACCUGGUUGACGAAGCUAAACGUCUGCUGACCA<br>CCUGCAACAUCCCGGUUCCGUCUGAUGUUCGCGUAGCAA<br>CCGAGUUCUCUGAAACUGCACCGGCUACCCUGAAAUCUG<br>UUAACGAUGUGAAAGCUGACGAGCAGAUCCUGGAUAUCG<br>GUGAUGCUUCCGCUCAGGAACUGGCUGAAAUCCUGAAGA<br>AUGCGAAAACCAUUCUGUGGAACGGUCCGGUUGGCGUGU<br>UCGAAUUCCCGAACUUCCGCAAAGGUACUGAAAUCGUGG<br>CUAACGCUAUCGCAGACAGCGAAGCGUUCUCCAUCGCUG<br>GCGGCGGCGACACUCUGGCAGCAAUCGACCUGUUCGGCA<br>UUGCUGACAAAAUCUCCUACAUCUCCACUGGCGGCGGCG<br>CAUUCCUCGAAUUCGUGGAAGGUAAAGUACUGCCUGCAG<br>UAG
phosphoglycerate mutase	*gpm*A	AUGGCAAAGUUGGUUUUGAUCCGUCACGGUCAAAGUGAA<br>UGGAACGCAUUGAACUUGUUCAAUGGUUGGGUAGACACG<br>AAGUUGAGCGACAAGGG**UAUU**GCCCAAGCUCGUACUGCA<br>GGUGAAUUGUUGGCUAAGGAAGGCAUCCAAUUCGACCAA<br>GCAUACACGUCAGUUUUGACGCGUGCGAUCACGACAUUG<br>CACUACGCUUUGGAAGAAGCUGGUCAAUUGUGGAUUCCU<br>GAAAUGAAGUCAUGGCGUUUGAACGAGCGUCACUACGGU<br>GCUUUGCAAGGUUUGAACAAGGCCGACGCCGCUGAAAAG<br>UGGGGCGACGAGCAAGUUUUGCAAUGGCGUCGUUCAUAC<br>GAUGUUUUGCCUCCAUUGCUAGAAGAGCAAACGGAGACU<br>GUUGAAGUUUUGGGUAAGGAAUACCCAGCCUUCGACCGU<br>CGUUACGCUGAUGUACCUGAAGGUGAAUUGCCAUUCGGC<br>GAAAACUUGAAGGUUACUUUGGAACGUG**UAUU**GCCAUUC<br>UGGGAGUCAAACAUCUCAAAGGACUUGGCAGCAGGUAAG<br>AACGUUGUGAUUGCCGCCCACGGUAACUCAUUGCGUGCC<br>UUGGUAAAGCACAUCGAAGGUAUCUCAGAUGAUGAUAUCU<br>UGGGUGUUGAAAUCGCUAACGGUGAGCCUUUGGUAUACG<br>ACUUGGCAGAUGAUUUGUCAGUUGUUUCAAAGAAGGUUU<br>UGAAGCCAGAAGCUUAA
enolase	*eno*	AUGUCUGCAAUUACUGAUAUUUACGCACGCGAAGUCUUG<br>GACUCACGUGGUAACCCAACUGUUGAAGUUGAAGUUUAC<br>ACUGAAUUGGGUGGUUUCGGUCGCGGAAUCGUUCCUUCA<br>GGAGCUUCAACUGGUGUUAACGAAGCCGUUGAAUUGCGU<br>GACGGUGACAAGGGUCGUUUCUUGGGUAAGGGAACGUUG

		AAGGCCGUUGAAAACGUUAACAAGGUUAUCAAGGACAAGU<br>UGGUUGGUAUGGAUGUUACUGACCAAGUUUUGUUGGACC<br>GUACGAUGAUCGAAUUGGACGGAACUCCAAACAAGGGUAA<br>GUUGGGUGCCAACGCUAUCCUUGGUGUUUCAAUUGCAGC<br>UGCUCGUGCAGCUGCUGACGAAUUGGGAACGCCUUUGUA<br>CAACUACCUUGGUGGUUUCAACUCAAAGGUUUUGCCUAC<br>GCCAAUGAUGAACGUUGUUAACGGUGGAGCCCACGCGGA<br>CAACUCUGUUGACUUCCAAGAGUUCAUGAUCAUGCCAGU<br>UGGUGCAAAGACUGUUGCUGAAGCUAUCCGUAUGGGUUC<br>AGAAACUUUCCAUGCAUUGCAAGCAUUGUUGAAGGAAUCA<br>GGUCACUCAACUGCUGUUGGUGACGAAGGUGGAUUCGCU<br>CCUAACUUCGCCUCAAACGCAGAGCCAUUUGAGUUCUUG<br>UUGAACGCUAUCGAGCGUGCUGGUUACAAGCCUGGUAAG<br>GACAUCGCCAUUGCC
pyruvate kinase	*pyk*A	AUGAAGAAGACCAAGAUUGUUUCAACGCUUGGACCAGCUA<br>GUUCUGACGUCGAGAC**<u>UAUU</u>**ACGAAGUUAAUCGAGUCAG<br>GUGCCAACGUUGUACGUUUCAACUUCUCACACGGAGACC<br>ACGAGGAACACUUGGGUCGUAUGAACGCUGUGCGCGAAG<br>CCGAGAAGAUUUCAGGUAAGACUGUAGGAUUCUUGUUGG<br>ACACUAAGGGUGCUGAAAUCCGUACGACUGUUCAAGAGC<br>CAGGUAAGAUUGAGUUCAACAUCGGUGAUGUUGUUCGCA<br>UCUCAAUGGAUUCAUCUCUUGAAGGUACUAAGGAAAAGGU<br>UGCCGUAACUUACCCAGGUUUGUUCGACGAUGUUAAGGU<br>UGGCGGACACGUGUUGUUCGACGACGGUAAGAUUGACAU<br>GUUGAUCACUGAAAAGGACGAAGCUAACCGCGAAUUGGU<br>UACUGAAGUUCAAAACCACGGUUUGUUGGGAUCACGUAA<br>GGGUGUUAACGCACCUGGUGUUUCAAUCAACUUGCCAGG<br>UAUCACUGAAAAGGAUGCCGAUGACAUCCGCUUCGGUUU<br>GGACCAAGGUAUCCAAUACAUCGCCGCUUCAUUCGUACG<br>UAAGCCUUCAGACGUUGAAGACAUCCGUGCUUUGUUGGU<br>AGAAGCUGGUAAGGAAGAUGUUAUGAUCUUCCCUAAGAU<br>CGAAUCUCAAGAAGGUAUCGACAACUUUGCCGAAAUCUUG<br>AAGGUUUCUGAUGGU

Table S5 The mRNA of metabolic enzymes involved in pyruvate dehydrogenase system

Pyruvate dehydrogenase system		
Enzyme	Gene	Sequence (mRNA)
pyruvate dehydrogenase	*ace*E	AUGUCAGAACGUUUCCCAAAUGACGUGGAUCCGAUCGAAA<br>CUCGCGACUGGCUCCAGGCGAUCGAAUCGGUCAUCCGUG<br>AAGAAGGUGUUGAGCGUGCUCAGUAUCUGAUCGACCAAC<br>UGCUUGCUGAAGCCCGCAAAGGCGGUGUAAACGUAGCCG<br>CAGGCACAGGUAUCAGCAACUACAUCAACACCAUCCCCGU<br>UGAAGAACAACCGGAGUAUCCGGGUAAUCUGGAACUGGA<br>ACGCCG**UAUU**CGUUCAGCUAUCCGCUGGAACGCCAUCAU<br>GACGGUGCUGCGUGCGUCGAAAAAAGACCUCGAACUGGG<br>CGGCCAUAUGGCGUCCUUCCAGUCUUCCGCAACCAUUUA<br>UGAUGUGUGCUUUAACCACUUCUUCCGUGCACGCAACGA<br>GCAGGAUGGCGGCGACCUGGUUUACUUCCAGGGCCACAU<br>CUCCCCGGGCGUGUACGCUCGUGCUUUCCUGGAAGGUC<br>GUCUGACUCAGGAGCAGCUGGAUAACUUCCGUCAGGAAG<br>UUCACGGCAAUGGCCUCUCUUCCUAUCCGCACCCGAAAC<br>UGAUGCCGGAAUUCUGGCAGUUCCCGACCGUAUCUAUGG<br>GUCUGGGGCCGAUUGGUGC**UAUU**UACCAGGCUAAAUUCC<br>UGAAAUAUCUGGAACACCGUGGCCUGAAAGAUACCUCUAA<br>ACAGACCGUUUACGCGUUCCUCGGCGACGGUGAAAUGGA<br>CGAACCGGAAUCCAAAGGUGCGAUCACCAUCGCUACCCG<br>UGAAAAACUGGAUAACCUGGUCUUCGUUAUCAACUGUAAC<br>CUGCAGCGUCUUGACGGCCCGGUCACCGGUAACGGCAAG<br>AUCAUCAACGAACUGGAAGGCAUCUUCGAAGGUGCUGGC<br>UGGAACGUGAUCAAAGUGAUGUGGGGUAGCCGUUGGGAU<br>GAACUGCUGCGUAAAGAUACCAGCGGUAAACUGAUCCAG<br>CUGAUGAACGAAACCGUUGACGGCGACUACCAGACCUUC<br>AAAUCGAAAGAUGGUGCGUACGUUCGUGAACACUUCUUC<br>GGUAAAUAUCCUGAAACCGCAGCACUGGUUGCAGACUGG<br>ACUGACGAGCAGAUCUGGGCACUGAACCGUGGUGGUCAC<br>GAUCCGAAGAAAAUCUACGCUGCAUUCAAGAAAGCGCAGG<br>AAACCAAAGGCAAAGCGACAGUAAUCCUUGCUCAUACCAU<br>UAAAGGUUACGGCAUGGGCGACGCGGCUGAAGGUAAAAA<br>CAUCGCGCACCAGGUUAAGAAAAUGAACAUGGACGGCGU<br>GCGUCACAUCCGCGACCGUUUCAAUGUGCCGGUGUCUGA<br>UACAGAUAUCGAAAAACUGCCGUACAUCACCUUCCCGGAA<br>GGUUCUGAAGAGCAUACCUAUCUGCACGCUCAGCGUCAG<br>AAACUGCACGGUUAUCUGCCAAGCCGUCAGCCGAACUUC<br>ACCGAGAAGCUUGAGCUGCCGAGCCUGCAAGACUUCGGC<br>GCGCUGUUGGAAGAGCAGAGCAAAGAGAUCUCUACCACU<br>AUCGCUUUCGUUCGUGCUCUGAACGUGAUGCUGAAGAAC<br>AAGUCGAUCAAAGAUCGUCUGGUACCGAUCAUCGCCGAC<br>GAAGCGCGUACUUUCGGUAUGGAAGGUCUGUUCCGUCAG<br>AUUGG**UAUU**UACAGCCCGAACGGUCAGCAGUACACCCCG<br>CAGGACCGCGAGCAGGUUGCUUACUAUAAAGAAGACGAG<br>AAAGGUCAGAUUCUGCAGGAAGGGAUCAACGAGCUGGGC<br>GCAGGUUGUUCCUGGCUGGCAGCGGCGACCUCUUACAGC<br>ACCAACAAUCUGCCGAUGAUCCCGUUCUACAUC**UAUU**ACU<br>CGAUGUUCGGCUUCCAGCG**UAUU**GGCGAUCUGUGCUGG<br>GCGGCUGGCGACCAGCAAGCGCGUGGCUUCCUGAUCGG<br>CGGUACUUCCGGUCGUACCACCCUGAACGGCGAAGGUCU<br>GCAGCACGAAGAUGGUCACAGCCACAUUCAGUCGCUGAC

		UAUCCCGAACUGUAUCUCUUACGACCCGGCUUACGCUUA<br>CGAAGUUGCUGUCAUCAUGCAUGACGGUCUGGAGCGUAU<br>GUACGGUGAAAAACAAGAGAACGUUUACUACUACAUCACU<br>ACGCUGAACGAAAACUACCACAUGCCGGCAAUGCCGGAAG<br>GUGCUGAGGAAGGUAUCCGUAAAGGUAUCUACAAACUCG<br>AAAC**UAUU**GAAGGUAGCAAAGGUAAAGUUCAGCUGCUCG<br>GCUCCGGUUCUAUCCUGCGUCACGUCCGUGAAGCAGCUG<br>AGAUCCUGGCGAAAGAUUACGGCGUAGGUUCUGACGUUU<br>AUAGCGUGACCUCCUUCACCGAGCUGGCGCGUGAUGGUC<br>AGGAUUGUGAACGCUGGAACAUGCUGCACCCGCUGGAAA<br>CUCCGCGCGUUCCGUAUAUCGCUCAGGUGAUGAACGACG<br>CUCCGGCAGUGGCAUCUACCGACUAUAUGAAACUGUUCG<br>CUGAGCAGGUCCGUACUUACGUACCGGCUGACGACUACC<br>GCGUACUGGGUACUGAUGGCUUCGGUCGUUCCGACAGCC<br>GUGAGAACCUGCGUCACCACUUCGAAGUUGAUGCUUCUU<br>AUGUCGUGGUUGCGGCGCUGGGCGAACUGGCUAAACGU<br>GGCGAAAUCGAUAAGAAAGUGGUUGCUGACGCAAUCGCC<br>AAAUUCAACAUCGAUGCAGAUAAAGUUAACCCGCGUCUGG<br>CGUAA
dihydrolipoamide transacetylase	*ace*F	AUGGCUAUCGAAAUCAAAGUACCGGACAUCGGGGCUGAU<br>GAAGUUGAAAUCACCGAGAUCCUGGUCAAAGUGGGCGAC<br>AAAGUUGAAGCCGAACAGUCGCUGAUCACCGUAGAAGGC<br>GACAAAGCCUCUAUGGAAGUUCCGUCUCCGCAGGCGGGU<br>AUCGUUAAAGAGAUCAAAGUCUCUGUUGGCGAUAAAACCC<br>AGACCGGCGCACUGAUUAUGAUUUUCGAUUCCGCCGACG<br>GUGCAGCAGACGCUGCACCUGCUCAGGCAGAAGAGAAGA<br>AAGAAGCAGCUCCGGCAGCAGCACCAGCGGCUGCGGCGG<br>CAAAAGACGUUAACGUUCCGGAUAUCGGCAGCGACGAAG<br>UUGAAGUGACCGAAAUCCUGGUGAAAGUUGGCGAUAAAG<br>UUGAAGCUGAACAGUCGCUGAUCACCGUAGAAGGCGACA<br>AGGCUUCUAUGGAAGUUCCGGCUCCGUUUGCUGGCACCG<br>UGAAAGAGAUCAAAGUGAACGUGGGUGACAAAGUGUCUA<br>CCGGCUCGCUGAUUAUGGUCUUCGAAGUCGCGGGUGAAG<br>CAGGCGCGGCAGCUCCGGCCGCUAAACAGGAAGCAGCUC<br>CGGCAGCGGCCCCUGCACCAGCGGCUGGCGUGAAAGAAG<br>UUAACGUUCCGGAUAUCGGCGGUGACGAAGUUGAAGUGA<br>CUGAAGUGAUGGUGAAAGUGGGCGACAAAGUUGCCGCUG<br>AACAGUCACUGAUCACCGUAGAAGGCGACAAAGCUUCUAU<br>GGAAGUUCCGGCGCCGUUUGCAGGCGUCGUGAAGGAACU<br>GAAAGUCAACGUUGGCGAUAAAGUGAAAACUGGCUCGCU<br>GAUUAUGAUCUUCGAAGUUGAAGGCGCAGCGCCUGCGGC<br>AGCUCCUGCGAAACAGGAAGCGGCAGCGCCGGCACCGGC<br>AGCAAAAGCUGAAGCCCCGGCAGCAGCACCAGCUGCGAA<br>AGCGGAAGGCAAAUCUGAAUUUGCUGAAAACGACGCUUAU<br>GUUCACGCGACUCCGCUGAUCCGCCGUCUGGCACGCGAG<br>UUUGGUGUUAACCUUGCGAAAGUGAAGGGCACUGGCCGU<br>AAAGGUCGUAUCCUGCGCGAAGACGUUCAGGCUUACGUG<br>AAAGAAGCUAUCAAACGUGCAGAAGCAGCUCCGGCAGCGA<br>CUGGCGGUGGUAUCCCUGGCAUGCUGCCGUGGCCGAAG<br>GUGGACUUCAGCAAGUUUGGUGAAAUCGAAGAAGUGGAA<br>CUGGGCCGCAUCCAGAAAAUCUCUGGUGCGAACCUGAGC<br>CGUAACUGGGUAAUGAUCCCGCAUGUUACUCACUUCGAC<br>AAAACCGAUAUCACCGAGUUGGAAGCGUUCCGUAAACAGC<br>AGAACGAAGAAGCGGCGAAACGUAAGCUGGAUGUGAAGA<br>UCACCCCGGUUGUCUUCAUCAUGAAAGCCGUUGCUGCAG

		CUCUUGAGCAGAUGCCUCGCUUCAAUAGUUCGCUGUCGG<br>AAGACGGUCAGCGUCUGACCCUGAAGAAAUACAUCAACAU<br>CGGUGUGGCGGUGGAUACCCCGAACGGUCUGGUUGUUC<br>CGG**UAUU**CAAAGACGUCAACAAGAAAGGCAUCAUCGAGCU<br>GUCUCGCGAGCUGAUGAC**UAUU**UCUAAGAAAGCGCGUGA<br>CGGUAAGCUGACUGCGGGCGAAAUGCAGGGCGGUUGCUU<br>CACCAUCUCCAGCAUCGGCGGCCUGGGUACUACCCACUU<br>CGCGCCGAUUGUGAACGCGCCGGAAGUGGCUAUCCUCGG<br>CGUUUCCAAGUCCGCGAUGGAGCCGGUGUGGAAUGGUAA<br>AGAGUUCGUGCCGCGUCUGAUGCUGCCGAUUUCUCUCUC<br>CUUCGACCACCGCGUGAUCGACGGUGCUGAUGGUGCCCG<br>UUUCAUUACCAUCAUUAACAACACGCUGUCUGACAUUCGC<br>CGUCUGGUGAUGUAA
dihydrolipoamide dehydrogenase	*lpd*	AUGAGUACUGAAAUCAAAACUCAGGUCGUGGUACUUGGG<br>GCAGGCCCCGCAGGUUACUCCGCUGCCUUCCGUUGCGCU<br>GAUUUAGGUCUGGAAACCGUAAUCGUAGAACGUUACAACA<br>CCCUUGGCGGUGUUUGCCUGAACGUCGGCUGUAUCCCUU<br>CUAAAGCACUGCUGCACGUAGCAAAAGUUAUCGAAGAAGC<br>CAAAGCGCUGGCUGAACACGGUAUCGUCUUCGGCGAACC<br>GAAAACCGAUAUCGACAAGAUUCGUACCUGGAAAGAGAAA<br>GUGAUCAAUCAGCUGACCGGUGGUCUGGCUGGUAUGGCG<br>AAAGGCCGCAAAGUCAAAGUGGUCAACGGUCUGGGUAAA<br>UUCACCGGGGCUAACACCCUGGAAGUUGAAGGUGAGAAC<br>GGCAAAACCGUGAUCAACUUCGACAACGCGAUCAUUGCAG<br>CGGGUUCUCGCCCGAUCCAACUGCCGUU**UAUU**CCGCAUG<br>AAGAUCCGCGUAUCUGGGACUCCACUGACGCGCUGGAAC<br>UGAAAGAAGUACCAGAACGCCUGCUGGUAAUGGGUGGCG<br>GUAUCAUCGGUCUGGAAAUGGGCACCGUUUACCACGCGC<br>UGGGUUCACAGAUUGACGUGGUUGAAAUGUUCGACCAGG<br>UUAUCCCGGCAGCUGACAAAGACAUCGUUAAAGUCUUCAC<br>CAAGCGUAUCAGCAAGAAAUUCAACCUGAUGCUGGAAACC<br>AAAGUUACCGCCGUUGAAGCGAAAGAAGACGGCAUUUAU<br>GUGACGAUGGAAGGCAAAAAAGCACCCGCUGAACCGCAG<br>CGUUACGACGCCGUGCUGGUAGCGAUUGGUCGUGUGCC<br>GAACGGUAAAAACCUCGACGCAGGCAAAGCAGGCGUGGA<br>AGUUGACGACCGUGGUUUCAUCCGCGUUGACAAACAGCU<br>GCGUACCAACGUACCGCACAUCUUUGCUAUCGGCGAUAU<br>CGUCGGUCAACCGAUGCUGGCACACAAAGGUGUUCACGA<br>AGGUCACGUUGCCGCUGAAGUUAUCGCCGGUAAGAAACA<br>CUACUUCGAUCCGAAAGUUAUCCCGUCCAUCGCCUAUACC<br>GAACCAGAAGUUGCAUGGGUGGGUCUGACUGAGAAAGAA<br>GCGAAAGAGAAAGGCAUCAGCUAUGAAACCGCCACCUUCC<br>CGUGGGCUGCUUCUGGUCGUGCUAUCGCUUCCGACUGC<br>GCAGACGGUAUGACCAAGCUGAUUUUCGACAAAGAAUCUC<br>ACCGUGUGAUCGGUGGUGCGAUUGUCGGUACUAACGGCG<br>GCGAGCUGCUGGGUGAAAUCGGCCUGGCAAUCGAAAUGG<br>GUUGUGAUGCUGAAGACAUCGCACUGACCAUCCACGCGC<br>ACCCGACUCUGCACGAGUCUGUGGGCCUGGCGGCAGAAG<br>UGUUCGAAGGUAGCAUUACCGACCUGCCGAACCCGAAAG<br>CGAAGAAGAAGUAA

TABLE S6 The mRNA of metabolic enzymes involved in citric acid cycle (TCA cycle)

Citric acid cycle (TCA cycle)		
Enzyme	Gene	Sequence (mRNA)
citrate synthase	*glt*A	AUGGCUGAUACAAAAGCAAAACUCACCCUCAACGGGGACA<br>CAGCUGUUGAACUGGAUGUGCUGAAAGGCACGCUGGGUC<br>AAGAUGU**UAUU**GAUAUCCGUACUCUCGGUUCAAAAGGUG<br>UGUUCACCUUUGACCCUGGCUUCACUUCAACCGCAUCCU<br>GCGAAUCUAAAAUUACUUU**UAUU**GAUGGUGAUGAAGG**UA**<br>**UU**UUGCUGCACCGCGGUUUCCCGAUCGAUCAGCUGGCGA<br>CCGAUUCUAACUACCUGGAAGUUUGUUACAUCCUGCUGA<br>AUGGUGAAAAACCGACUCAGGAACAGUAUGACGAAUUUAA<br>AACUACGGUGACCCGUCAUACCAUGAUCCACGAGCAGAUU<br>ACCCGUCUGUUCCACGCUUUCCGUCGCGACUCACAUCCA<br>AUGGCAGUCAUGUGUGG**UAUU**ACCGGCGCGCUGGCGGC<br>GUUCUAUCACGACUCGCUGGAUGUUAACAAUCCUCGUCA<br>UCGUGAAAUUGCCGCGUUCCGCCUGCUGUCGAAAAUGCC<br>GACCAUGGCAGCGAUGUGUUACAAG**UAUU**CCAUUGGUCA<br>GCCAUUUGUUUACCCGCGCAACGAUCUCUCCUACGCCGG<br>UAACUUCCUGAAUAUGAUGUUCUCUACGCCGUGCGAACC<br>GUAUGAAGUUAAUCCGAUUCUGGAACGUGCUAUGGACCG<br>**UAUU**UUGAUCCUGCACGCUGACCAUGAACAGAACGCCUC<br>CACCUCCACCGUGCGUACCGCUGGCUCUUCGGGUGCGAA<br>CCCGUUUGCCUGUAUCGCAGCAGG**UAUU**GCUUCACUGUG<br>GGGACCUGCGCACGGUGGUGCUAACGAAGCAGCGCUGAA<br>AAUGCUGGAAGAAAUCAGCUCCGUUAAACACAUUCCGGAA<br>UUUGUUCGUCGUGCGAAAGAUAAAAAUGAUUCUUUCCGC<br>CUGAUGGGCUUCGGUCACCGCGUGUACAAAAAUUACGAC<br>CCGCGCGCCACCGUAAUGCGUGAAACCUGCCAUGAAGUG<br>CUGAAAGAGCUGGGCACGAAGGAUGACCUGCUGGAAGUG<br>GCUAUGGAGCUGGAAAACAUCGCGCUGAACGACCCGUAC<br>UUUAUCGAGAAGAAACUGUACCCGAACGUCGAUUUCUACU<br>CUGGUAUCAUCCUGAAAGCGAUGGG**UAUU**CCGUCUUCCA<br>UGUUCACCGUCAUUUUCGCAAUGGCACGUACCGUUGGCU<br>GGAUCGCCCACUGGAGCGAGAUGCACAGUGACGGUAUGA<br>AGAUUGCCCGUCCGCGUCAGCUGUAUACAGGAUAUGAAA<br>AACGCGACUUUAAAAGCGAUAUCAAGCGUUAA
aconitase	*acn*A	AUGAGCAACCCAUUCGCACACCUUGCUGAGCCAUUGGAU<br>CCUGUACAACCAGGAAAGAAAUUCUUCAAUUUGAAUAAAU<br>UGGAGGAUUCAAGAUAUGGGCGCUUACCAUUUUCGAUCA<br>GAGUUCUUCUGGAAGCAGCCAUUCGGAAUUGUGAUGAGU<br>UUUUGGUGAAGAAACAGGA**UAUU**GAAAA**UAUU**CUACAUUG<br>GAAUGUCACGCAGCACAAGAACAUAGAAGUGCCAUUUAAG<br>CCUGCUCGUGUCAUCCUGCAGGACUUUACGGGUGUGCCC<br>GCUGUGGUUGACUUUGCUGCAAUGCGUGAUGCUGUGAAA<br>AAGUUAGGAGGAGAUCCAGAGAAAAUAAACCCUGUCUGCC<br>CUGCUGAUCUUGUAAUAGAUCAUUCCAUCCAGGUUGAUU<br>UCAACAGAAGGGCAGACAGUUUACAGAAGAAUCAAGACCU<br>GGAAUUUGAAAGAAAUAGAGAGCGAUUUGAAUUUUUAAAG<br>UGGGGUUCCCAGGCUUUUCACAACAUGCGGAU**UAUU**CCC<br>CCUGGCUCAGGAAUCAUCCACCAGGUGAAUUUGGAA**UAU**<br>**U**UGGCAAGAGUGG**UAUU**UGAUCAGGAUGGA**UAUU**AUUAC<br>CCAGACAGCCUCGUGGGCACAGACUCGCACACUACCAUG<br>AUUGAUGGCUUGGGCAUUCUUGGUUGGGGUGUCGGUGG<br>**UAUU**GAAGCAGAAGCUGUCAUGCUGGGUCAGCCAAUCAG

		UAUGGUGCUUCCUCAGGUGAUUGGCUACAGGCUGAUGGG<br>GAAGCCCCACCCUCUGGUAACAUCCACUGACAUCGUGCU<br>CACCAUUACCAAGCACCUCCGCCAGGUUGGGGUAGUGGG<br>CAAAUUUGUCGAGUUCUUCGGGCCUGGAGUAGCCCAGUU<br>GUCCAUUGCUGACCGAGCUACGAUUGCUAACAUGUGUCC<br>AGAGUACGGAGCAACUGCUGCCUUUUUCCCAGUUGAUGA<br>AGUUAGUAUCACGUACCUGGUGCAAACAGGUCGUGAUGA<br>AGAAAAAUUAAAGUA**UAUU**AAAAAAUAUCUUCAGGCUGUA<br>GGAAUGUUUCGAGAUUUCAAUGACCCUUCUCAAGACCCA<br>GACUUCACCCAGGUUGUGGAAUUAGAUUUGAAAACAGUA<br>GUGCCUUGCUGUAGUGGACCCAAAAGGCCUCAGGACAAA<br>GUUGCUGUGUCCGACAUGAAAAAGGACUUUGAGAGCUGC<br>CUUGGAGCCAAGCAAGGAUUUAAAGGAUUCCAAGUUGCU<br>CCUGAACAUCAUAAUGACCAUAAGACCUUUAUCUAUGAUA<br>ACACUGAAUUCACCCUUGCUCAUGGUUCUGUGGUCAUUG<br>CUGCCAUUACUAGCUGCACAAACACCAGUAAUCCGUCUGU<br>GAUGUUAGGGGCAGGAUUGUUAGCAAAGAAAGCUGUGGA<br>UGCUGGCCUGAACGUGAUGCCUUACAUCAAAACUAGCCU<br>GUCUCCUGGGAGUGGCGUGGUCACCUACUACCUACAAGA<br>AAGCGGAGUCAUGCCUUAUCUGUCUCAGCUUGGGUUUGA<br>CGUGGUGGGCUAUGGCUGCAUGACCUGCAUUGGCAACAG<br>UGGGCCUUUACCUGAACCUGUGGUAGAAGCCAUCACACA<br>GGGAGACCUUGUAGCUGUUGGAGUACUAUCUGGAAACAG<br>GAAUUUUGAAGGUCGAGUUCACCCCAACACCCGGGCCAA<br>C**UAUU**UAGCCUCUCCCCCCUUAGUAAUAGCAUAUGCAAUU<br>GCUGGAACCAUCAGAAUCGACUUUGAGAAAGAGCCAUUG<br>GGAGUAAAUGCAAAGGGACAGCAGG**UAUU**UCUGAAAGAU<br>AUCUGGCCGACUAGAGACGAGAUCCAGGCAGUGGAGCGU<br>CAGUAUGUCAUCCCGGGGAUGUUUAAGGAAGUCUAUCAG<br>AAAAUAGAGACUGUGAAU
	*acn*B	GUGCUAGAAGAAUACCGUAAGCACGUAGCUGAGCGUGCC<br>GCUGAGGGGAUUGCGCCCAAACCCCUGGAUGCAAACCAA<br>AUGGCCGCACUUGUAGAGCUGCUGAAAAACCCGCCCGCG<br>GGCGAAGAAGAAUUCCUGUUAGAUCUGUUAACCAACCGU<br>GUUCCCCCAGGCGUCGAUGAAGCCGCCUAUGUCAAAGCA<br>GGCUUCCUGGCUGCUAUCGCUAAAGGCGAAGCCAAAUCC<br>CCUCUGCUGACUCCGGAAAAAGCCAUCGAACUGCUGGGC<br>ACCAUGCAGGGUGGUUACAACAUUCAUCCGCUGAUCGAC<br>GCGCUGGAUGAUGCCAAACUGGCACCGAUCGCUGCCAAA<br>GCACUUUCUCACACACUGCUGAUGUUCGAUAACUUCUAU<br>GACGUAGAAGAGAAAGCGAAAGCAGGCAACGAAUAUGCGA<br>AGCAGGUAAUGCAGUCCUGGGCGGAUGCCGAAUGGUUCC<br>UGAAUCGCCCGGCGCUGGCUGAAAAACUGACCGUUACCG<br>UCUUCAAAGUCACUGGCGAAACCAACACCGAUGACCUCUC<br>UCCGGCACCGGAUGCGUGGUCACGCCCGGAUAUCCCACU<br>GCACGCGCUGGCGAUGCUGAAAAACGCCCGUGAAGGCAU<br>CGAGCCAGACCAGCCAGGUGUUGUUGGUCCGAUCAAGCA<br>AAUCGAAGCUCUGCAACAGAAAGGUUUCCCGCUGGCGUA<br>CGUCGGUGACGUUGUGGGUACGGGUUCAUCGCGUAAAUC<br>CGCCACGAACUCCGUACUGUGGUUUAUGGGUGAUGA**UAU**<br>**U**CCACAUGUGCCGAACAAACGCGGCGGUGGUUUGUGCCU<br>CGGCGGUAAAAUUGCACCAAUCUUCUUUAACACAAUGGAA<br>GACGCAGGUGCACUGCCAAUCGAAGUCGACGUCUCUAAC<br>CUGAACAUGGGCGACGUAAUUGACGUUUACCCGUACAAA

		GGUGAAGUGCGUAACCACGAAACCGGCGAACUGCUGGCG<br>ACCUUCGAACUGAAAACCGACGUGCUGAUUGAUGAAGUG<br>CGUGCUGGCGGCCG**UAUU**CCGCUGAU**UAUU**GGGCGUGG<br>CCUGACCACCAAAGCGCGUGAAGCACUUGGUCUGCCGCA<br>CAGUGAUGUGUUCCGUCAGGCGAAAGAUGUCGCUGAGAG<br>CGAUCGCGGCUUCUCGCUGGCGCAAAAAAUGGUAGGCCG<br>UGCCUGUGGCGUGAAAGGCAUUCGUCCGGGCGCGUACU<br>GCGAACCGAAAAUGACUUCUGUAGGCUCUCAGGACACCA<br>CCGGCCCGAUGACCCGUGAUGAACUGAAAGACCUGGCGU<br>GCCUGGGCUUCUCGGCUGACCUGGUGAUGCAGUCUUUCU<br>GCCACACCGCGGCGUAUCCGAAGCCAGUUGACGUGAACA<br>CGCACCACACGCUGCCGGACUUCAUUAUGAACCGUGGCG<br>GUGUGUCGCUGCGUCCGGGUGACGGCGUCAUUCACUCC<br>UGGCUGAACCGUAUGCUGCUGCCGGAUACCGUCGGUACC<br>GGUGGUGACUCCCAUACCCGUUUCCCGAUCGGUAUCUCU<br>UUCCCGGCGGGUUCUGGUCUGGUGGCGUUUGCUGCCGC<br>AACUGGCGUAAUGCCGCUGGAUAUGCCGGAAUCCGUUCU<br>GGUGCGCUUCAAAGGCAAAAUGCAGCCGGGCAUCACCCU<br>GCGCGAUCUGGUACACGCGAUCCCGCUGUAUGCGAUCAA<br>ACAAGGUCUGCUGACCGUUGAGAAGAAAGGCAAGAAAAAC<br>AUCUUCUCUGGCCGCAUCCUGGAAAUUGAAGGUCUGCCG<br>GAUCUGAAAGUUGAGCAGGCCUUUGAGCUAACCGAUGCG<br>UCCGCCGAGCGUUCUGCCGCUGGUUGUACCAUCAAGCUG<br>AACAAAGAACCGAUCAUCGAAUACCUGAACUCUAACAUCG<br>UCCUGCUGAAGUGGAUGAUCGCGGAAGGUUACGGCGAUC<br>GUCGUACCCUGGAACGUCG**UAUU**CAGGGCAUGGAAAAAU<br>GGCUGGCGAAUCCUGAGCUGCUGGAAGCCGAUGCAGAUG<br>CGGAAUACGCGGCAGUGAUCGACAUCGAUCUGGCGGA**UA**<br>**UU**AAAGAGCCAAUCCUCUGUGCACCGAACGACCCGGACG<br>ACGCACGUCCGCUGUCUGCGGUACAGGGCGAGAAGAUCG<br>ACGAAGUGUUUAUCGGUUCCUGCAUGACCAACAUCGGUC<br>ACUUCCGUGCUGCGGGUAAACUGCUGGAUGCGCACAAAG<br>GCCAGUUGCCGACGCGCCUGUGGGUGGCACCGCCAACCC<br>GUAUGGAUGCCGCGCAGUUGACCGAAGAAGGCUACUAUA<br>GCGUCUUCGGUAAGAGCGGUGCGCGUAUCGAGAUCCCUG<br>GCUGUUCCCUGUGUAUGGGUAACCAGGCGCGUGUAGCAG<br>ACGGUGCGACGGUGGUUUCCACCUCUACCCGUAACUUCC<br>CGAACCGUCUGGGGACUGGCGCGAAUGUCUUCCUGGCUU<br>CUGCGGAACUGGCGGCUGUUGCGGCGCUGAUUGGCAAAC<br>UGCCGACGCCGGAAGAGUACCAGACCUACGUGGCGCAAG<br>UAGAUAAAACUGCCGUUGAUACUUAUCGUUAUCUGAACUU<br>CAACCAGCUUUCUCAGUACACCGAAAAAGCCGAUGGGGU<br>GAUUUUCCAGACUGCGGUUUAA
isocitrate dehydrogenase	*icd*	AUGGAAAGUAAAGUAGUUGUUCCGGCACAAGGCAAGAAG<br>AUCACCCUGCAAAACGGCAAACUCAACGUUCCUGAAAAUC<br>CGAUUAUCCCUUACAUUGAAGGUGAUGGAAUCGGUGUAG<br>AUGUAACCCCAGCCAUGCUGAAAGUGGUCGACGCUGCAG<br>UCGAGAAAGCCUAUAAAGGCGAGCGUAAAAUCUCCUGGAU<br>GGAAAUUUACACCGGUGAAAAAUCCACACAGGUUUAUGGU<br>CAGGACGUCUGGCUGCCUGCUGAAACUCUUGAUCUGAUU<br>CGUGAAUAUCGCGUUGCCAUUAAAGGUCCGCUGACCACU<br>CCUGUUGGUGGCGG**UAUU**CGCUCUCUGAACGUUGCCCU<br>GCGCCAGGAACUGGAUCUCUACAUCUGCCUGCGUCCGGU<br>ACGUUACUAUCAGGGCACUCCAAGCCCGGUUAAACACCCU<br>GAACUGACCGAUAUGGUUAUCUUCCGUGAAAACUCGGAA

		GACAUUUAUGCGGGUAUCGAAUGGAAAGCAGACUCUGCC<br>GACGCCGAGAAAGUGAUUAAAUUCCUGCGUGAAGAGAUG<br>GGGGUGAAGAAAAUUCGCUUCCCGGAACAUUGUGGUAUC<br>GG**UAUU**AAGCCGUGUUCGGAAGAAGGCACCAAACGUCUG<br>GUUCGUGCAGCGAUCGAAUACGCAAUUGCUAACGAUCGU<br>GACUCUGUGACUCUGGUGCACAAAGGCAACAUCAUGAAG<br>UUCACCGAAGGCGCGUUUAAAGACUGGGGCUACCAGCUG<br>GCGCGUGAAGAGUUUGGCGGUGAACUGAUCGACGGCGG<br>CCCGUGGCUGAAAGUUAAAAACCCGAACACCGGCAAAGAG<br>AUCGUCAUUAAAGACGUGAUUGCUGAUGCAUUCCUGCAA<br>CAGAUCCUGCUGCGUCCGGCUGAAUAUGAUGUUAUCGCC<br>UGUAUGAACCUGAACGGUGACUACAUUUCUGACGCUCUG<br>GCAGCGCAGGUUGGCGGUAUCGGUAUCGCCCCUGGAGCA<br>AACAUCGGUGACGAAUGCGCCCUGUUUGAAGCCACCCAC<br>GGUACUGCGCCGAAAUACGCCGGUCAGGACAAAGUAAAC<br>CCUGGCUC**UAUU**AUUCUCUCCGCUGAGAUGAUGUUACGC<br>CAUAUGGGCUGGACUGAAGCCGCAGACCUGAUUGUUAAA<br>GGUAUGGAAGGCGCAAUCAAUGCGAAGACCGUAACCUAU<br>GACUUCGAGCGUCUGAUGGAAGGCGCUAAACUGCUGAAA<br>UGUUCAGAAUUUGGUGAUGCGAUCAUCAAGAACAUGUAA
α-ketoglutarate dehydrogenase	*suc*A	AUGCAGAACAGCGCUUUGAAAGCCUGGUUGGACUCUUCU<br>UACCUCUCUGGCGCAAACCAGAGCUGGAUAGAACAGCUC<br>UAUGAAGACUUCUUAACCGAUCCUGACUCGGUUGACGCU<br>AACUGGCGUUCGACGUUCCAGCAGUUACCUGGUACGGGA<br>GUCAAACCGGAUCAAUUCCACUCUCAAACGCGUGAA**UAUU**<br>UCCGCCGCCUGGCGAAAGACGCUUCACGUUACUCUUCAA<br>CGAUCUCCGACCCUGACACCAAUGUGAAGCAGGUAAAAGU<br>CCUGCAGCUCAUUAACGCAUACCGCUUCCGUGGUCACCA<br>GCAUGCGAAUCUCGAUCCGCUGGGACUGUGGCAGCAAGA<br>UAAAGUGGCCGAUCUGGAUCCGUCUUUCCACGAUCUGAC<br>CGAAGCAGACUUCCAGGAGACCUUCAACGUCGGUUCAUU<br>UGCCAGCGGCAAAGAAACCAUGAAACUCGGCGAGCUGCU<br>GGAAGCCCUCAAGCAAACCUACUGCGGCCCGAUUGGUGC<br>CGAGUAUAUGCACAUCACCAGCACCGAAGAAAAACGCUGG<br>AUCCAACAGCGUAUCGAGUCUGGUCGCGCGACUUUCAAU<br>AGCGAAGAGAAAAAACGCUUCUUAAGCGAACUGACCGCCG<br>CUGAAGGCCUUGAACGUUACCUCGGCGCAAAAUUCCCUG<br>GCGCAAAACGCUUCUCGCUGGAAGGCGGUGACGCGUUAA<br>UCCCGAUGCUUAAAGAGAUGAUCCGCCACGCUGGCAACA<br>GCGGCACCCGCGAAGUGGUUCUCGGGAUGGCGCACCGU<br>GGUCGUCUGAACGUGCUGGUGAACGUGCUGGGUAAAAAA<br>CCGCAAGACUUGUUCGACGAGUUCGCCGGUAAACAUAAA<br>GAACACCUCGGCACGGGUGACGUGAAAUACCACAUGGGC<br>UUCUCGUCUGACUUCCAGACCGAUGGCGGCCUGGUGCAC<br>CUGGCGCUGGCGUUUAACCCGUCUCACCUUGAGAUUGUA<br>AGCCCGGUAGUUAUCGGUUCUGUUCGUGCCCGUCUGGAC<br>AGACUUGAUGAGCCGAGCAGCAACAAAGUGCUGCCAAUCA<br>CCAUCCACGGUGACGCCGCAGUGACCGGGCAGGGUGUG<br>GUUCAGGAAACUCUGAACAUGUCGAAAGCGCGUGGUUAU<br>GAAGUUGGCGGUACGGUACGUAUCGUUAUCAACAACCAG<br>GUUGGUUUCACCACCUCUAAUCCGCUGGAUGCUCGUUCU<br>ACACCGUACUGUACUGAUAUCGGUAAGAUGGUUCAGGCA<br>CCGAUUUUCCACGUUAACGCGGAUGAUCCGGAAGCCGUU<br>GCCUUUGUGACCCGUCUGGCGCUCGAUUUCCGUAACACC

		UUUAAACGUGAUGUCUUCAUCGACCUGGUGUGCUACCGC<br>CGUCACGGCCACAACGAAGCCGACGAGCCGAGCGCAACC<br>CAGCCGCUGAUGUAUCAGAAAUCAAAAAACAUCCGACGC<br>CGCGCAAAAUCUAUGCUGACAAGCUGGAGCAGGAAAAAGU<br>GGCGACGCUGGAAGAUGCCACCGAGAUGGUUAACCUGUA<br>CCGCGAUGCGCUGGAUGCUGGCGAUUGCGUAGUGGCAG<br>AGUGGCGUCCGAUGAACAUGCACUCUUUCACCUGGUCGC<br>CGUAUCUCAACCACGAAUGGGACGAAGAGUACCCGAACAA<br>AGUUGAGAUGAAGCGCCUGCAGGAGCUGGCGAAACGCAU<br>CAGCACGGUGCCGGAAGCAGUUGAAAUGCAGUCUCGCGU<br>UGCCAAGAUUUAUGGCGAUCGCCAGGCGAUGGCUGCCGG<br>UGAGAAACUGUUCGACUGGGGCGGUGCGGAAAACCUCGC<br>UUACGCCACGCUGGUUGACGAAGGCAUUCCGGUUCGCCU<br>GUCGGGUGAAGACUCCGGUCGCGGUACCUUCUUCCACCG<br>CCACGCGGUGAUCCACAACCAGUCUAACGGUUCCACUUA<br>CACGCCGUUGCAACACAUCCAUAACGGGCAGGGCGCGUU<br>CCGCGUCUGGGACUCCGUACUGUCUGAAGAAGCAGUGCU<br>GGCGUUUGAAUACGGUUAUGCCACCGCAGAACCACGCAC<br>UCUGACUAUCUGGGAAGCGCAGUUCGGUGACUUCGCCAA<br>CGGUGCGCAGGUGGUUAUCGACCAGUUCAUCUCUUCUGG<br>CGAACAGAAAUGGGGUCGGAUGUGUGGCCUGGUGAUGUU<br>GCUGCCGCACGGUUACGAAGGGCAGGGGCCGGAGCACUC<br>CUCCGCGCGUCUGGAACGUUAUCUGCAACUUUGUGCUGA<br>GCAAAACAUGCAGGUUUGCGUACCGUCUACCCCGGCACA<br>GGUUUACCACAUGCUGCGUCGUCAGGCGCUGCGCGGGAU<br>GCGUCGUCCGCUGGUCGUGAUGUCGCCGAAAUCCCUGCU<br>GCGUCAUCCGCUGGCGGUUUCCAGCCUCGAAGAACUGGC<br>GAACGGCACCUUCCUGCCAGCCAUCGGUGAAAUCGACGA<br>GCUUGAUCCGAAGGGCGUGAAGCGCGUAGUAAUGUGUUC<br>UGGUAAGGUU**UAUU**ACGACCUGCUGGAACAGCGUCGUAA<br>GAACAAUCAACACGAUGUCGCCAUUGUGCGUAUCGAGCAA<br>CUCUACCCGUUCCCGCAUAAAGCGAUGCAGGAAGUGUUG<br>CAGCAGUUUGCUCACGUCAAGGAUUUUGUCUGGUGCCAG<br>GAAGAGCCGCUCAACCAGGGCGCAUGGUACUGCAGCCAG<br>CAUCAUUUCCGUGAAGUGAUUCCGUUUGGGGCUUCUCUG<br>CGUUAUGCAGGCCGCCCGGCCUCCGCCUCUCCGGCGGUA<br>GGGUAUAUGUCCGUUCACCAGAAACAGCAACAAGAUCUG<br>GUUAAUGACGCGCUGAACGUCGAAUAA
	*suc*B	AUGAGUAGCGUAGA**UAUU**CUGGUCCCUGACCUGCCUGAA<br>UCCGUAGCCGAUGCCACCGUCGCAACCUGGCAUAAAAAAC<br>CCGGCGACGCAGUCGUACGUGAUGAAGUGCUGGUAGAAA<br>UCGAAACUGACAAAGUGGUACUGGAAGUACCGGCAUCAG<br>CAGACGGCAUUCUGGAUGCGGUUCUGGAAGAUGAAGGUA<br>CAACCGUAACGUCUCGUCAGAUCCUUGGUCGCCUGCGUG<br>AAGGCAACAGCGCCGGUAAAGAAACCAGCGCCAAAUCUGA<br>AGAGAAAGCGUCCACUCCGGCGCAACGCCAGCAGGCGUC<br>UCUGGAAGAGCAAAACAACGAUGCGUUAAGCCCGGCGAU<br>CCGUCGCCUGCUGGCUGAACACAAUCUCGACGCCAGCGC<br>CAUUAAAGGCACCGGUGUGGGUGGUCGUCUGACUCGUGA<br>AGAUGUGGAAAAACAUCUGGCGAAAGCCCCGGCGAAAGA<br>GUCUGCUCCGGCAGCGGCUGCUCCGGCGGCGCAACCGG<br>CUCUGGCUGCACGUAGUGAAAAACGUGUCCCGAUGACUC<br>GCCUGCGUAAGCGUGUGGCAGAGCGUCUGCUGGAAGCGA<br>AAAACUCCACCGCCAUGCUGACCACGUUCAACGAAGUCAA<br>CAUGAAGCCGAUUAUGGAUCUGCGUAAGCAGUACGGUGA

		AGCGUUUGAAAAACGCCACGGCAUCCGUCUGGGCUUUAU<br>GUCCUUCUACGUGAAAGCGGUGGUUGAAGCCCUGAAACG<br>UUACCCGGAAGUGAACGCGUCUAUCGACGGCGAUGACGU<br>GGUUUACCACAAC**UAUU**UCGACGUCAGCAUGGCGGUUUC<br>UACGCCGCGCGGCCUGGUGACACCGGUACUGCGUGAUGU<br>CGAUACCCUCGGCAUGGCAGACAUCGAGAAGAAAAUCAAA<br>GAGCUGGCAGUCAAAGGCCGUGACGGCAAGCUGACCGUU<br>GAAGAUCUGACCGGUGGUAACUUCACCAUCACCAACGGU<br>GGUGUGUUCGGUUCCCUGAUGUCUACGCCGAUCAUCAAC<br>CCGCCGCAGAGCGCAAUUCUGGGUAUGCACGCUAUCAAA<br>GAUCGUCCGAUGGCGGUGAAUGGUCAGGUUGAGAUCCUG<br>CCGAUGAUGUACCUGGCGCUGUCCUACGAUCACCGUCUG<br>AUCGAUGGUCGCGAAUCCGUGGGCUUCCUGGUAACGAUC<br>AAAGAGUUGCUGGAAGAUCCGACGCGUCUGCUGCUGGAC<br>GUGUAG
succinyl-CoA synthetase	*suc*C	AUGAACUUACAUGAAUAUCAGGCAAAACAACUUUUUGCCC<br>GCUAUGGCUUACCAGCACCGGUGGGUUAUGCCUGUACUA<br>CUCCGCGCGAAGCAGAAGAAGCCGCUUCAAAAAUCGGUG<br>CCGGUCCGUGGGUAGUGAAAUGUCAGGUUCACGCUGGUG<br>GCCGCGGUAAAGCGGGCGGUGUGAAAGUUGUAAACAGCA<br>AAGAAGACAUCCGUGCUUUUGCAGAAAACUGGCUGGGCA<br>AGCGUCUGGUAACGUAUCAAACAGAUGCCAAUGGCCAACC<br>GGUUAACCAGAUUCUGGUUGAAGCAGCGACCGAUAUCGC<br>UAAAGAGCUGUAUCUCGGUGCCGUUGUUGACCGUAGUUC<br>CCGUCGUGUGGUCUUUAUGGCCUCCACCGAAGGCGGCGU<br>GGAAAUCGAAAAAGUGGCGGAAGAAACUCCGCACCUGAUC<br>CAUAAAGUUGCGCUUGAUCCGCUGACUGGCCCGAUGCCG<br>UAUCAGGGACGCGAGCUGGCGUUCAAACUGGGUCUGGAA<br>GGUAAACUGGUUCAGCAGUUCACCAAAAUCUUCAUGGGC<br>CUGGCGACCAUUUUCCUGGAGCGCGACCUGGCGUUGAUC<br>GAAAUCAACCCGCUGGUCAUCACCAAACAGGGCGAUCUGA<br>UUUGCCUCGACGGCAAACUGGGCGCUGACGGCAACGCAC<br>UGUUCCGCCAGCCUGAUCUGCGCGAAAUGCGUGACCAGU<br>CGCAGGAAGAUCCGCGUGAAGCACAGGCUGCACAGUGGG<br>AACUGAACUACGUUGCGCUGGACGGUAACAUCGGUUGUA<br>UGGUUAACGGCGCAGGUCUGGCGAUGGGUACGAUGGACA<br>UCGUUAAACUGCACGGCGGCGAACCGGCUAACUUCCUUG<br>ACGUUGGCGGCGGCGCAACCAAAGAACGUGUAACCGAAG<br>CGUUCAAAAUCAUCCUCUCUGACGACAAAGUGAAAGCCGU<br>UCUGGUUAACAUCUUCGGCGGUAUCGUUCGUUGCGACCU<br>GAUCGCUGACGGUAUCAUCGGCGCGGUAGCAGAAGUGGG<br>UGUUAACGUACCGGUCGUGGUACGUCUGGAAGGUAACAA<br>CGCCGAACUCGGCGCGAAGAAACUGGCUGACAGCGGCCU<br>GAA**UAUU**AUUGCAGCAAAAGGUCUGACGGAUGCAGCUCA<br>GCAGGUUGUUGCCGCAGUGGAGGGGAAAUAA
	*suc*D	AUGUCCAUUUUAAUCGAUAAAAACACCAAGGUUAUCUGCC<br>AGGGCUUUACCGGUAGCCAGGGGACUUUCCACUCAGAAC<br>AGGCCAUUGCAUACGGCACUAAAAUGGUUGGUGGCGUAA<br>CUCCAGGUAAAGGCGGCACUACCCACCUCGGCCUGCCGG<br>UGUUCAACACCGUGCGUGAAGCUGUUGCUGCUACUGGCG<br>CUACCGCUUCUGUUAUCUACGUACCAGCACCGUUCUGCA<br>AAGACUCCAUUCUGGAAGCCAUCGACGCAGGCAUCAAACU<br>GAUUAUCACCAUCACUGAAGGCAUCCCGACGCUGGAUAU<br>GCUGACUGUGAAAGUGAAACUGGACGAAGCAGGCGUUCG

		UAUGAUCGGCCCGAACUGCCCAGGCGUUAUCACUCCGGG<br>UGAAUGCAAAAUCGG**UAUU**CAGCCGGGUCACAUUCACAAA<br>CCGGGUAAAGUGGGUAUCGUUUCCCGUUCCGGUACACUG<br>ACCUAUGAAGCGGUUAAACAGACCACGGAUUACGGUUUC<br>GGUCAGUCGACCUGUGUCGGUAUCGGCGGUGACCCGAUC<br>CCUGGCUCUAACUUUAUCGACAUUCUCGAAAUGUUCGAAA<br>AAGAUCCGCAGACCGAAGCGAUCGUGAUGAUUGGUGAGA<br>UCGGCGGUAGCGCUGAAGAAGAAGCGGCAGCGUACAUCA<br>AAGAGCACGUUACCAAGCCAGUUGUGGGCUACAUUGCUG<br>GUGUGACUGCGCCGAAAGGCAAACGUAUGGGCCACGCGG<br>GUGCCAUCAUUGCCGGUGGGAAAGGGACUGCGGAUGAGA<br>AAUUCGCUGCUCUGGAAGCCGCAGGCGUGAAAACCGUUC<br>GCAGCCUGGCGGAUAUCGGUGAAGCACUGAAAACUGUUC<br>UGAAAUAA
